# Green One-Pot Synthesis of Coumarin-Hydroxybenzohydrazide Hybrids and Their Antioxidant Potency

**DOI:** 10.3390/antiox10071106

**Published:** 2021-07-10

**Authors:** Marko R. Antonijević, Dušica M. Simijonović, Edina H. Avdović, Andrija Ćirić, Zorica D. Petrović, Jasmina Dimitrić Marković, Višnja Stepanić, Zoran S. Marković

**Affiliations:** 1 Department of Science, Institute for Information Technologies, University of Kragujevac, Jovana Cvijića bb, 34000 Kragujevac, Serbia; mantonijevic@uni.kg.ac.rs (M.R.A.); dusicachem@kg.ac.rs (D.M.S.); 2 Department of Chemistry, Faculty of Science, University of Kragujevac, Radoja Domanovića 12, 34000 Kragujevac, Serbia; andrija.ciric@pmf.kg.ac.rs (A.Ć.); zorica.petrovic@pmf.kg.ac.rs (Z.D.P.); 3 Faculty of Physical Chemistry, University of Belgrade, Studentski trg 12-16, 11000 Belgrade, Serbia; markovich@ffh.bg.ac.rs; 4 Ruđer Bošković Institute, Bijenička Cesta 54, 10000 Zagreb, Croatia; visnja.stepanic@irb.hr

**Keywords:** coumarins, green synthesis, antioxidants, DFT

## Abstract

Compounds from the plant world that possess antioxidant abilities are of special importance for the food and pharmaceutical industry. Coumarins are a large, widely distributed group of natural compounds, usually found in plants, often with good antioxidant capacity. The coumarin-hydroxybenzohydrazide derivatives were synthesized using a green, one-pot protocol. This procedure includes the use of an environmentally benign mixture (vinegar and ethanol) as a catalyst and solvent, as well as very easy isolation of the desired products. The obtained compounds were structurally characterized by IR and NMR spectroscopy. The purity of all compounds was determined by HPLC and by elemental microanalysis. In addition, these compounds were evaluated for their in vitro antioxidant activity. Mechanisms of antioxidative activity were theoretically investigated by the density functional theory approach and the calculated values of various thermodynamic parameters, such as bond dissociation enthalpy, proton affinity, frontier molecular orbitals, and ionization potential. In silico calculations indicated that hydrogen atom transfer and sequential proton loss–electron transfer reaction mechanisms are probable, in non-polar and polar solvents respectively. Additionally, it was found that the single-electron transfer followed by proton transfer was not an operative mechanism in either solvent. The conducted tests indicate the excellent antioxidant activity, as well as the low potential toxicity, of the investigated compounds, which makes them good candidates for potential use in food chemistry.

## 1. Introduction

The oxygen molecule is an important electron acceptor in metabolic processes in the cells of living organisms. It plays an important role in the cell’s respiratory processes, especially in a process of oxidative phosphorylation. This molecule is included in almost all electron transfer processes in organisms, and is one of the key components of the electron-transport chain, which has a crucial role in energy production [1]. However, besides its many positive effects, as a result of its bi-radical properties, it also enables the formation of partially reduced chemical species known as reactive oxygen species (ROS), which can be involved in starting a chain reaction that could be potentially dangerous for the cell [2]. The production of ROS in the organism is a natural process that allows the immune system to remove foreign bodies from blood, manages cell-signaling, leads to acceleration of the aging process, etc. ROS are constantly forming within the cells upon exposure to drugs, air pollutants, ultraviolet rays, ionizing radiation, smoke, and some endogenous metabolites of the redox and respiratory chain during the transfer of electrons. Under normal conditions, the organism is capable of regulating the production of ROS in such a manner that damage caused by influences of chemical agents on healthy cells is reduced to a minimum. The human body has developed a complex defense system against oxidative stress that includes preventive, restorative, antioxidant mechanisms, and physiological defense [3]. The natural system of antioxidant protection includes enzymatic and non-enzymatic antioxidants. Enzymatic antioxidants consist of a limited number of proteins such as catalase, glutathione peroxidase, superoxide dismutase, along with some auxiliary enzymes. They work by converting reactive species into hydrogen peroxide and later into water, in the presence of cofactors such as copper, zinc, manganese, and iron. Another group of antioxidants can be divided into direct and indirect, depending on the mechanism of action. Direct-acting antioxidants are extremely important in the body’s defense against oxidative stress. Most of them are taken into the body through diet, and only a small number are synthesized in the body itself. These include vitamins, polyphenols, carotenoids, etc. Most of the naturally occurring antioxidants are found in the plant world, often in plants used in traditional medicine. Extraction, identification, and evaluation of the biological activity of these compounds has been a hot topic in the last couple of decades [4,5,6].

Despite the great efforts of many researchers over many years, creating new and potent antioxidant agents, which will also satisfy other criteria, like solubility and selective biological activity/toxicity, is quite challenging. Coumarins are relatively simple, naturally occurring phenolic compounds consisting of fused γ-pyrone and benzene rings, along with the carbonyl group on the pyrone ring at position C2. They are found in some essential oils like lavender oil, cinnamon bark oil, and cassia leaf oil. They mostly occur in higher plants, in the fruits, followed by the roots, stems, and leaves. The most frequent source for human exposure to coumarins is certain types of cinnamon. Coumarins have important biological functions, such as growth regulation, respiratory control, protection against herbivores and microorganisms. Simple coumarins act as hormones and signaling molecules. Over the last decade, antioxidant, anti-inflammatory, anticoagulant, enzyme inhibitory, antimicrobial, and anticancer activities of coumarins have been widely investigated. Some of them, like novobiocin and armillarisin A (antibiotics), warfarin, phenprocoumon, and acenocoumarol (anticoagulants), and hymecromone (antispasmodic and choleretic) have been in clinical use for many years [7,8,9,10,11,12,13,14,15].

Synthetic coumarins have great potential for developing new compounds with the aforementioned desired activities. It is worth pointing out that the reactions regarding 3-acetyl-4-hydroxycoumarin and various benzoyl hydrazides have been described in only a few articles [16,17,18]. The synthesis of coumarin-hydrazide derivatives was achieved via condensation of 3-acetyl-4-hydroxycoumarin with appropriate hydrazides under reflux in *n*-propanol or by heating in ethanol and in the presence of acetic acid as catalyst. Additionally, in these studies, the isolated products were evaluated for their antioxidant, anti-LOX, and anticancer activity. The investigated compounds exhibited good to moderate cytotoxicity and anti-inflammatory activity, while the radical scavenging activity was bad. 

The aim of this research is the synthesis of new coumarin hydroxybenzohydrazide derivatives with expected good antioxidant activity. For this synthesis green, simple and low-cost protocol was used. The synthesized compounds are structurally characterized, and their in vitro antiradical activity is investigated. Since they can be potent antioxidants, a theoretical investigation of the structure and reaction mechanisms involved in free radical scavenging is performed. DFT approach allowed the computation of various thermodynamic properties, such as bond dissociation enthalpy (BDE), proton affinity (PA), frontier molecular orbitals and HOMO-LUMO gap, and ionization potential (IP), to predict the mechanisms of antioxidative action.

## 2. Materials and Methods

### 2.1. Chemical Reagents and Instruments

Chemicals (purity > 98%) used for the synthesis were acquired from Merck, and vinegar (acetic acid 90 g/L) was purchased from the local market. Compounds 3-acetyl-4-hydroxycoumarin (**1**) and hydrazides (**2**) were prepared according to the previously reported methods [19,20]. All chemicals used in HPLC analysis were HPLC grade.

IR spectroscopy was performed on a Perkin-Elmer Spectrum One FT-IR spectrometer using the KBr disc. The NMR spectra were recorded on a Varian Gemini spectrometer (200 MHz for ^1^H and 50 MHz for ^13^C) in DMSO-*d*_6_. The UV-Vis measurements were recorded on Agilent Technologies, Cary 300 Series UV/Vis Spectrophotometer. Melting points were determined on a Mel-Temp capillary melting points apparatus, model 1001. Elemental microanalysis for carbon, hydrogen, and nitrogen was done in the Centre for Instrumental Analysis, at the Faculty of Chemistry, Belgrade. Elemental (C, H, N) analysis of the samples was carried out on an Elemental analysis system VARIO EL III CHNOS, model—Elementar Analysensysteme GmbH, 2003. To confirm the purity of isolated products, HPLC analyses were performed using an HPLC system (Shimadzu Prominence, Kyoto, Japan) with a PDA detector (SPD-M20A). The separation was carried out using a Hypersil Gold aQ C18 column (150 × 4.6 mm, 5 µm) at a flow rate of 1 mL/min. Mobile phases: (A) 0.1% formic acid in water, (B) 0.1% formic acid in acetonitrile. Gradient profile 0–2 min 2% B, 45 min 95% B. 

### 2.2. Synthesis of Coumarin-Hydroxybenzohydrazide Derivatives

The starting compounds 3-acetyl-4-hydroxycoumarin (**1**) (1 mmoL) and benzoyl hydrazides (**2**) (1 mmoL), were dissolved in a mixture of vinegar and ethanol (1:1) (20 mL) and stirred at reflux for 5 h. Reaction progress was monitored using thin-layer chromatography (TLC). When the reaction was completed, the resulting mixture was cooled to room temperature and the precipitate was collected by filtration. All of the coumarin-hydroxybenzohydrazide products (**3a**–**e**) were characterized with melting points, ^1^H NMR, ^13^C NMR, and IR spectra, as well as with elemental and HPLC analysis. 

*(E)-N’-(1-(2,4-dioxochroman-3-ylidene)ethyl)-2-hydroxybenzohydrazide* (**3a**). Beige solid, m.p. 248–249 °C; Isolated yield: 0.221 g (65.31%), HPLC purity: 91.44%; ^1^H NMR (200 MHz, DMSO-*d*_6_) δ: 15.95 (s, 1H), 8.00 (dd, *J* = 7.8, 1.6 Hz, 1H), 7.87 (dd, *J* = 7.8, 1.7 Hz, 1H), 7.67 (m, 1H), 7.53–7.40 (m, 1H), 7.40–7.25 (m, 2H), 7.07–6.90 (m, 2H), 2.74 (s, 3H); ^13^C NMR (50 MHz, DMSO-*d*_6_) δ: 178.8, 170.5, 164.3, 161.5, 157.5, 153.1, 134.3, 134.1, 129.9, 125.7, 123.9, 119.6, 119.5, 117.1, 116.4, 116.3, 95.4, 17.6; IR (KBr): 3521 (OH), 3435, 3267 (NH), 3049 (=CH), 2930, 2862 (CH), 1692, 1629, 1608 (C=O), 1547, 1455 (C=C), 1233 (C–O) cm^−1^; C_18_H_14_N_2_O_5_ (FW = 338.32): C, 63.90; N, 8.28; H, 4.17%; found: C, 63.74; N, 8.45; H, 4.28%.

*(E)-N’-(1-(2,4-dioxochroman-3-ylidene)ethyl)-4-hydroxybenzohydrazide* (**3b**).White solid, m.p. 264–265 °C; Isolated yield: 0.215 g (63.51%), HPLC purity: 97.79%; ^1^H NMR (200 MHz, DMSO-*d*_6_) δ: 15.72 (s, 1H), 11.56 (s, 1H), 10.34 (s, 1H), 7.99 (dd, *J* = 7.8, 1.6 Hz, 1H), 7.90–7.78 (m, 2H), 7.73–7.57 (m, 1H), 7.39–7.23 (m, 2H), 6.96–6.85 (m, 2H), 2.74 (s, 3H); ^13^C NMR (50 MHz, DMSO-*d*_6_) δ: 179.2, 171.8, 164.6, 161.6, 161.5, 153.2, 134.3, 130.1, 125.7, 123.9, 121.6, 119.8, 116.4, 115.4, 95.3, 17.8; IR (KBr): 3565 (OH), 3435, 3183 (NH), 3057 (=CH), 2964, 2807 (CH), 1666, 1639, 1605 (C=O), 1566, 1490, 1467(C=C), 1232 (C–O) cm^−1^; C_18_H_14_N_2_O_5_ (FW = 338.32): C, 63.90; N, 8.28; H, 4.17%; found: C, 63.68; N, 8.40; H, 4.37%.

*(E)-N’-(1-(2,4-dioxochroman-3-ylidene)ethyl)-4-hydroxy-3-methoxybenzohydrazide* (**3c**). Light yellow solid, m.p. 223–224 °C; Isolated yield: 0.246 g (66.83%), HPLC purity: 98.50%; ^1^H NMR (200 MHz, DMSO-*d*_6_) δ: 15.67 (s, 1H), 11.55 (s, 1H), 9.96 (s, 1H), 8.00 (dd, *J* = 7.8, 1.3 Hz, 1H), 7.95 (m, 1H), 7.67 (ddd, *J* = 8.1, 7.4, 1.7 Hz, 1H), 7.54–7.43 (m, 2H), 7.32 (ddd, *J* = 8.0, 5.3, 1.6 Hz, 2H), 6.92 (d, *J* = 11.3 Hz, 1H), 3.86 (s, 3H), 2.75 (s, 3H); ^13^C NMR (50 MHz, DMSO-*d*_6_) δ: 179.3, 175.9, 172.2, 164.6, 161.6, 153.2, 151.1, 147.5, 134.4, 125.8, 123.9, 122.0, 121.8, 119.8, 116.4, 115.3, 111.9, 95.4, 55.9, 17.9; IR (KBr): 3524 (OH), 3388, 3235 (NH), 3090 (=CH), 2944, 2782 (CH), 1675, 1605, 1591 (C=O), 1527, 1484, 1410 (C=C), 1221 (C–O) cm^−1^; C_19_H_16_N_2_O_6_ (FW = 368.10): C, 61.96; N, 7.61; H, 4.38%; found: C, 61.62; N, 7.43; H, 4.17%.

*(E)-N’-(1-(2,4-dioxochroman-3-ylidene)ethyl)-2,3-dihydroxybenzohydrazide* (**3d**). Beige solid, m.p. 245–247 °C; Isolated yield: 0.223 g (62.85%), HPLC purity: 99.10%; ^1^H NMR (200 MHz, DMSO-*d*_6_) δ: 15.88 (s, 1H), 8.00 (dd, *J* = 7.8, 1.4 Hz, 1H), 7.76–7.58 (m, 1H), 7.47–7.20 (m, 3H), 7.02 (dd, *J* = 7.8, 1.6 Hz, 1H), 6.80 (t, *J* = 7.9 Hz, 1H), 2.73 (s, 3H); ^13^C NMR (50 MHz, DMSO-*d*_6_) δ: 178.9, 171.0, 165.5, 161.5, 153.2, 147.2, 146.2, 134.4, 125.7, 124.0, 119.6, 119.4, 119.1, 119.1, 116.4, 116.2, 95.5, 17.6; IR (KBr): 3498 (OH), 3386, 3269 (NH), 3050 (=CH), 2964, 2863 (CH), 1703, 1659, 1609 (C=O), 1583, 1524, 1484, 1466 (C=C), 1222 (C–O) cm^−1^; C_18_H_14_N_2_O_6_ (FW = 354.32): C, 61.02; N, 7.91; H, 3.98%; found: C, 61.22; N, 7.79; H, 3.67%.

*(E)-N’-(1-(2,4-dioxochroman-3-ylidene)ethyl)-3,4-dihydroxybenzohydrazide* (**3e**). Beige solid, m.p. 255–257 °C; Isolated yield: 0.233 g (65.71%), HPLC purity: 97.46%; ^1^H NMR (200 MHz, DMSO-*d*_6_) δ: 15.71 (s, 1H), 11.51 (s, 1H), 9.86 (s, 1H), 9.45 (s, 1H), 7.99 (dd, *J* = 7.9, 1.5 Hz, 1H), 7.66 (ddd, *J* = 8.1, 7.4, 1.7 Hz, 1H), 7.44–7.14 (m, 4H), 6.87 (d, *J* = 8.1 Hz, 1H), 2.73 (s, 3H); ^13^C NMR (50 MHz, DMSO-*d*_6_) δ: 179.2, 171.6, 164.7, 161.6, 153.2, 150.1, 145.3, 134.3, 125.7, 123.9, 121.9, 120.3, 119.8, 116.4, 115.4, 115.4, 95.3, 17.8; IR (KBr): 3478 (OH), 3419, 3156 (NH), 3066 (=CH), 2973 (CH), 1668, 1641, 1602 (C=O), 1568, 1489, 1457 (C=C), 1222 (C–O) cm^−1^. C_18_H_14_N_2_O_6_ (FW = 354.32): C, 61.02; N, 7.91; H, 3.98%; found: C, 61.37; N, 7.73; H, 3.69%.

### 2.3. DPPH Radical Scavenging Assay

The free radical scavenging activities of obtained products were determined by 2,2-diphenyl-1-picrylhydrazyl (DPPH) assay [21]. In detail, the tested compounds (20 µL of different concentrations dissolved in DMSO and 980 mL of methanol) were mixed with an equal volume of the solution of DPPH in methanol (0.05 mM). The prepared samples were shaken well and left at room temperature in the dark for 20 and 60 min. After the incubation period absorbance was determined at 517 nm by using the methanol as a blank control. All tests were run in triplicate and averaged. Nordihydroguaiaretic acid (NDGA) and quercetin were used as positive controls. For products that exert good activity, IC_50_ values were determined. Detailed calculations of IC_50_ value for the most active compound is given in the Supplementary Material. The results are presented as mean ± SD. For calculation of the stoichiometric factor [22,23], the following equation was used:

$$stoichiometric\;factor=\frac{{\left[DPPH\right]}_{0}}{\left(2\times IC_{50}\right)}$$

### 2.4. Computational Methods

The calculations were carried out by employing the *Gaussian09* software package [24]. For optimization of neutral molecules, the corresponding radicals, anions, and radical cations quantum chemical calculations based on density functional theory (DFT) were used. Since it has been shown that the M062-X method well describes short-range and medium-range intramolecular and intermolecular interactions (<500 pm) [25], the M06-2X method is applied in combination with 6–311++G(d,p) basis set. This theoretical model is suitable for thermodynamic and kinetic investigation of reaction mechanisms of examined compounds with free radicals [22,26]. All investigated structures were reoptimized in methanol (ε = 32.61), and benzene (ε = 2.27). The solvent effect was included by the Conductor-like Polarizable Continuum Model (CPCM) [27], without any geometrical constraints. Solvents were selected to simulate the polar and non-polar environments, as well as conditions of experimental measurements. 

Three antioxidant mechanisms were selected for the evaluation of the antioxidant activity: Hydrogen Atom Transfer (HAT), Single-Electron Transfer followed by Proton Transfer (SET-PT), and Sequential Proton Loss-Electron Transfer (SPLET) [28,29,30,31].

The abstraction of hydrogen atoms can be described by two mechanisms, HAT and Proton-Coupled Electron Transfer (PCET) [29]. The HAT mechanism is defined as the simultaneous transfer of an electron and a proton between the donor and acceptor. On the other hand, the PCET mechanism involves a significant redistribution of molecular charge, during the transfer of hydrogen atoms. In this study, only the HAT mechanism was considered because only thermodynamic parameters were used to test antioxidant capacity. It should be noted that both reaction pathways are necessary for the kinetic examination of the mechanisms of antioxidant activity. In the HAT mechanism, the homolytic cleavage of the O-H bond occurs leading to the separation between radical species (Ar-O^•^) and a hydrogen atom (Equation (1)) [30,31].

Ar–OH → Ar–O^•^ + H^•^ (1)

The SET-PT mechanism is a two-step process that includes electron loss from a molecule and generation of the radical cation (Ar–OH^+•^), which in the second step loses protons (Equation (2a,b)).

Ar–OH → Ar–OH^•+^ + e^−^ (2a)

Ar–OH^•+^→ Ar–O^•^ + H^+^ (2b)

The SPLET mechanism is also a two-step mechanism. In the first step, an anion (Ar–O^−^) is formed from the antioxidant molecule, while the corresponding radical is formed after the electron transfer in the second step of this mechanism (Equation (3a,b)).

Ar–OH → Ar–O^−^ + H^+^ (3a)

Ar–O^−^ → Ar–O^•^ + e^−^ (3b)

The thermodynamic parameters governing the mentioned antioxidative mechanisms are BDE (Bond Dissociation Enthalpy) describing HAT, PA (Proton Affinity), and ETE (Electron Transfer Enthalpy) describing SPLET, and IP (Ionization Potential) and PDE (Proton Dissociation Enthalpy) describing SET-PT mechanism. The contribution of these parameters was calculated from the enthalpies of the optimized chemical species using the following equations [32,33]:

BDE = *H*(Ar–O**^•^**) + *H*(H**^•^**) − *H*(Ar–OH) (4)

IP = *H*(Ar–OH**^•+^**) + *H*(e^−^) − *H*(Ar–OH) (5a)

PDE = *H*(Ar–O**^•^**) + *H*(H**^+^**) − *H*(Ar–OH**^•+^**) (5b)

PA = *H*(Ar–O^−^) + *H*(H**^+^**) − *H*(Ar–OH) (6a)

ETE = *H*(Ar–O**^•^**) + *H*(e^−^) − *H*(Ar–O^−^) (6b)

All reaction enthalpies used in the equations were calculated at 298 K. The calculated enthalpy values of the solvated proton, *H*(H**^+^**) and electron, *H*(e^−^), for the M062-X method in benzene (−856.9 and –11.9 kJ moL^−1^) and methanol (−1010.5 and −93.5 kJ moL^−1^) were taken from the literature [28,34].

The free radical scavenging potency of the synthesized coumarin derivatives toward the DPPH radical was investigated in two different solvents: benzene and methanol. Mechanisms of radical scavenging activity are described by Equations (1s)–(5s), given in the Supplementary Material. Thermodynamic parameters of the investigated mechanisms were calculated according to Equations (6s)–(10s) from Supplementary material.

## 3. Results and Discussion

### 3.1. Synthesis

**Scheme 1:**
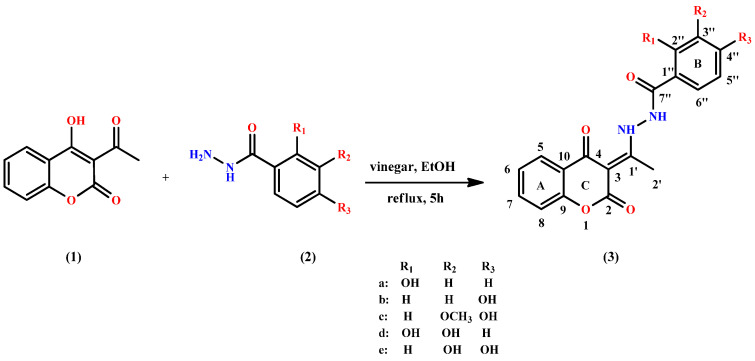
Synthesis of coumarin-hydroxybenzohydrazide derivatives.

The synthesis of coumarin-hydroxybenzohydrazide derivatives (**3**) was achieved by treatment of 3-acetyl-4-hydroxycoumarin (**1**) with corresponding benzoyl hydrazides (**2a–e**), Scheme 1. The reaction medium for these reactions was a mixture of vinegar and ethanol (1:1). This medium provided good solubility of the reactants and acted as a catalyst. The coumarin-hydroxybenzohydrazide derivatives **3a**–**e** were obtained in moderate yields under reflux for 5 h. All products **3a**–**e** were precipitated during the reaction and isolated by filtration. Three of five obtained products (**3c**–**e**) are reported in this study for the first time. 

The synthesized coumarin-hydroxybenzohydrazide derivatives (**3**) were characterized using IR, ^1^H and ^13^C NMR spectroscopy, as well as with melting points. The purity of all isolated compounds was determined by HPLC and elemental analysis. The coumarin-hydroxybenzohydrazide derivatives (**3**) were obtained in yields 62.85–66.83% and with HPLC purity in the range 91.44–99.10%, Table 1. HPLC data and chromatograms can be found in Supplementary Material, Tables S1–S5 and Figures S11–S15, respectively.

The 2,4-dione tautomeric form of these derivatives was assumed on the basis of the obtained spectroscopy data and X-ray structure which was determined for a similar type of compounds in our previous studies [35,36,37].

All synthesized compounds were characterized by IR spectroscopy. The obtained spectra are very similar to each other. In all spectra, bands of about 3500 cm^−1^ belonging to phenolic OH groups are observed, while bands of NH groups appear at lower wavenumbers, in the range of 3200–3400 cm^−1^. Bands in the region 1700–1600 cm^−1^ are assigned to different carbonyl groups. The stretching vibrations corresponding to the C-O group were identified between 1220 and 1230 cm^−1^.

The NMR spectra of coumarin-hydroxybenzohydrazide derivatives presented in the Supplementary Material (Figures S1–S10) are in good agreement with the proposed structures. In the ^1^H NMR spectra of all obtained compounds (**3a**–**e**) characteristic singlets positioned at 2.73–2.75 ppm are assigned to the protons of the methyl group in position C2′. In addition, the broad singlet appearing in the 9.45–10.34 ppm range is assigned to phenolic protons, and the singlets positioned at a higher chemical shift, between 11.32–11.56 and 15.67–15.95 ppm, are assigned to two NH groups. The NMR spectra clearly show multiple resonance maxima of aromatic protons of the 2,4-dioxochroman and benzoyl moieties in the range of 6.80–8.00 ppm. In addition, the singlet at 3.86 ppm in the spectrum of compound **3c** is assigned to the proton of the methoxy group attached to the aromatic ring of the hydrazide moiety.

In the ^13^C NMR spectra of compound **3a**–**e**, the peak at about 18 ppm was assigned to the carbon atom of the methyl group in position C2′. The peak at about 95 ppm is attributed to the C3 atom. The aromatic carbons of both parts have resonance maxima in the 112–153 ppm region. The resonance maxima with the highest chemical shifts, at about 179 ppm, originate from carbon atoms of the C4-carbonyl group, while the signals of the amide and lactone carbonyl groups occur at lower chemical shifts, at about 161–165 ppm. The C1 atom showed a resonance in the range of 171–176 ppm. 

**Scheme 2:**
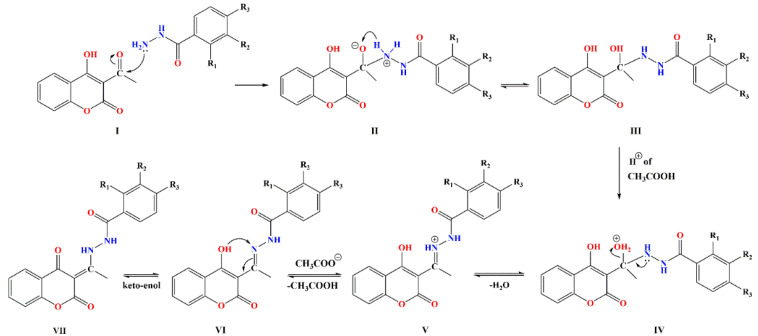
The suggested mechanism of the formation of coumarin-hydroxybenzohydrazide hybrids by the reaction between 3-acetyl-4-hydroxycoumarin and benzoyl hydrazides.

The predicted mechanism of synthesis of new coumarin-hydrazide hybrids is presented in Scheme 2. The first step of the reaction is the nucleophilic attack of the hydrazide on the carbon of the carbonyl group of coumarin (**I**), whereby intermediate (**II**) is formed. In the next step, the proton from the NH_2_ group is transferred to a partially negative oxygen atom of the C=O group, which is followed by the formation of a hemiaminal (**III**). In the further course of the reaction, the protonation of the -OH group takes place in the presence of acetic acid, which is followed by releasing of the water molecules (**IV**). Deprotonation of the iminium ion (**V**) leads to the formation of the enol form of the coumarin-hydroxybenzohydrazide hybrids (**VI**). In the last step, by acid-catalyzed keto-enol tautomerization, a more stable keto form (**VII**) is obtained. The confirmation of this reaction path is supported by the fact that ^1^H NMR spectra for all obtained compounds showed signals at about 15 ppm, which originate from the proton -NH group [35,36,37].

### 3.2. In Vitro Antioxidative Activity of Tested Compounds

The results of the in vitro DPPH test are presented in Table 2. The obtained results show that two out of five investigated compounds, **3d**, and **3e**, expressed high antioxidant activity, with IC_50_ values of 2.9 and 12.9 µM, respectively. It should be mentioned that the compound **3d** reduced DPPH well and exhibited high activity, slightly lower than the reference compounds NDGA and quercetin. The compound **3e** also showed high antioxidative activity, a little bit lower than compound **3d**. Namely, based on the obtained results, the new compounds **3d** and **3e** can be considered good antioxidants. Additionally, the stoichiometric factor (SF) of **3d**, which is equal to 4.4, indicates very good antioxidative ability, taking into account the fact that significant radical scavengers have SF bigger than 2 [22,23,38,39].

The obtained results for compounds **3a–c** revealed that the presence of only one phenolic OH group had no significant effect on the DPPH radical scavenging activity. However, for compounds **3d** and **3e**, with additional phenolic OH groups in neighboring positions, a significant improvement of antioxidative activity was observed. This is in agreement with the fact that the pronounced antioxidative capacity of compounds with catechol moiety stems from resonance and electron-donating effects of these groups, which increase the stability of the formed phenoxy radical. In these compounds, the hydroxy group supports the homolytic cleavage of the neighboring O–H bond and enables the formation of a hydrogen bond with formed phenoxy radical [40,41,42]. 

In addition, appropriate in silico methods were used to determine the most likely mechanism of radical scavenging activity of coumarin-hydroxybenzohydrazide derivatives (**3**).

### 3.3. Thermodynamic Parameters of Antioxidative Activity

Geometry optimization of the examined compounds was performed using the M062X/6-311++G(d,p) model. The most stable conformations of **3a**–**e** are shown in Figure S16. The obtained values for bond lengths, bond angles, and dihedral angles are shown in Tables S8–S10. To confirm that the obtained geometries were the minimums on the potential energy surface, the vibration frequencies were calculated at the same theoretical level. This means that the corresponding stationary points were without negative eigenvalues in the force constant matrix. From the optimized molecular structure of the five coumarin derivatives, it can be seen that the N-N bond is a partial double bond that exists in all compounds and provides delocalization of the π-electron between the coumarin base and the aromatic ring (B), which contributes to the stabilization of the formed radicals after H-abstraction. Compared to all phenol-hydroxy and amino groups on all considered molecules **3a**–**e**, the bond length C1′N-H is noticeably longer than C7″N-H. This may be associated with a ketone group at the C4 position that forms an HB between C1′NH and O4, resulting in an extension of the bond length of the C1′N-H bond. Alongside this hydrogen bond, compounds **3c**–**e** are capable of establishing additional hydrogen bonds, due to the fact that they possess additional OH groups in different positions. The additional hydrogen bond in **3c** is located between the C4″-OH and C3″-OCH_3_ groups, while there are three HB in **3d**, from which two are formed between C2″-OH, the adjacent carbonyl group and C3″-OH group. There are only two HBs in compound **3e**. Besides C1′NH-O4, an HB is formed in the catechol moiety with adjacent C3″OH-O4″. The number and position of the hydrogen bonds play a major role in the stabilization of the molecule and consequently have an impact on its antioxidative capacity [43]. Careful analysis of the structures of the considered compounds showed that benzopyrone and phenyl rings were not in the planar conformation. The calculated dihedral angles C-N-N-C were found in the interval between 74.71°–76.51° for all scrutinized compounds. These values clearly show that the examined compounds lose coplanarity to varying degrees. These results indicate the weakening of the electronic distribution within the compounds, which significantly affects their antioxidant activity.

#### 3.3.1. Bond Dissociation Energy

The BDE values for OH and NH groups are known to be of particular importance for understanding the mechanism of free radical scavenging activity [44,45]. This means that a weak O-H or N-H bond has a rapid response and thus potential for antioxidative activity. The capacity of coumarin-hydroxybenzohydrazide derivatives to remove free radicals is generally related to the existence of OH and NH groups in a particular position on the core of coumarin-hydroxybenzohydrazide derivatives. Optimization of the geometry of radical species formed after homolytic cleavage of either OH or NH bonds for all coumarin-hydroxybenzohydrazide derivatives is performed starting from the optimized geometry of neutral molecules. Removal of H-atoms from the C2″-OH, C3″-OH, C4″-OH, and C7″-NH positions in coumarin-hydroxybenzohydrazide derivatives yields various radical species. For example, the radical species created by removing the H-atom from the C2″-OH group of **3a** is called **3a**C2″O^•^. The remaining radical species are generated and named in the same way. It should be noted that the geometry optimization of free radical species was performed using the same theoretical model and in the same solvents as for the parent molecules.

The calculated BDE values for all coumarin-hydroxybenzohydrazide derivatives are shown in Table 3. From the calculated values, it can be seen that the smallest BDE values are obtained for the C7″-NH position, in both solvents, for all investigated molecules, except in the case of compound **3e**, where slightly lower BDE values are obtained for the OH group in the para position. Such BDE values for **3e** are a consequence of catechol structure and the formation of intramolecular hydrogen bonds between both, adjacent OH groups and the resulting radicals with the adjacent OH group. In addition, the BDE values of the neighboring OH groups in **3d** are somewhat higher, despite the fact that the groups are in *o*- and *m*-positions.

**Figure 1:**
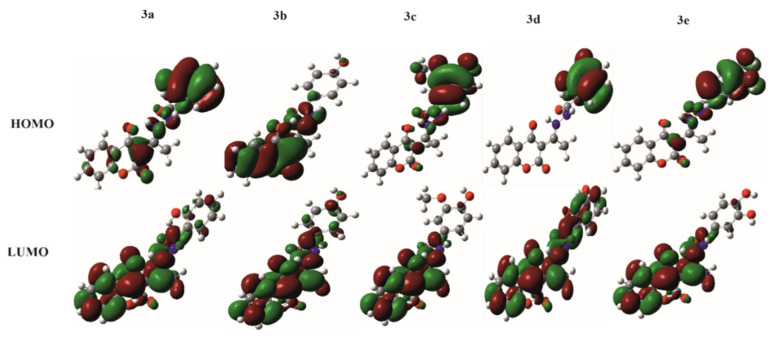
Frontier orbitals of the investigated compounds.

This is a consequence of better delocalization of the unpaired electron in the **3e**C4″O^•^ than in the **3d**C2″O^•^ radical (Figure 1). However, it should be mentioned that BDE values for the position C7″-NH, calculated in benzene, are a little bit lower than those calculated in methanol are. 

#### 3.3.2. Proton Affinity

The heterolytic cleavage of N-H and O-H bonds leads to the formation of the corresponding anions. This process is considered to be the first step of the SPLET mechanistic pathway and is described by the thermodynamic parameter PA. The high calculated PA values indicate that the SPLET mechanism is not operative in non-polar conditions. On the other hand, PA values obtained in methanol were significantly lower, due to increased anion stabilization in polar solvents. This is a consequence of the fact that polar protic solvents stabilize anions via hydrogen bonding. The obtained results are presented in Table 3. By analyzing the PA values, it is clear that the abstraction of the proton from nitrogen is much easier than from an oxygen atom. The lowest PA value, found for **3d**, implies that this compound is the most reactive when operating following the SPLET mechanism. Such results are in good agreement with the results obtained in vitro. 

#### 3.3.3. Frontier Molecular Orbitals and HOMO–LUMO Gap

Investigating the energies of the frontier molecular orbitals is very important for understanding the chemical behavior of tested compounds, because these orbitals usually take part in reactions. The ability of molecules with respect to electron donation or electron affinity can be determined using HOMO or LUMO energy values. The highest occupied molecular orbital (HOMO) usually indicates that the molecule is a good electron donor, and characterizes the susceptibility of the molecule to attack by electrophiles. The energy of the lowest unoccupied molecular orbital (LUMO) is related to electron affinity and describes the sensitivity of molecules toward nucleophile attack. The HOMO–LUMO gap (H-L), calculated as the energy difference between the HOMO and LUMO orbitals, is an important index of stability and chemical reactivity. A high value of H-L implies high stability of molecules and lower reactivity in chemical reactions, while a low value of H-L implies low stability and higher molecular reactivity [22,33].

The frontier orbitals for the investigated compounds in methanol are shown in Figure 1, and the frontier orbital energies, as well as H-L, are indicated in Table 4. The active site can be visually demonstrated by the distribution of the frontier orbital. Based on the results presented in Figure 1, it is obvious that the HOMO of almost all investigated compounds is mainly distributed in the aromatic ring of benzoyl-hydrazide, while LUMO is mainly assigned in the coumarin part of the molecule. The electron donation ability of a molecule can be determined by HOMO values; HOMO with high energy corresponds to a strong electron donation ability. From Table 4, it can be seen that **3c** possesses the highest HOMO among all compounds, while **3a** possesses the lowest one. The H-L of **3d**, in methanol, is 6.39 eV, while for the least reactive **3b** it is 6.74 eV. Values for the remaining compounds lie in the range of 0.35 eV. It should be noted that almost identical values are obtained for the H-L energy in the nonpolar solvent. The lower the value of H-L, the more pronounced the antioxidant abilities of the molecules. The relatively low energy of H-L indicates that the scrutinized compounds could be a highly reactive system. The obtained values for the H-L are in accordance with the in vitro results obtained by the DPPH test (Table 2).

When the HOMO orbital is localized on the B ring (phenol/catechol part of the molecule), as is the case for the compounds **3e**, **3d**, and **3c**, values of the H-L, as well as the *E*_HOMO_, are higher than in the case of the compounds **3a** and **3b**. Therefore, compounds **3e**, **3d**, and **3c** are more reactive than compounds **3a** and **3b**. The difference in HOMO distribution between **3a** and **3b** is a consequence of HB forming between OH group in position C2″-OH and carbonyl group in position C7″. Besides the position of the OH group and its ability to form HB, several electron-rich substituents on the B ring play an important part in HOMO and LUMO distribution, as well as the values of the H-L. As seen from Figure 1, HOMO tends to be localized on the part of the molecule with electron-rich substituents, because of the increased electron delocalization. This is why **3c**, **3d**, and **3e** have HOMO distributed only over the B ring, which makes the HOMO orbital less stable, thus making the H-L smaller. Expectedly, compounds with a smaller HL-gap are more reactive towards free radical species. 

The data presented in Table 4 suggest that compound **3d** has the highest antioxidative potential since it has the lowest value of H-L. 

#### 3.3.4. Ionization Potential

Removal of free radicals can also be achieved by donating one electron from the parent compound to the free radical, which is followed by the formation of a radical cation. The ability of the investigated compound to donate an electron is associated with prolonged electron delocalization across the entire molecule. It is known that many natural products such as flavonoids, which have a high degree of π-delocalization, are more active as radical scavengers. The value that measures this ability is IP. The calculated IP values for the investigated compounds in methanol and benzene are given in Table 3.

Generally speaking, molecules with lower IP values are more easily engaged in radical-scavenging reactions [46,47]. All investigated compounds show mutually similar and high values for this thermodynamic parameter (Table 3). Based on the presented values for IP, it can be concluded that this mechanistic pathway is unlikely, and should be excluded from further discussion.

### 3.4. Theoretical Assessment of Mechanisms of Antioxidative Action

Based on the values of BDE, IP, PDE, PA, and ETE, the dominant mechanism of antioxidant action of coumarin-hydroxybenzohydrazide derivatives **3a**–**e** can be assumed. The thermodynamically most preferable mechanism is the one with the lowest value for the parameter describing the first step. All thermodynamic parameters for the examined coumarin-hydroxybenzohydrazide derivatives **3a**–**e** were calculated by applying the same level of theory in benzene and methanol as solvents (Table 3). The obtained values of thermodynamic parameters indicate that the good antioxidative activity of these compounds can be expected. It was found that PA values in methanol were considerably lower than the corresponding BDEs. This implies that SPLET is the predominant mechanism of antioxidant action in a polar environment. The obtained PA values suggest that compound **3d** shows the highest antioxidative capacity, which is in good agreement with IC_50_ values (Table 2). On the other hand, the values of BDE and PA (Table 3) indicate the competition of HAT and SPLET mechanisms in a non-polar medium. 

### 3.5. Radicals and Anions of Investigated Compounds

**Figure 2:**
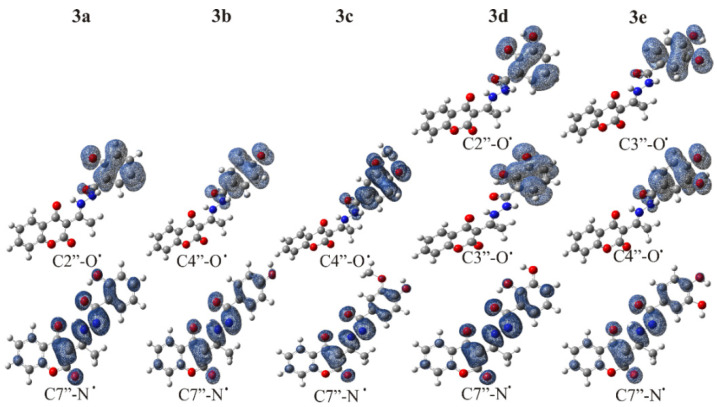
Spin density maps of the radicals obtained in the radical scavenging reactions of investigated compounds.

The potential antioxidative action of the investigated compounds requires homolytic and heterolytic bond cleavage between the hydrogen and heteroatom. This leads to the formation of corresponding radicals and anions. The relative stability of these chemical species depends on their ability for the delocalization of unpaired electrons or charges. Depending on their relative stability, it can be determined which functional group or position is the most likely to contribute to the antioxidant activity, i.e., to the ROS inactivation. According to the thermodynamic parameters presented in Table 3, radical species derived from NH groups are generally more stable than those formed by homolytic cleavage of the OH bond. This behavior can be explained by examining the radical structures of the investigated compounds presented in Figure 2. Radicals formed by removing hydrogen atoms from the nitrogen of the hydrazide group are more stable. The main reason for this lies in the fact that nitrogen atom rehybridizes from sp^3^ to sp^2^ hybrid, which causes the investigated compounds to become planar. 

The planar conformation enables the delocalization of the spin over the whole molecule (Figure 2), and better delocalization leads to the higher radical stability of all investigated compounds. On the other hand, in cases where the antioxidant action is achieved through the OH group, the spin is delocalized only on the B ring of the molecule. 

The maps of electrostatic potentials (ESP) of the investigated anions show that in the planar conformation, negative charge is better delocalized than in the non-planar one (Figure S18). As the atomic charges of ESP are important for understanding the second step of the SPLET mechanism, ESP maps of all coumarin hybrids were calculated to verify high electron density regions that can indicate the atoms responsible for the next step and, consequently, the electron transfer. Figure S17 shows the ESP maps of the tested compounds, while Figure S18 shows ESP maps of the corresponding anions.

**Figure 3:**
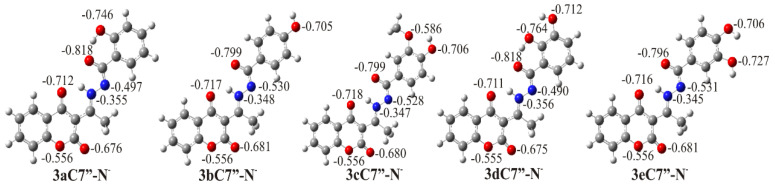
The NBO charge delocalization for anions obtained by proton abstraction from -NH group in position C7″-N for investigated compounds.

It can be seen, from Figure S18, that a localized homogeneous negative surface (represented in red) is on the atom from which the single proton was abstracted. The best delocalization effect was found for the nitrogen atom anion in position C7″-N^−^, which delocalizes its negative charge through the oxygen atom on the carbonyl group and the corresponding oxygen atoms on the B ring. Delocalization of this type of negative charge is presented in Figure 3.

### 3.6. Radical Scavenging Activity against DPPH Radical

The antioxidative capacity of the investigated coumarin-hydroxybenzohydrazide derivatives was examined by the DPPH test, and the results are presented in Table 2. Since the IC_50_ values represent the sample concentration required to inhibit 50% of DPPH radicals, thermodynamic calculation on possible inactivation of DPPH radical through already examined free radical scavenging mechanisms was performed. The potential mechanisms of free radical scavenging were discussed based on a change in enthalpies for reactions following HAT, SET-PT and SPLET mechanisms. The calculations were performed in methanol, simulating the environment in which the in vitro DPPH test was performed. The energy difference between the products and reactants of the reaction was used as the principal to assess whether the reaction can be expected to occur or not. The lowest value of thermodynamic parameters implies the preferability of the mechanistic pathway.

Analysis of the results presented in Table 5 exposes positive **∆*H*_IP_** values for reaction with all investigated compounds. This means that all the examined reactions of scavenging of DPPH radical via SET-PT are endergonic. The high values of **∆*H*_IP_** unequivocally indicate that the SET-PT mechanism can be neglected when scavenging of DPPH radical is discussed, which is consistent with the thermodynamic results presented in Table 3. Furthermore, the HAT and SPLET mechanisms will be considered possible mechanisms of scavenging the DPPH radical. Both HAT and SPLET mechanisms can be discussed as potential mechanisms of radical scavenging in methanol, since low positive values (<50 kJ/moL) are obtained. The obtained values of **∆*H*_BDE_** and **∆*H*_PA_** for all examined compounds are similar and indicate endogenic reactions, except in the case of the radical, e.g., the anion in the C7″-NH position. Namely, in the C7″-NH position, negative values of **∆*H*_PA_** are obtained, implying an exergonic reaction. Based on this, it can be expected that DPPH radical in methanol will probably be scavenged following the SPLET mechanistic pathway. This hypothesis should be proven or annulled with the appropriate values of activation energies.

The obtained experimental and theoretical results (Table 2 and Table 5) suggest that **3d** is more reactive than **3e** against the DPPH radical. At the same time, IC_50_ values for **3a**, **3b**, and **3c** suggest that their activity is almost non-existent, while **∆*H*_PA_** values from Table 5 do not infer a significant difference between these three compounds and compound **3e**. This can be explained by the fact that compound **3e**, in comparison to compounds **3a**, **3b**, and **3c**, has one OH group more, which allows a better chance of reacting with the free radical species. The results presented in Table 5 suggest that the mechanism of radical scavenging against DPPH radical is SPLET, following the experimental results.

### 3.7. Potential Toxicology

To find whether compounds were suitable to be used as antioxidants in food industry, potential toxicity was investigated. For this purpose, the Prediction of Toxicity of Chemicals (ProTox-II) webserver protocol was used [48]. The results of the potential toxicity are presented in Table 6. To closely determine overall toxicity, investigated compounds are divided into six toxicity classes by the median lethal dose (LD_50_). These toxicity classes are defined according to the globally harmonized system of classification and labelling of chemicals (GHS). Compounds from Class I are highly toxic and fatal if swallowed (LD_50_ < 5mg/kg). The Class II compounds’ LD_50_ is found in the range between 5 and 50 mg/kg, and these compounds are still considered fatal if swallowed. Class III compounds are considered toxic, but with no fatal consequences, with LD_50_ in between 50 mg/kg and 300 mg/kg. Class **IV** compounds are harmful if swallowed, with LD_50_ between 300 mg/kg and 2000 mg/kg. Class **V** consist of compounds that may be harmful if swallowed (2000 mg/kg < LD_50_ ≤ 5000 mg/kg), while Class **VI** contains compounds that are considered non-toxic, with LD_50_ > 5000 mg/kg.

According to the results presented in Table 6, compounds **3a**, **3d**, and **3e** show significantly lower potential toxicity than esculetin and quercetin, which are natural products found in plants with a wide range of commercial uses [49,50,51]. Additionally, compounds with the OH group in C2″ position, i.e., **3a** and **3d**, showed a significant decrease in potential toxicity in comparison with parent molecule, 4-hydroxy coumarin. Bearing in mind that **3d** has shown excellent antioxidative properties, it is possible to consider it a potential supplement in the food industry, although experimental in vitro/in vivo studies are necessary for the definite conclusion.

## 4. Conclusions

Green synthesis of coumarin-hydroxybenzohydrazide derivatives by using a mixture of vinegar and ethanol as a catalyst and solvent is reported in this paper. This protocol provides isolation of pure products in moderate yield without any purification. The obtained compounds were structurally characterized by IR and NMR spectroscopy, and examined for their in vitro antioxidant activity. The results of the in vitro DPPH examination showed that two out of five of the investigated compounds, **3d** and **3e**, expressed high antioxidant activity. It should be mentioned that especially compound **3d**, with an activity slightly lower than the reference compounds NDGA and quercetin, acts as an excellent radical scavenger. In addition, the preferred radical scavenging pathways were theoretically investigated by the density functional theory approach. For that purpose, the values of various thermodynamic parameters, such as bond dissociation enthalpy, proton affinity, frontier molecular orbitals, HOMO-LUMO gap, and ionization potential were calculated. In silico calculations showed that HAT and SPLET reaction mechanisms are probable in non-polar and polar solvents, respectively. On the other hand, it was found that the SET-PT was not an operative mechanism in both solvents.

## Figures and Tables

**Table 1:** Isolated yield and HPLC purity of synthesized coumarin-hydroxybenzohydrazide derivatives **3**.

Entry	Compound	Isolated Yield (%)	HPLC Purity (%)
1	3a	65.31	91.44
2	3b	63.51	97.79
3	3c	66.83	98.50
4	3d	62.85	99.10
5	3e	65.71	97.46

**Table 2:** In vitro DPPH antioxidant activity of products **3** and reference compounds (%, IC_50_ and SF values).

Compound	DPPH Scavenging Ability (%)									IC_50_ (µM)	
	100 µM		50 µM		25 µM		* 150 µM	* 10 µM	* 2.5 µM		SF
	20 Min	60 Min	20 Min	60 Min	20 Min	60 Min	20 Min	20 Min	20 Min		
**3a**	2.9 ± 0.7	8.7 ± 0.7	0.6 ± 0.8	1.2 ± 0.7	0.6 ± 0.8	1.2 ± 0.7	nd	nd	nd	nd	nd
**3b**	4.5 ± 1.8	15.8 ± 0.2	0.1 ± 1.0	4.7 ± 1.7	0.1 ± 1.0	4.7 ± 1.7	nd	nd	nd	nd	nd
**3c**	31.5 ± 0.4	41.5 ± 0.6	20.4 ± 1.2	26.7 ± 0.9	20.4 ± 1.2	26.7 ± 0.9	56.4 ± 1.0	nd	nd	142.2 ± 2.7	nd
**3d**	94.9 ± 0.6	94.3 ± 0.9	92.9 ± 0.1	92.5 ± 0.4	92.9 ± 0.1	92.5 ± 0.4	nd	nd	42.9 ± 1.3	2.9 ± 0.1	4.4
**3e**	66.2 ± 1.8	75.3 ± 0.4	63.1 ± 1.1	73.6 ± 0.9	63.1 ± 1.1	73.6 ± 0.9	nd	47.7 ± 0.3	32.5 ± 0.7	12.9 ± 0.4	1.0
**NDGA**	94.5 ± 0.2	94.1 ± 0.7	94.6 ± 0.7	94.6 ± 0.6	94.6 ± 0.7	94.6 ± 0.6	nd	nd	59.9 ± 0.1	1.7 ± 0.1	7.4
**Quercetin**	95.1 ± 0.9	95.4 ± 0.8	95.3 ± 0.8	95.1 ± 0.9	95.3 ± 0.8	95.1 ± 0.9	nd	nd	60.9 ± 7.2	1.9 ± 0.1	6.6

* Relevant concentrations used in DPPH assay for determination of IC_50_ values; nd—Not determined.

**Table 3:** Thermodynamic parameters of antioxidant mechanism for investigated coumarin-hydroxybenzohydrazide derivatives in kJ moL^−1^.

	BENZENE					METHANOL				
Position	*HAT*	*SET-PT*		*SPLET*		*HAT*	*SET-PT*		*SPLET*	
	BDE	IP	PDE	PA	ETE	BDE	IP	PDE	PA	ETE
**3a**										
C2″-OH	404	714	123	446	391	373	552	19	191	380
C7″-NH	383		102	413	402	362		7	161	398
**3b**										
C4″-OH	382	688	127	407	408	381	551	28	185	394
C7″-NH	348		94	372	409	355		3	156	398
**3c**										
C4″-OH	377	693	118	422	388	377	528	47	200	375
C7″-NH	348		88	376	405	355		25	158	395
**3d**										
C2″-OH	372	706	99	420	385	362	519	41	191	369
C3″-OH	374		101	430	376	365		44	200	363
C7″-NH	348		75	343	438	355		34	133	420
**3e**										
C3″-OH	342	703	72	393	382	346	535	9	178	367
C4″-OH	344		74	380	397	348		11	168	378
C7″-NH	346		77	370	410	355		19	154	399

**Table 4:** The frontier orbital energy and energy gap (eV) of the investigated compounds.

Ligand	METHANOL			BENZENE		
	HOMO	LUMO	HL Gap (eV)	HOMO	LUMO	HL Gap (eV)
**3a**	−8.09	−1.41	6.68	−8.10	−1.37	6.72
**3b**	−8.09	−1.36	6.74	−8.01	−1.26	6.75
**3c**	−7.80	−1.35	6.45	−7.85	−1.26	6.59
**3d**	−7.81	−1.42	6.39	−7.87	−1.41	6.46
**3e**	−7.86	−1.36	6.50	−7.94	−1.28	6.66

**Table 5:** Calculated parameters of radical scavenging activity for coumarin hybrids in kJ moL^−1^.

METHANOL	*HAT*	*SET-PT*		*SPLET*	
	∆*H*_BDE_	∆*H*_IP_	∆*H*_PDE_	∆*H*_PA_	∆*H*_ETE_
**3a**					
C2″-OH	47	201	−142	50	7
C7″-NH	27		−163	2	34
**3b**					
C4″-OH	59	184	−134	23	28
C7″-NH	38		−159	−6	29
**3c**					
C4″-OH	50	161	−115	38	3
C7″-NH	24		−137	−4	25
**3d**					
C3″-OH	34	152	−118	38	−4
C2″-OH	31		−121	29	0.1
C7″-NH	24		−128	−29	53
**3e**					
C4″-OH	17	168	−151	6	9
C3″-OH	15		−152	16	−1.8
C7″-NH	25		−143	−8	32

**Table 6:** Predicted toxicity of the investigated compounds **3a**–**e** with warfarin, esculetin, 4-hydroxy coumarin, quercetin, and ethanol as referent compounds.

Compound:	Predicted Toxicity Class	Predicted LD_50_ (mg/kg)
**3a**	Class V	3000
**3b**	Class IV	1460
**3c**	Class IV	721
**3d**	Class V	3000
**3e**	Class IV	2000
**Warfarin**	Class I	2
**Esculetin**	Class IV	945
**4-hydroxy coumarin**	Class IV	2000
**Quercetin**	Class III	159
**Ethanol**	Class V	3450

## Data Availability

Data is contained within the article and supplementary material.

## Supplementary Materials

The following are available online at https://www.mdpi.com/article/10.3390/antiox10071106/s1.
