# Supplementary material for: Green One-Pot Synthesis of Coumarin-Hydroxybenzohydrazide Hybrids and Their Antioxidant Potency
Source: Antioxidants (Basel). 2021 Jul 10;10(7):1106. doi: 10.3390/antiox10071106 (PMC8301024; doi:10.3390/antiox10071106)
Supplement: Supplementary file 1 [file antioxidants-10-01106-s001.zip › antioxidants-1271141-supplementary.pdf]

Supplementary material

Green one-pot synthesis of coumarin-hydroxybenzohydrazide hybrids and their antioxidant potency

Marko R. Antonijević[1,2] , Dušica M. Simijonović[1], Edina H. Avdović[1], Andrija Ćirić[2], Zorica D. Petrović[2], Jasmina Dimitrić Marković[3], Višnja Stepanić[4], Zoran S. Marković[1*]

[1]University of Kragujevac, Institute for Information Technologies, Department of Science, Jovana Cvijića bb, 34000 Kragujevac, Serbia
[2]University of Kragujevac, Faculty of Science, Department of Chemistry, Radoja Domanovića 12, 34000 Kragujevac, Serbia
[3]University of Belgrade, Faculty of Physical Chemistry, Studentski trg 12-16, 11000 Belgrade, Serbia
[4]Ruđer Bošković Institute, Bijenička cesta 54, 10000 Zagreb, Croatia
*Correspondace: e-mail adress: zmarkovic@uni.kg.ac.rs ; edina.avdovic@pmf.kg.ac.rs; phone number: +38134/610-01-95

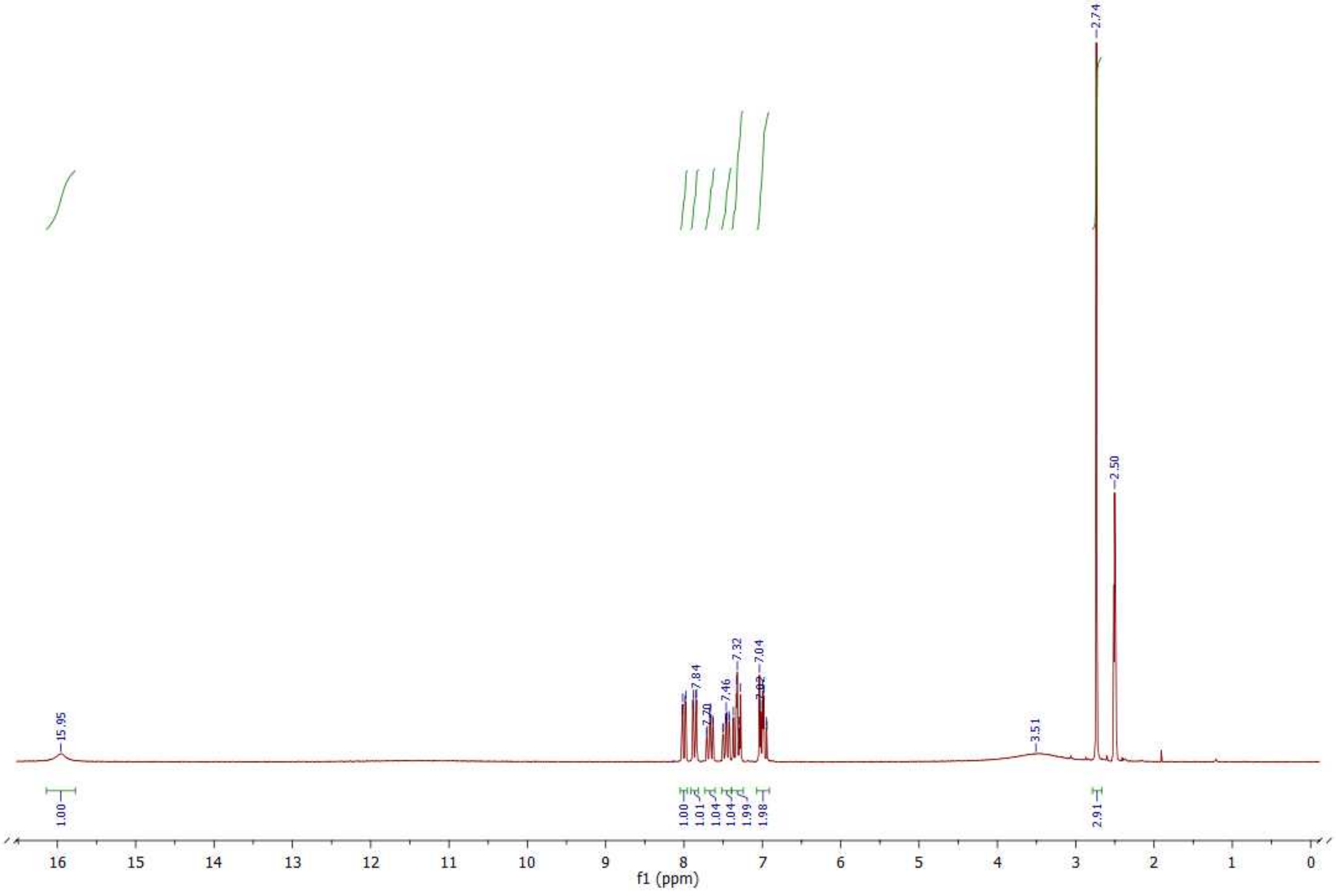

Figure S1: ^{1}H NMR spectrum of *(E)-N'-(1-(2,4-dioxochroman-3-ylidene)ethyl)-2-hydroxybenzohydrazide (**3a**)*

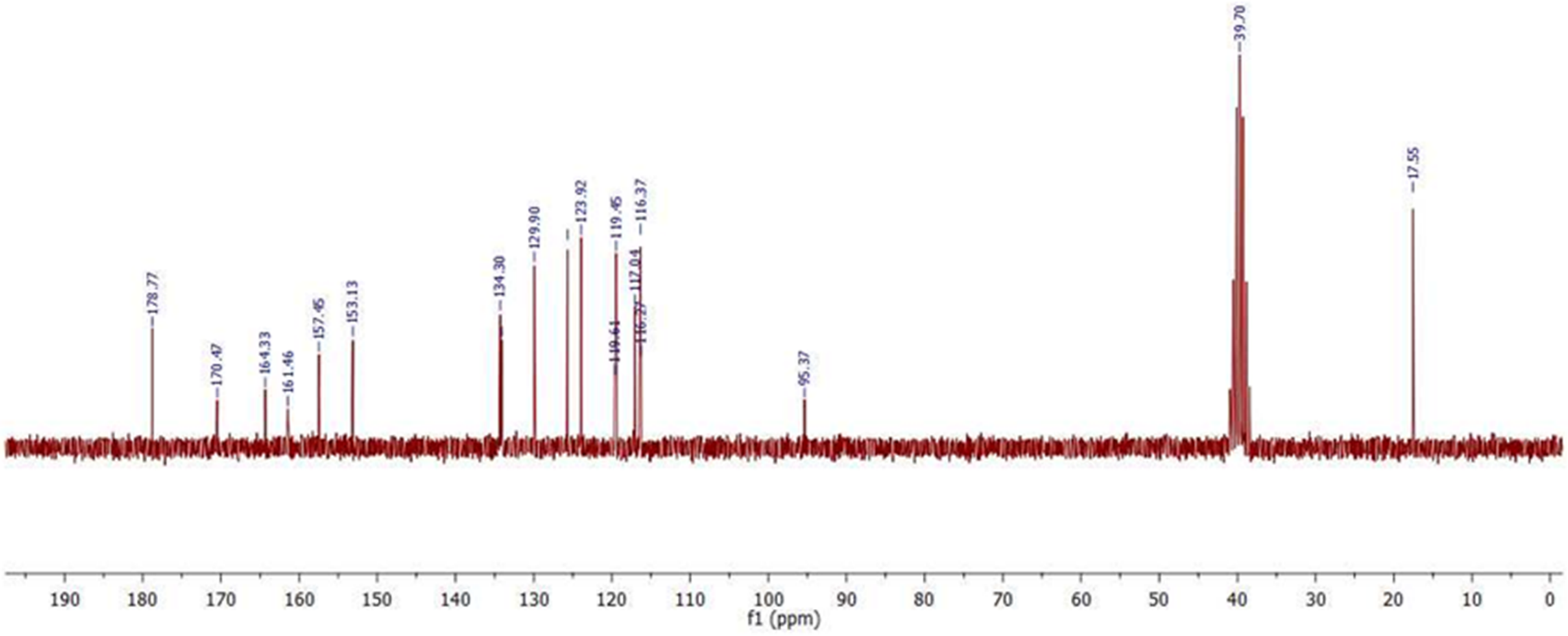

Figure S2: ^{13}C NMR spectrum of *(E)-N'-(1-(2,4-dioxochroman-3-ylidene)ethyl)-2-hydroxybenzohydrazide (**3a**)*

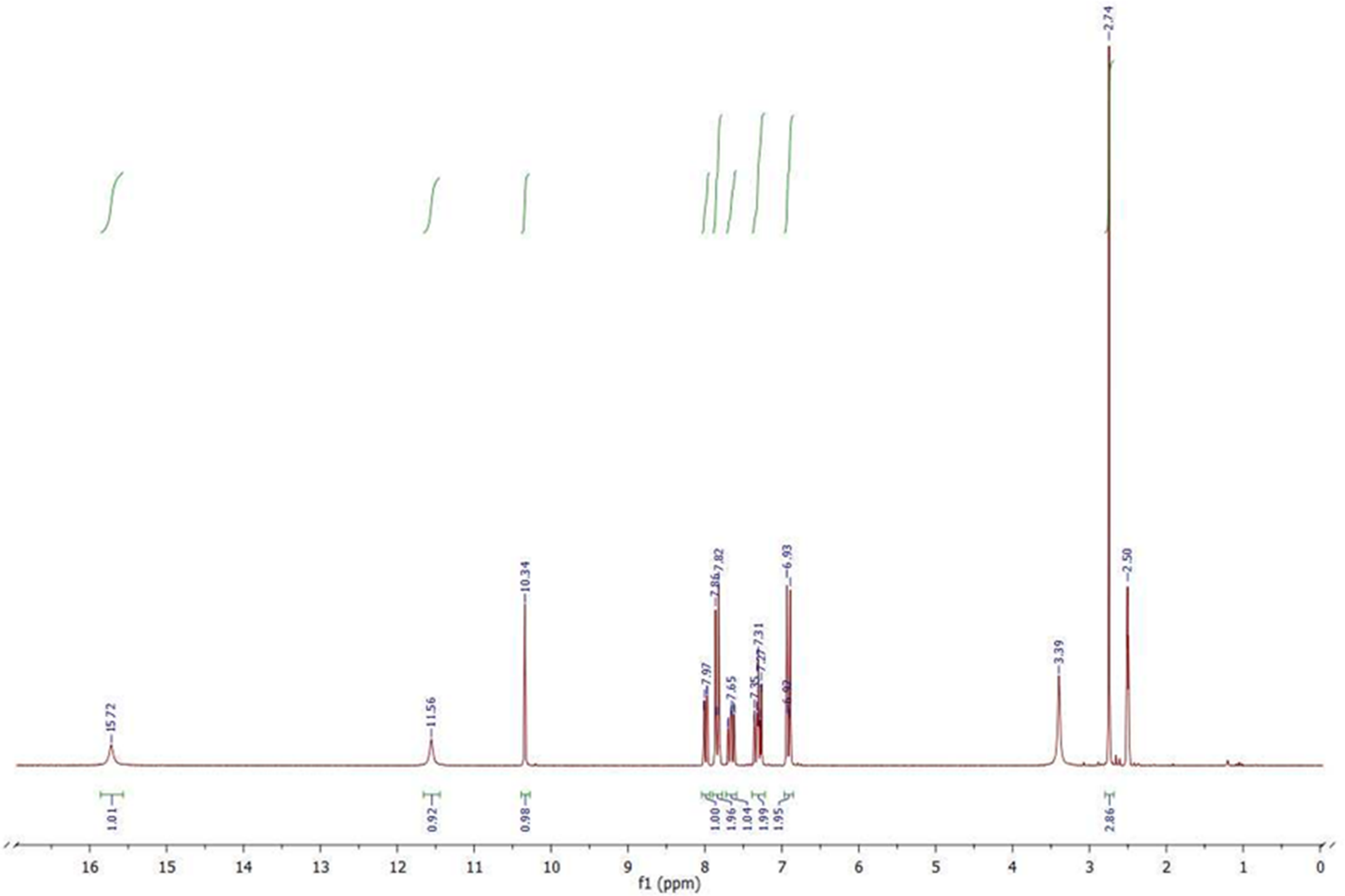

Figure S3: ^{1}H NMR spectrum of *(E)-N'-(1-(2,4-dioxochroman-3-ylidene)ethyl)-4-hydroxybenzohydrazide* (***3b***)

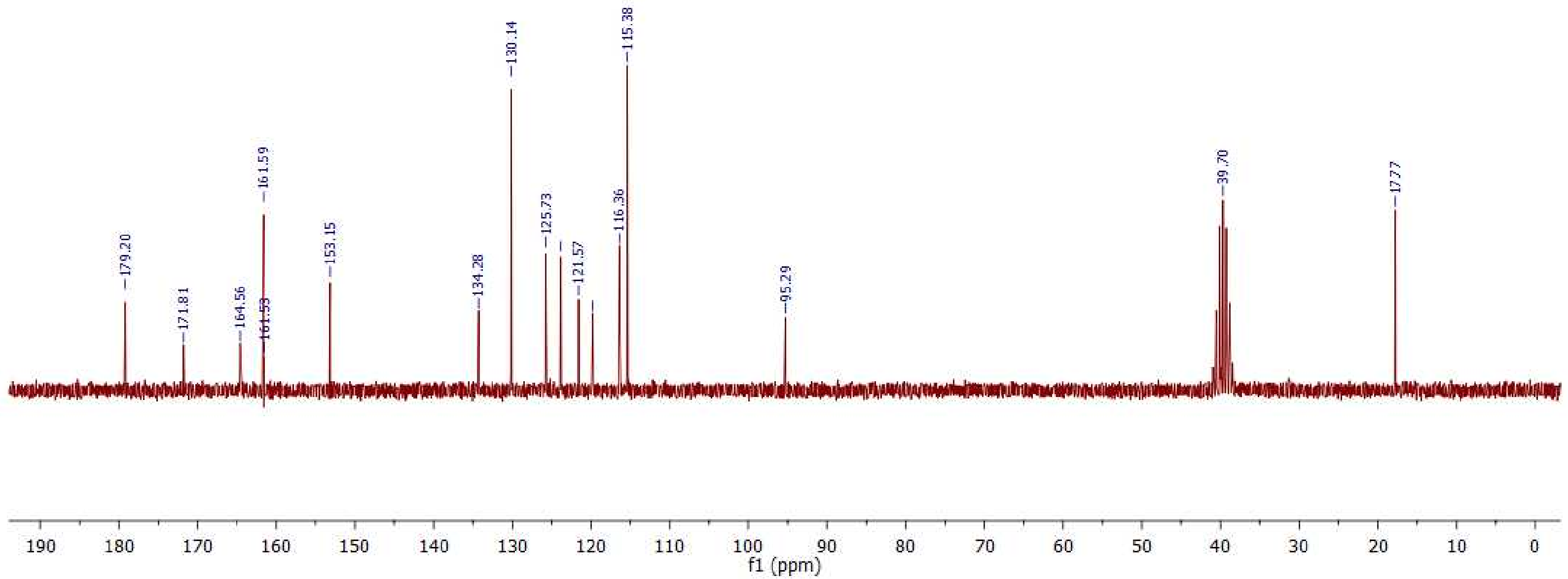

Figure S4: ^{13}C NMR spectrum of *(E)-N'-(1-(2,4-dioxochroman-3-ylidene)ethyl)-4-hydroxybenzohydrazide* ***(3b)***

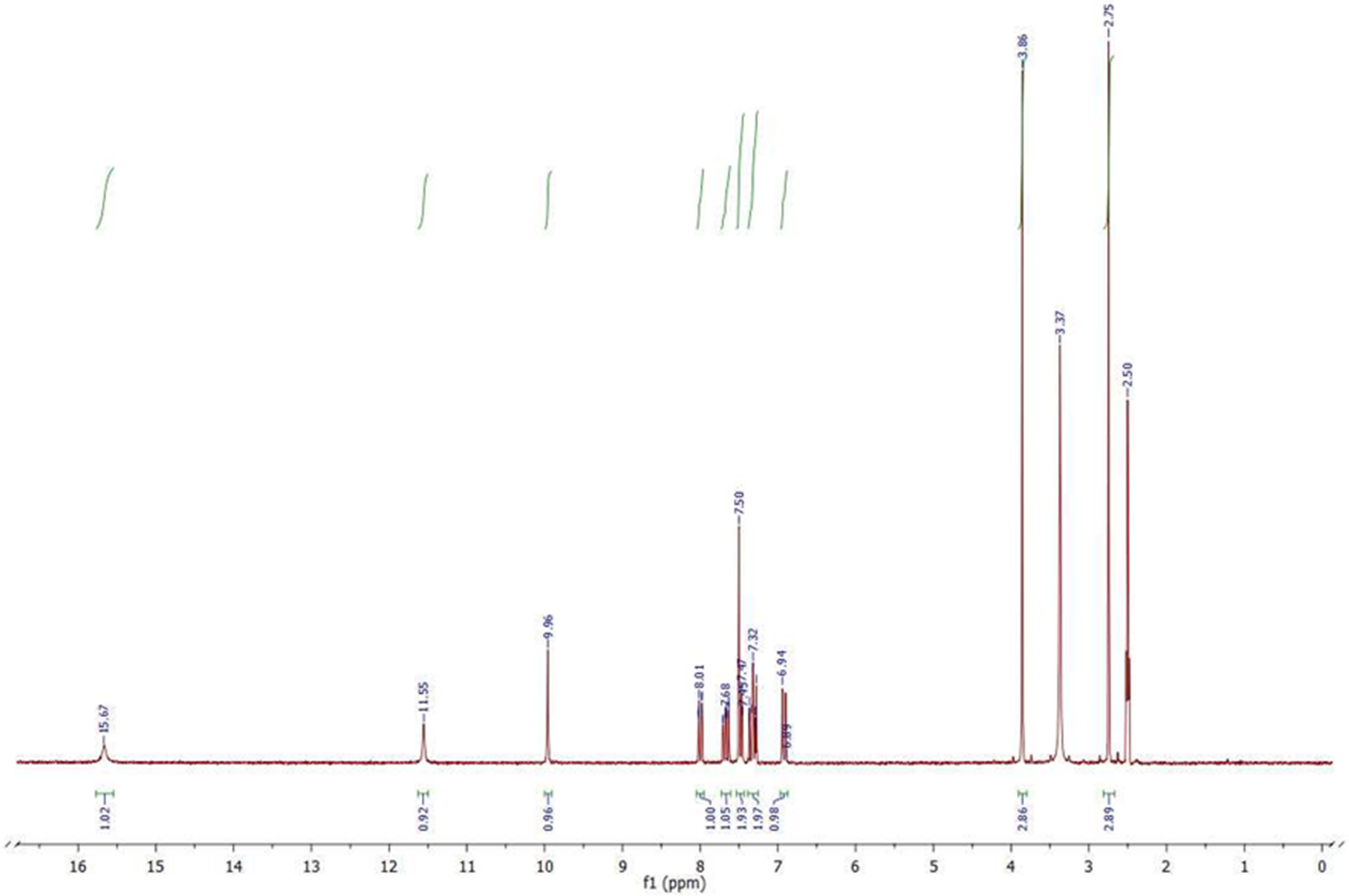

Figure S5: ^{1}H NMR spectrum *(E)-N'-(1-(2,4-dioxochroman-3-ylidene)ethyl)-4-hydroxy-3-methoxybenzohydrazide* ***(3c)***

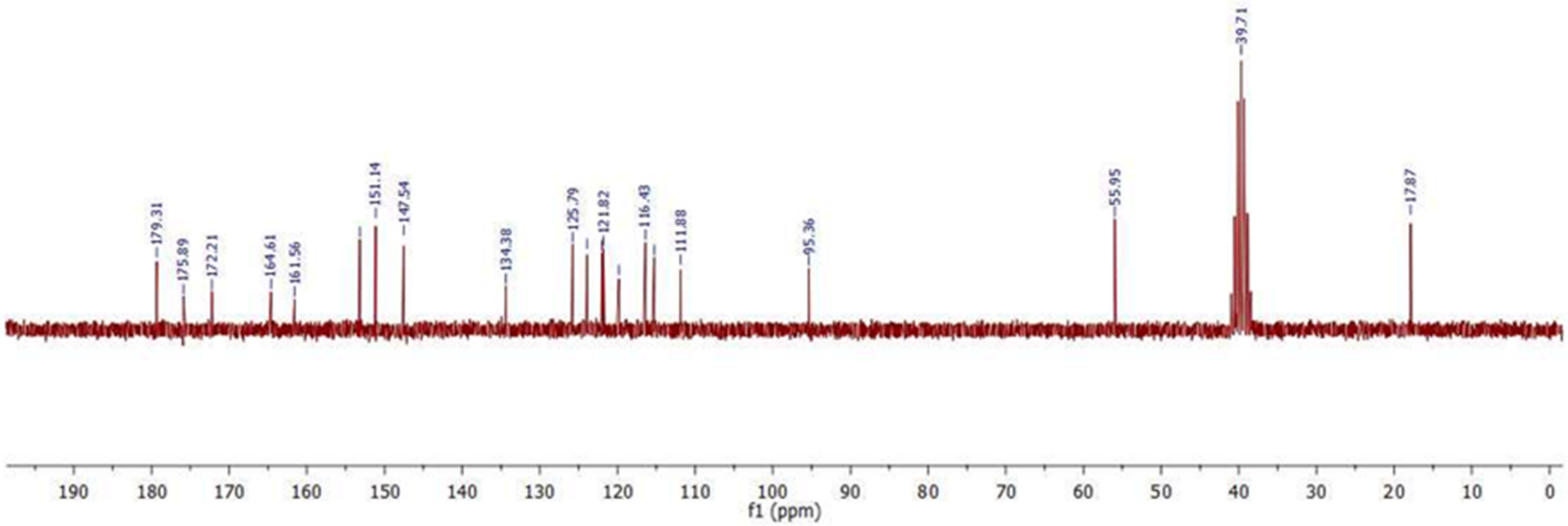

Figure S6: ^{13}C NMR spectrum of *(E)-N'-(1-(2,4-dioxochroman-3-ylidene)ethyl)-4-hydroxy-3-methoxybenzohydrazide (**3c**)*

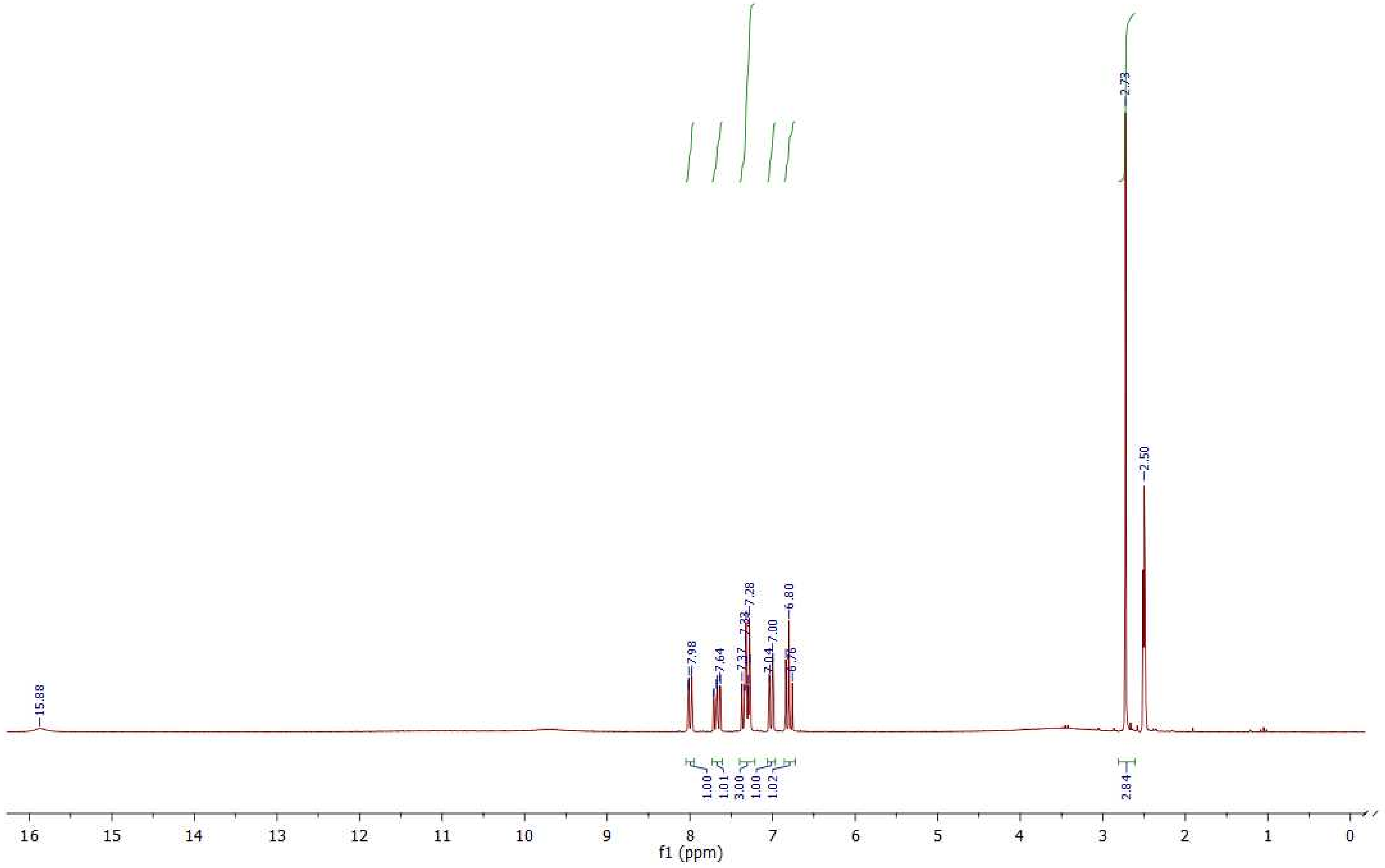

Figure S7: ^{1}H NMR spectrum *(E)-N'-(1-(2,4-dioxochroman-3-ylidene)ethyl)-2,3-dihydroxybenzohydrazide (**3d**)*

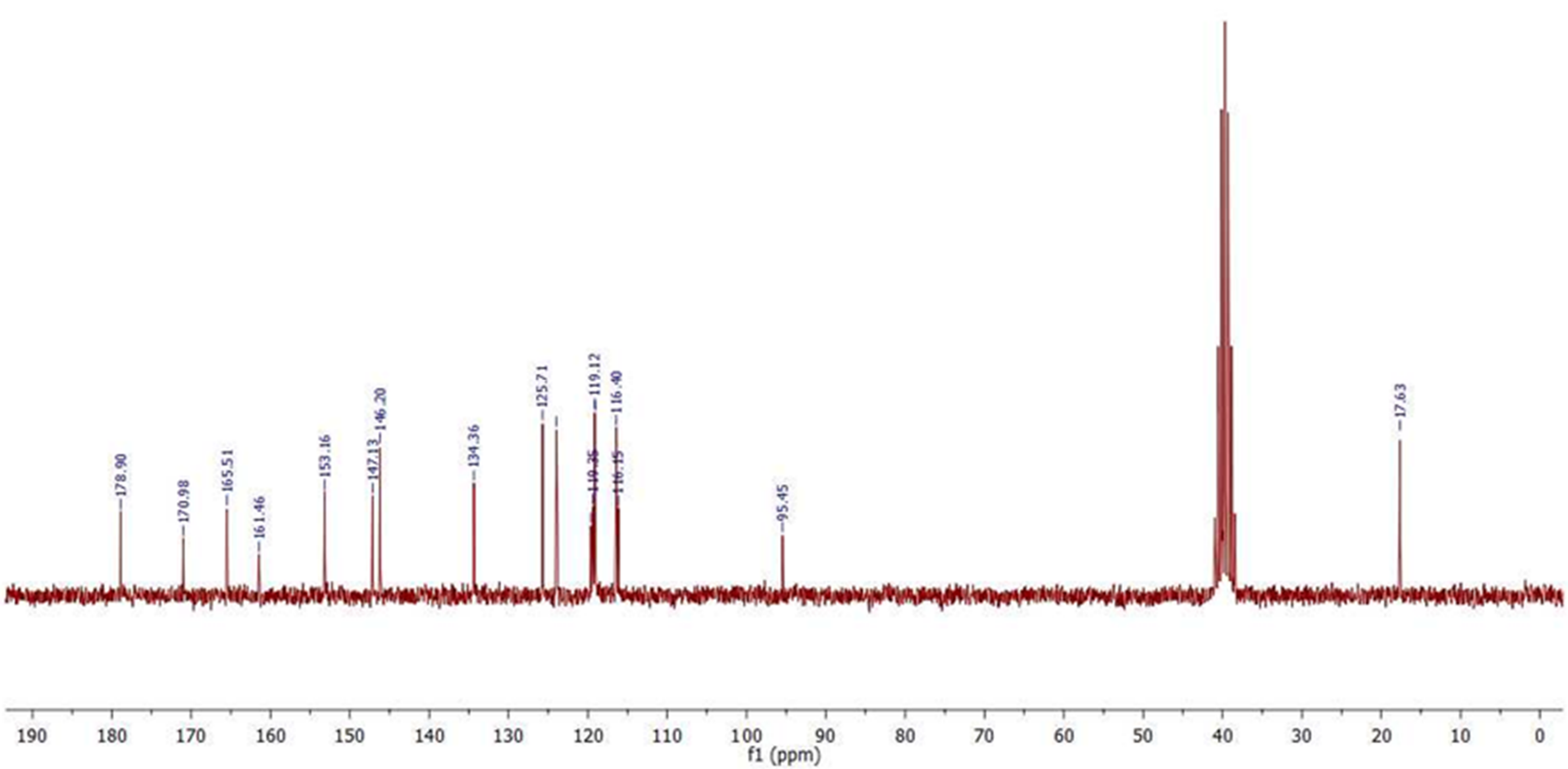

Figure S8: ^{13}C NMR spectrum of *(E)-N'-(1-(2,4-dioxochroman-3-ylidene)ethyl)-2,3-dihydroxybenzohydrazide (**3d**)*

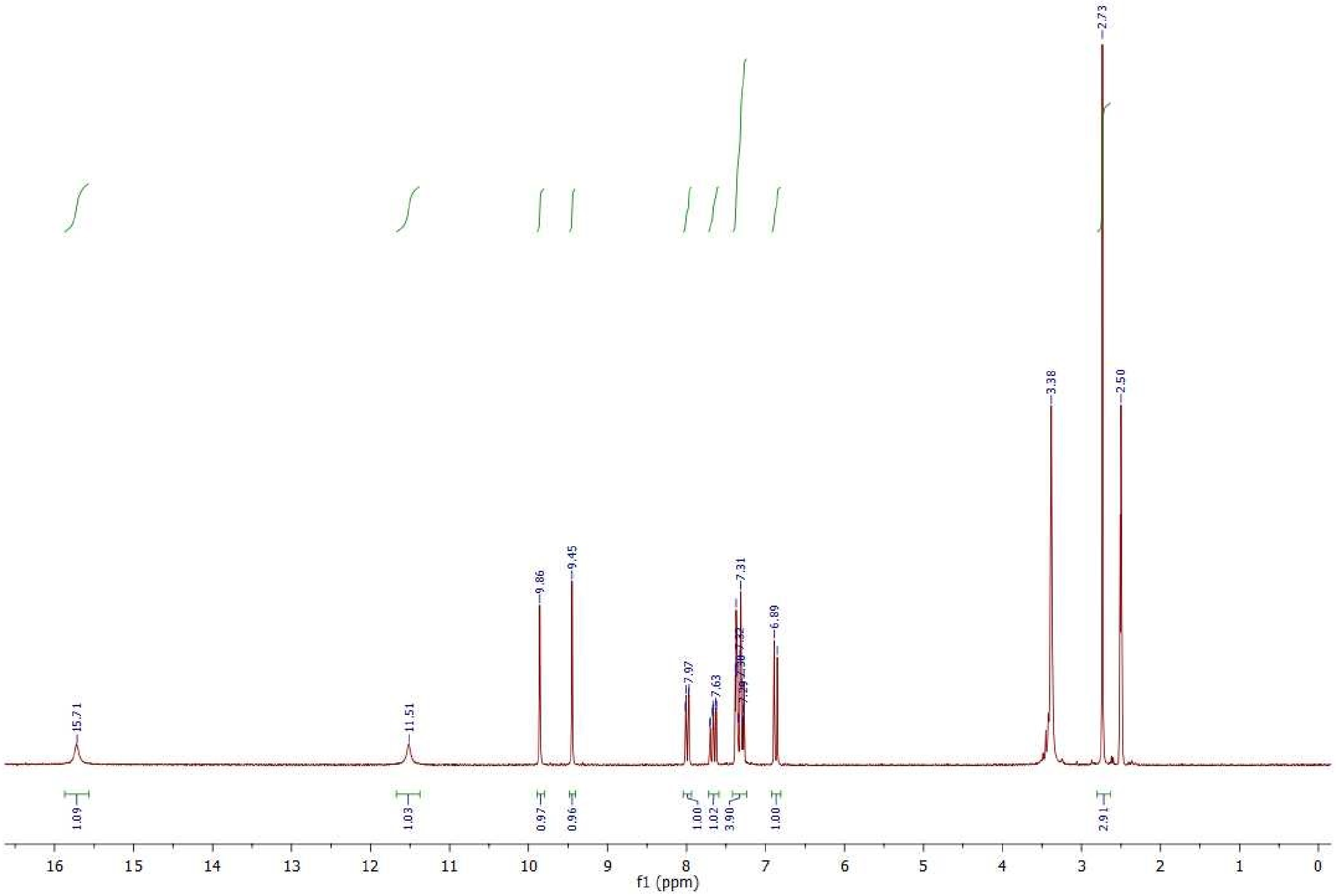

Figure S9: ^{1}H NMR spectrum *(E)-N'-(1-(2,4-dioxochroman-3-ylidene)ethyl)-3,4-dihydroxybenzohydrazide* (***3e***)

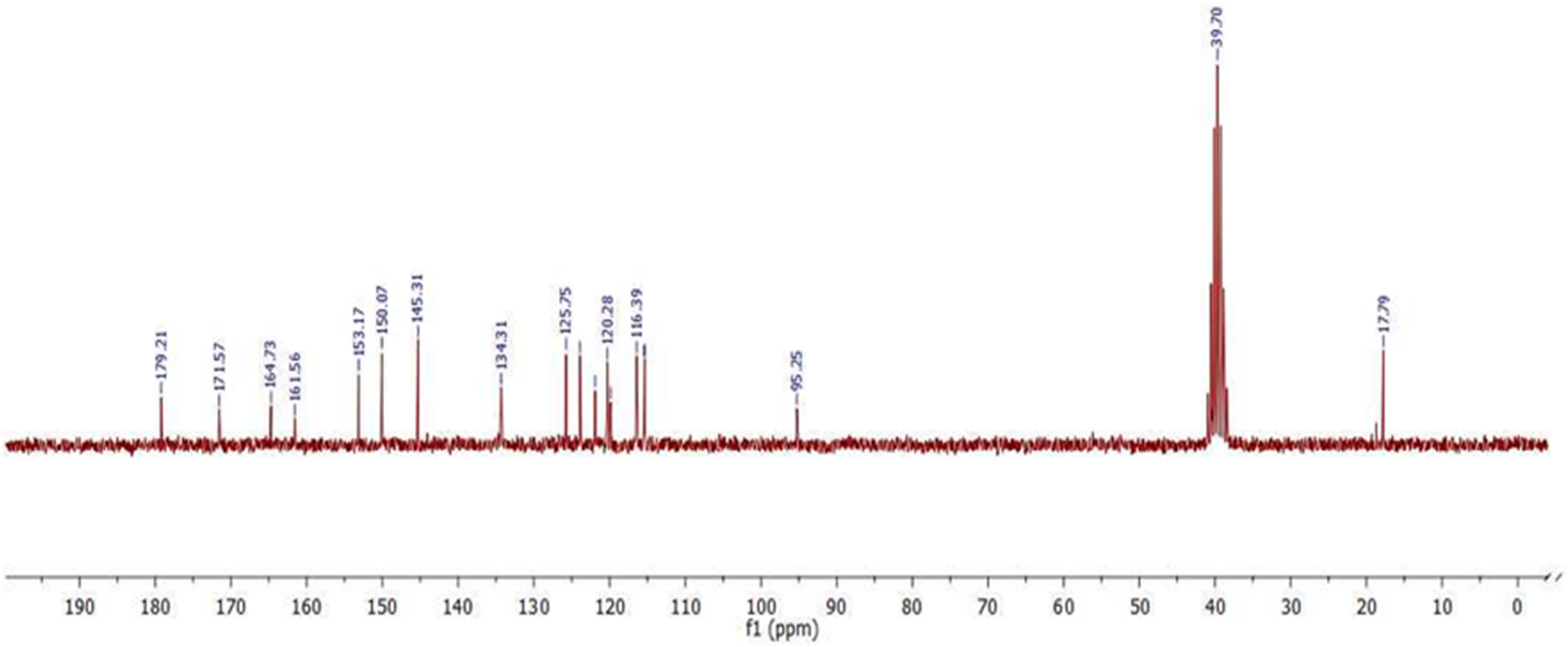

Figure S10: ^{13}C NMR spectrum of *(E)-N'-(1-(2,4-dioxochroman-3-ylidene)ethyl)-3,4-dihydroxybenzohydrazide* ***(3e)***

HPLC analysis of investigated coumarin-hydoxybenzohydrazide 3

Table S1: HPLC data for compound **3a**

	Retention time (min)	Area	Peak height	%
1	2.404	48134	5402	0.2689
2	2.559	167142	8624	0.9337
3	16.227	1867	250	0.0104
4	16.947	3556	276	0.0199
5	20.509	2023	222	0.0113
6	20.809	113605	8407	0.6347
7	22.639	1001	93	0.0056
8	23.321	45170	1544	0.2523
9	24.816	17035	1727	0.0952
10	25.168	5285	583	0.0295
11	**25.6**	**16368320**	**1016256**	**91.4426**
12	31.701	1126958	16032	6.2958
Wavelenght: 353 nm; Duration: 45 min; Volume: 20.00 µl				

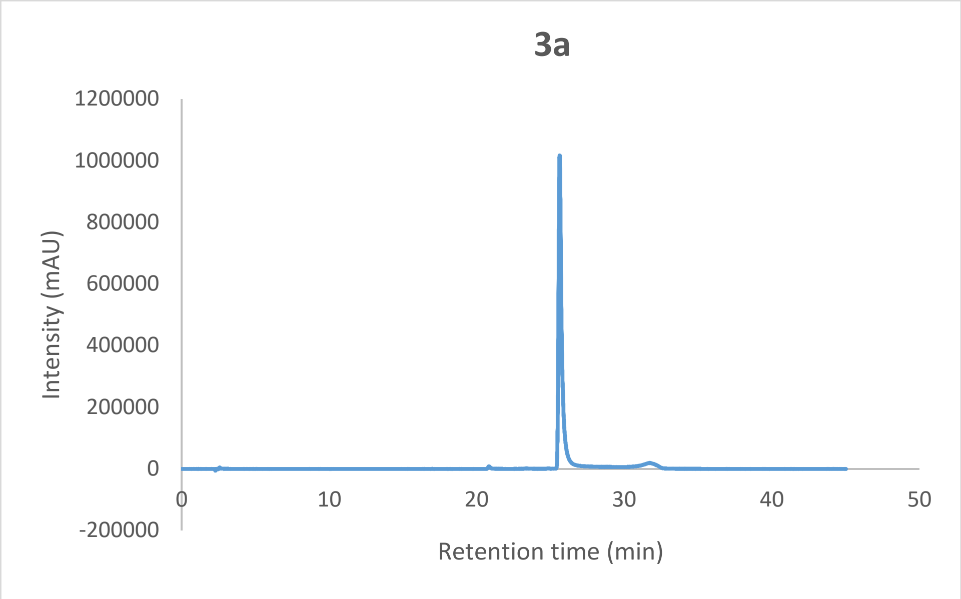

Figure S11: HPLC chromatogram of compound **3a**

Table S2: HPLC data for compound **3b**

	Retention time (min)	Area	Peak height	%
1	16.232	4726	661	0.0363
2	16.919	1975	249	0.0152
3	17.05	1776	270	0.0136
4	19.159	1452	181	0.0111
5	20.839	15522	1276	0.1191
6	**22.255**	**12749399**	**1343934**	**97.7935**
7	23.69	1804	240	0.0138
8	24.427	1016	71	0.0078
9	24.884	163054	12910	1.2507
10	25.693	90253	2590	0.6923
11	32.35	5009	512	0.0384
12	36.585	1073	112	0.0082
Wavelenght: 337 nm; Duration: 45 min; Volume: 20.00 µl				

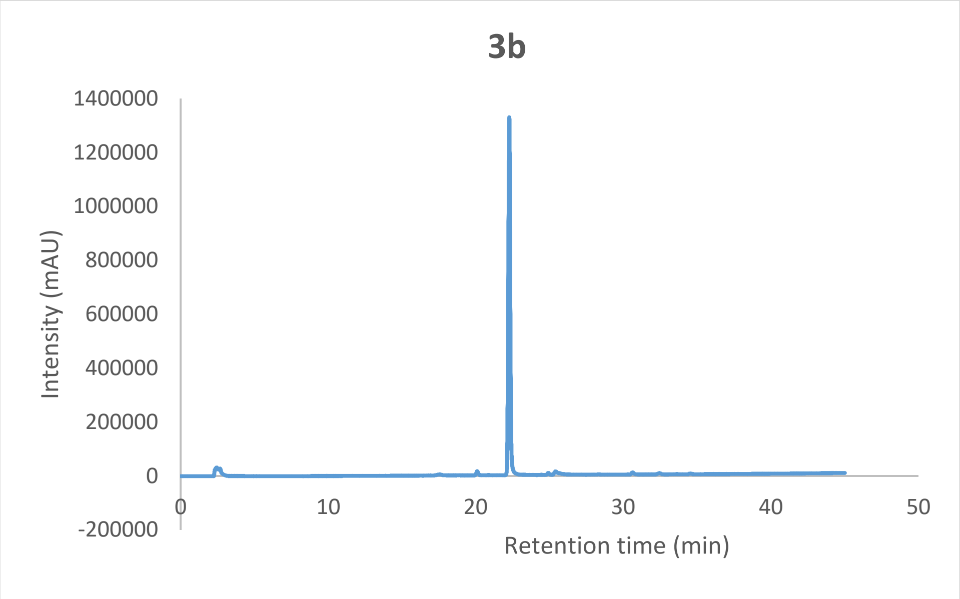

Figure S12: HPLC chromatogram of compound **3b**

Table S3: HPLC data for compound **3c**

	Retention time (min)	Area	Peak height	%
1	16.204	2244	324	0.011
2	16.891	2266	266	0.0111
3	17.661	5802	610	0.0283
4	19.455	6649	728	0.0325
5	20.841	20743	2173	0.1013
6	21.26	4303	492	0.021
7	22.251	34502	3415	0.1685
8	**22.734**	**20163780**	**1957944**	**98.4982**
9	24.918	214006	18546	1.0454
10	25.723	5322	365	0.026
11	26.014	5283	427	0.0258
12	32.382	4793	491	0.0234
Wavelenght: 336 nm; Duration: 45 min; Volume: 20.00 µl				

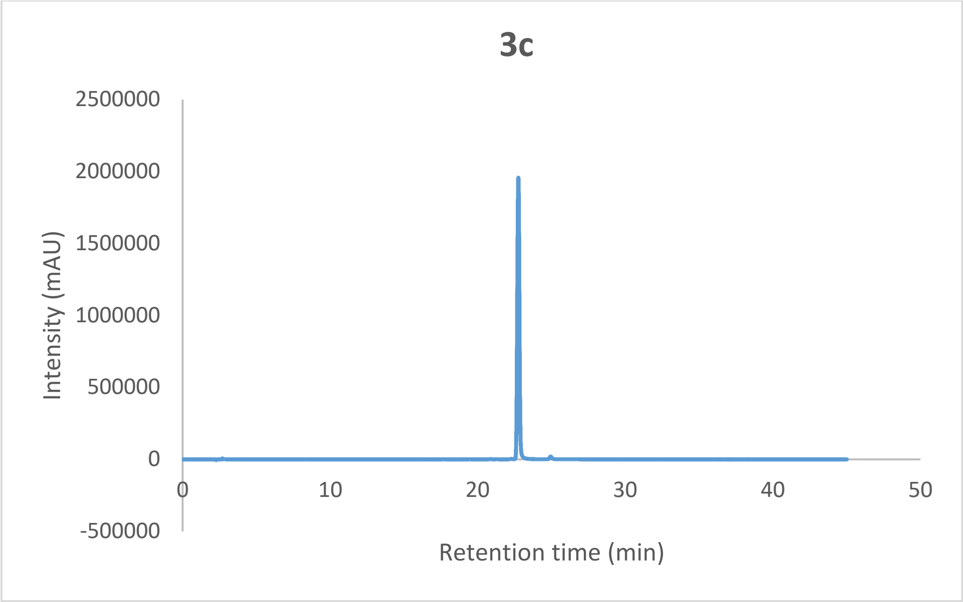

Figure S13: HPLC chromatogram of compound **3c**

Table S4: HPLC data for compound **3d**

	Retention time (min)	Area	Peak height	%
1	16.288	3120	405	0.0306
2	16.968	4680	382	0.0459
3	17.885	3520	153	0.0345
4	18.677	2592	176	0.0254
5	18.801	2029	235	0.0199
6	20.157	1581	144	0.0155
7	20.739	7554	404	0.0741
8	22.219	1350	117	0.0132
9	22.507	4962	414	0.0487
10	22.683	7715	660	0.0756
11	**23.403**	**10107439**	**597973**	**99.0957**
12	24.913	48187	3681	0.4724
13	32.362	4945	426	0.0485
Wavelenght: 345 nm; Duration: 45 min; Volume: 20.00 µl				

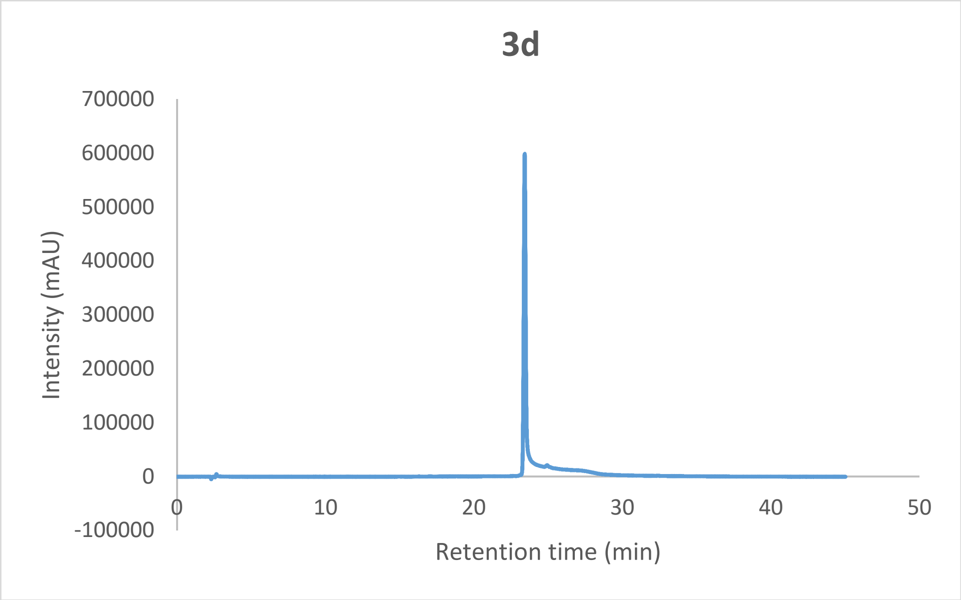

Figure S14: HPLC chromatogram of compound **3d**

Table S5: HPLC data for compound **3e**

	Retention time (min)	Area	Peak height	%
1	2.495	65821	4058	0.3415
2	2.705	113829	7695	0.5906
3	16.26	6105	772	0.0317
4	16.472	1785	162	0.0093
5	16.939	2513	230	0.013
6	17.085	2563	302	0.0133
7	17.964	3348	278	0.0174
8	18.32	1868	148	0.0097
9	18.635	3170	167	0.0164
10	18.97	2664	218	0.0138
11	19.535	4712	450	0.0245
12	20.082	2856	223	0.0148
13	**20.856**	**18783003**	**1658268**	**97.46**
14	22.487	4212	332	0.0219
15	23.136	5274	349	0.0274
16	23.74	47480	1463	0.2464
17	24.363	1448	337	0.0075
18	24.437	1884	339	0.0098
19	24.852	199452	14332	1.0349
20	25.429	8786	596	0.0456
21	25.771	1949	164	0.0101
22	26.234	1620	161	0.0084
23	32.338	4825	440	0.025
24	36.533	1355	117	0.007
Wavelenght: 336 nm; Duration: 45 min; Volume: 20.00 µl				

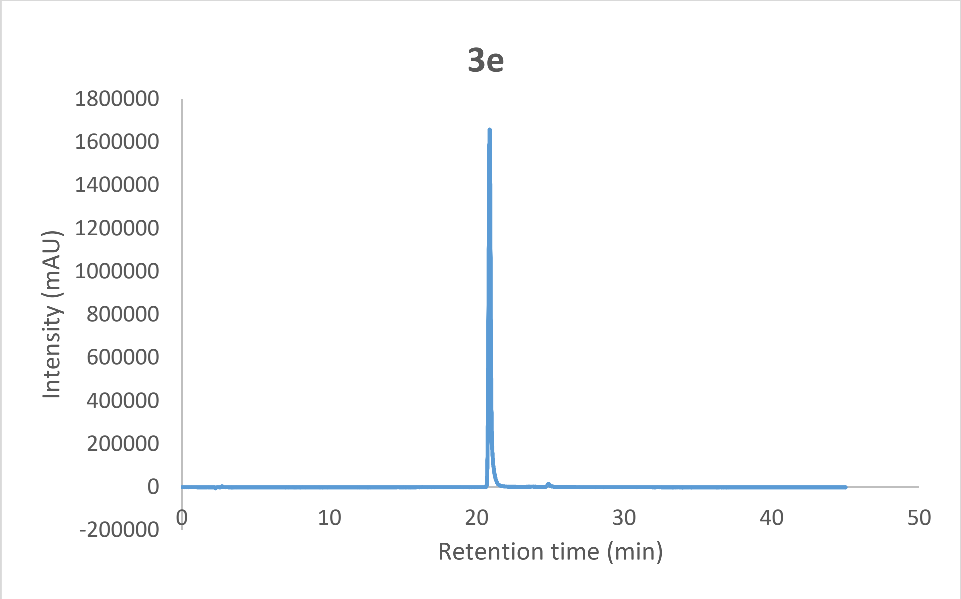

Figure S15: HPLC chromatogram of compound **3e**

Determination of IC_{50} value for compound 3d

Table S6: Determination of IC_{50} value for compound 3d

Concentration (µmol/L)	activity (%)		
	1st sample	2nd sample	3rd sample
2	31.97853	31.92145	31.44702
2.25	37.38497	35.30997	37.63768
2.5	42.17791	44.43589	42.08128
2.75	51.45706	51.21294	51.87998
3	52.22393	52.40662	53.09533
3.25	55.52147	54.37043	55.86783
3.5	59.77761	58.37505	58.48842
3.75	71.66411	70.65845	67.83137
4	74.84663	73.50789	76.37676
4.25	79.7546	80.51598	80.25066

All analyses for determination of IC_{50} values were run in triplicate and averaged.

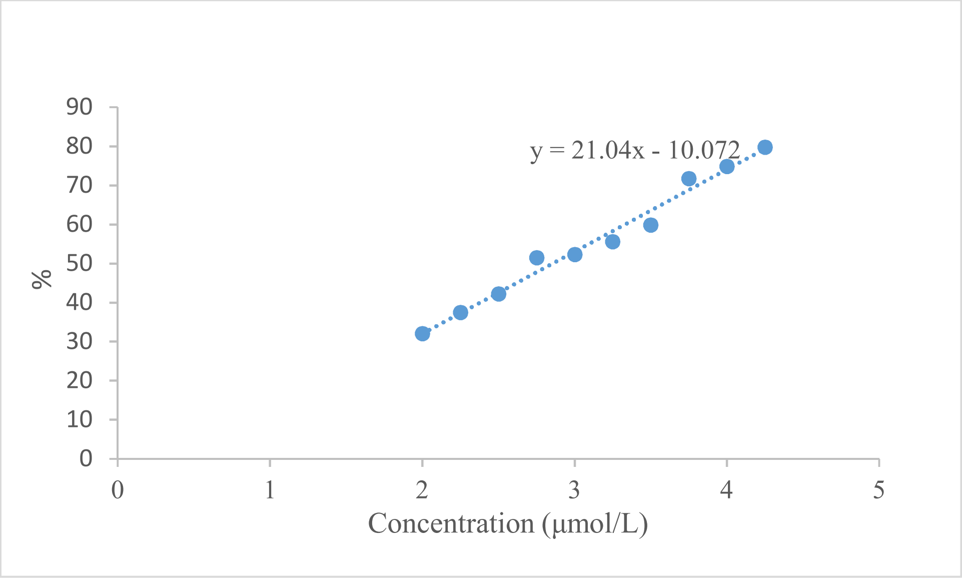

Graphic S1: Determination of IC_{50} value for compound **3d**-sample 1

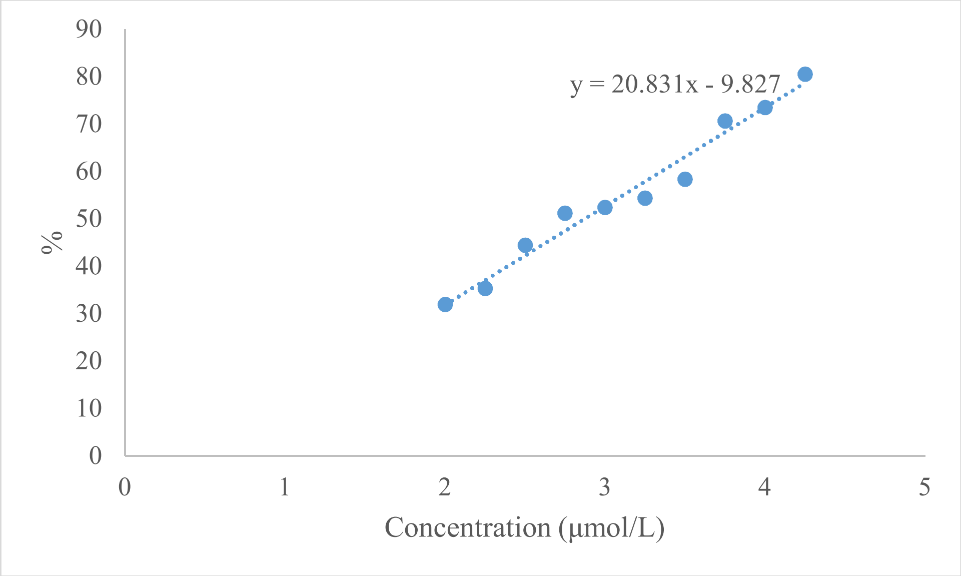

Graphic S2: Determination of IC_{50} value for compound **3d**-sample 2

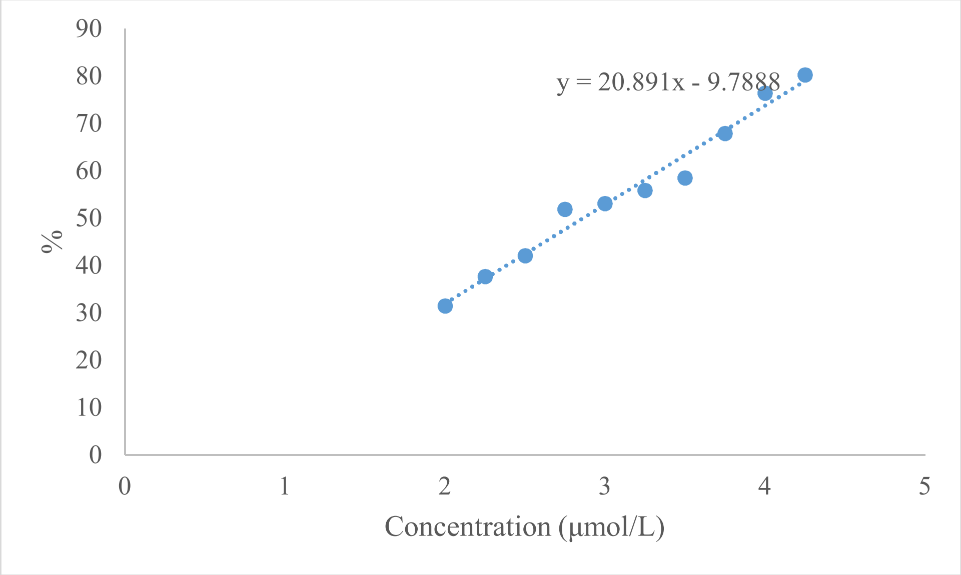

Graphic S3: Determination of IC_{50} value for compound **3d**-sample 3

The calculation of IC_{50} values from the equation of the line y=ax+b using the equation IC_{50}=(50-b)/a

Table S7: Determination of IC_{50} value for compound **3d**

	1st	2nd	3rd	AVERAGE(IC_{50})
a	21.04	20.831	20.891	
b	-10.072	-9.827	-9.7888	
IC_{50}	2.855133	2.872018	2.861941	2.86

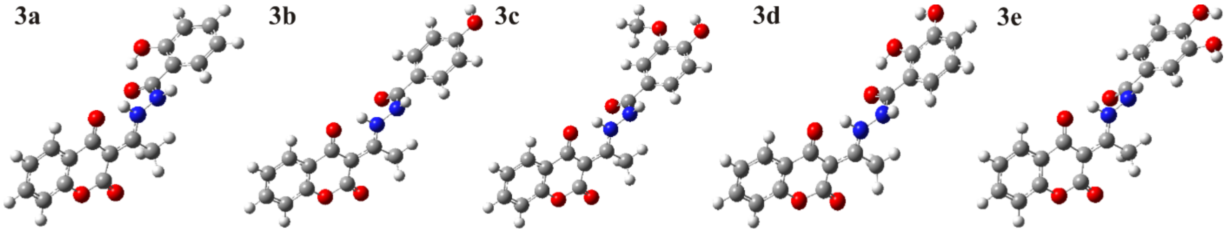

Figure S16: The most stable conformations of the investigated coumarin-hydroxybenzohydrazide derivatives

Equations for describing the mechanisms of radical scavenging activity of investigated compounds:

$$\text{Ar-OH} + \text{DPPH}^{\bullet} \rightarrow \text{Ar-O}^{\bullet} + \text{DPPH-H} \quad (1s)$$

$$\text{Ar-OH} + \text{DPPH}^{\bullet} \rightarrow \text{Ar-OH}^{\bullet+} + \text{DPPH}^{-} \quad (2s)$$

$$\text{Ar-OH}^{\bullet+} + \text{DPPH}^{-} \rightarrow \text{Ar-OH}^{\bullet} + \text{DPPH-H} \quad (3s)$$

$$\text{Ar-OH} + \text{DPPH}^{-} \rightarrow \text{Ar-O}^{-} + \text{DPPH-H} \quad (4s)$$

$$\text{Ar-O}^{-} + \text{DPPH}^{\bullet} \rightarrow \text{Ar-O}^{\bullet} + \text{DPPH}^{-} \quad (5s)$$

Thermodynamic parameters that describe radical scavenging activity of investigated compounds:

$$\Delta H_{\text{BDE}} = H(\text{Ar-O}^{\bullet}) + H(\text{DPPH-H}) - H(\text{Ar-OH}) - H(\text{DPPH}^{\bullet}) \quad (6s)$$

$$\Delta H_{\text{IP}} = H(\text{Ar-OH}^{\bullet+}) + H(\text{DPPH}^{-}) - H(\text{Ar-OH}) - H(\text{DPPH}^{\bullet}) \quad (7s)$$

$$\Delta H_{\text{PDE}} = H(\text{Ar-O}^{\bullet}) + H(\text{DPPH-H}) - H(\text{Ar-OH}^{\bullet+}) - H(\text{DPPH}^{-}) \quad (8s)$$

$$\Delta H_{\text{PA}} = H(\text{Ar-O}^{-}) + H(\text{DPPH-H}) - H(\text{Ar-OH}) - H(\text{DPPH}^{-}) \quad (9s)$$

$$\Delta H_{\text{ETE}} = H(\text{Ar-O}^{\bullet}) + H(\text{DPPH}^{-}) - H(\text{Ar-O}^{-}) - H(\text{DPPH}^{\bullet}) \quad (10s)$$

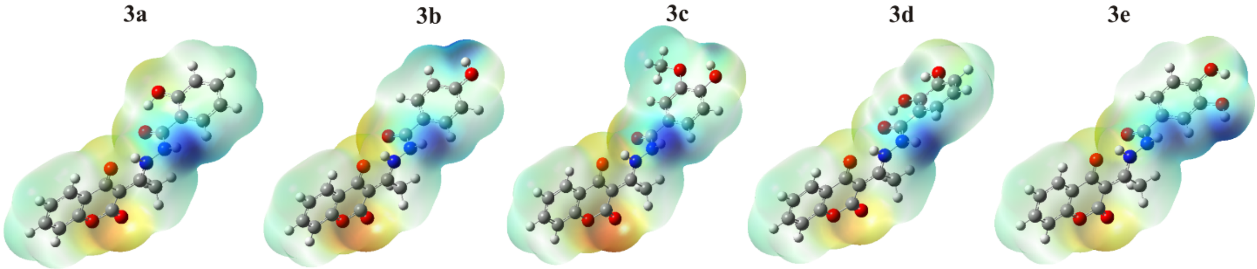

Figure S17. ESP maps of the investigated coumarin-hydroxybenzohydrazide derivatives where red collored area indicates negative charge distribution, green indicates neutral, and blue indicates positive charge

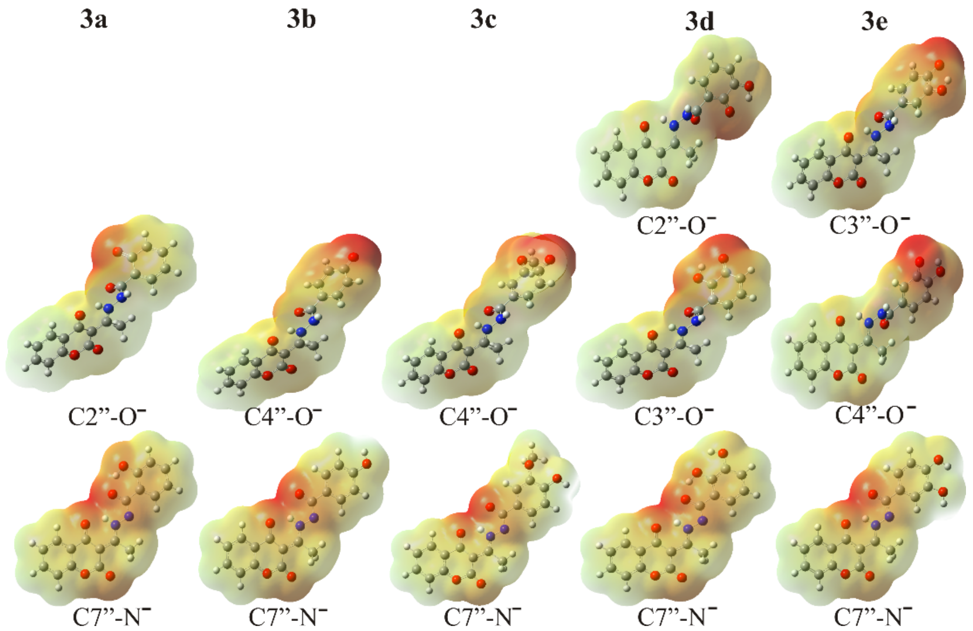

Figure S18: ESP maps of the anions of investigated compounds where red collored area indicates negative charge distribution, green/yellow indicates neutraly charged area

Table S8: Bond lenghts of the investigated coumarin-hydroxybenzohydrazide derivatives

3a		3b		3c		3d		3e	
**Bond distance (Å)**	**M062X**	**Bond distance (Å)**	**M062X**	**Bond distance (Å)**	**M062X**	**Bond distance (Å)**	**M062X**	**Bond distance (Å)**	**M062X**
O1-C2	1.376	O1-C2	1.376	O1-C2	1.376	O1-C2	1.375	O1-C2	1.376
C2-O2	1.208	C2-O2	1.208	C2-O2	1.208	C2-O2	1.208	C2-O2	1.208
C2-C3	1.456	C2-C3	1.456	C2-C3	1.452	C2-C3	1.457	C2-C3	1.455
C3-C4	1.450	C3-C4	1.449	C3-C4	1.448	C3-C4	1.450	C3-C4	1.448
C3-C1’	1.419	C3-C1’	1.422	C3-C1’	1.422	C3-C1’	1.419	C3-C1’	1.422
C4-O4	1.239	C4-O4	1.240	C4-O4	1.240	C4-O4	1.240	C4-O4	1.240
C4-C10	1.473	C4-C10	1.474	C4-C10	1.474	C4-C10	1.473	C4-C10	1.473
C10-C5	1.400	C10-C5	1.400	C10-C5	1.400	C10-C5	1.400	C10-C5	1.400
C10-C9	1.389	C10-C9	1.389	C10-C9	1.388	C10-C9	1.388	C10-C9	1.389
C5-C6	1.383	C5-C6	1.383	C5-C6	1.383	C5-C6	1.383	C5-C6	1.383
C5-H5	1.083	C5-H5	1.083	C5-H5	1.083	C5-H5	1.083	C5-H5	1.083
C6-C7	1.400	C6-C7	1.400	C6-C7	1.400	C6-C7	1.400	C6-C7	1.400
C6-H6	1.083	C6-H6	1.083	C6-H6	1.083	C6-H6	1.083	C6-H6	1.083
C7-C8	1.385	C7-C8	1.385	C7-C8	1.385	C7-C8	1.385	C7-C8	1.385
C7-H7	1.083	C7-H7	1.083	C7-H7	1.083	C7-H7	1.083	C7-H7	1.083
C8-C9	1.393	C8-C9	1.393	C8-C9	1.393	C8-C9	1.393	C8-C9	1.393
C8-H8	1.083	C8-H8	1.083	C8-H8	1.083	C8-H8	1.083	C8-H8	1.083
C9-O1	1.363	C9-O1	1.363	C9-O1	1.362	C9-O1	1.363	C9-O1	1.363
C1’-C2’	1.494	C1’-C2’	1.494	C1’-C2’	1.494	C1’-C2’	1.494	C1’-C2’	1.494
C2’-H2’(1)	1.091	C2’-H2’(1)	1.091	C2’-H2’(1)	1.091	C2’-H2’(1)	1.091	C2’-H2’(1)	1.091
C2’-H2’(2)	1.088	C2’-H2’(2)	1.087	C2’-H2’(2)	1.087	C2’-H2’(2)	1.087	C2’-H2’(2)	1.087
C2’-H2’(3)	1.086	C2’-H2’(3)	1.086	C2’-H2’(3)	1.086	C2’-H2’(3)	1.086	C2’-H2’(3)	1.086
C1’-N1’	1.330	C1’-N1’	1.327	C1’-N1’	1.327	C1’-N1’	1.330	C1’-N1’	1.327

N1’-H1’	1.029	N1’-H1’	1.028	N1’-H1’	1.028	N1’-H1’	1.029	N1’-H1’	1.029
N1’-N7”	1.376	N1’-N7”	1.376	N1’-N7”	1.376	N1’-N7”	1.375	N1’-N7”	1.376
N7”-H7”	1.010	N7”-H7”	1.010	N7”-H7”	1.010	N7”-H7”	1.010	N7”-H7”	1.011
N7”-C7”	1.371	N7”-C7”	1.383	N7”-C7”	1.383	N7”-C7”	1.368	N7”-C7”	1.383
C7”-O7”	1.227	C7”-O7”	1.214	C7”-O7”	1.215	C7”-O7”	1.227	C7”-O7”	1.214
C7”-C1”	1.480	C7”-C1”	1.486	C7”-C1”	1.487	C7”-C1”	1.481	C7”-C1”	1.487
C1”-C2”	1.410	C1”-C2”	1.396	C1”-C2”	1.402	C1”-C2”	1.400	C1”-C2”	1.400
C1”-C6”	1.404	C1”-C6”	1.398	C1”-C6”	1.391	C1”-C6”	1.405	C1”-C6”	1.392
C2”-C3”	1.398	C2”-C3”	1.385	C2”-C3”	1.381	C2”-C3”	1.404	C2”-C3”	1.383
C2”-O2”	1.345	C2”-H2”	1.083	C2”-H2”	1.082	C2”-O2”	1.350	C2”-H2”	1.085
O2”-H2”	0.977	C3”-H3”	1.085	C3”-O3”	1.357	O2”-H2”	0.978	C3”-O3”	1.362
C3”-H3”	1.083	C3”-C4”	1.396	O3”-C8”	1.423	C3”-O3”	1.356	O3”-H3”	0.963
C3”-C4”	1.383	C4”-C5”	1.395	C8”-H8”(1)	1.092	O3”-H3”	0.966	C3”-C4”	1.403
C4”-H4”	1.084	C4”-O4”	1.354	C8”-H8”(2)	1.086	C3”-C4”	1.382	C4”-O4”	1.352
C4”-C5”	1.398	O4”-H4”	0.963	C8”-H8”(3)	1.092	C4”-H4”	1.083	O4”-H4”	0.966
C5”-C6”	1.382	C5”-C6”	1.385	C3”-C4”	1.409	C4”-C5”	1.397	C4”-C5”	1.388
C5”-H5”	1.082	C5”-H5”	1.083	C4”-C5”	1.384	C5”-H5”	1.082	C5”-C6”	1.398
C6”-H6”	1.084	C6”-H6”	1.084	C4”-O4”	1.350	C5”-C6”	1.382	C5”-H5”	1.083
				O4”-H4”	0.967	C6”-H6”	1.083	C6”-H6”	1.082
				C5”-C6”	1.392				
				C5”-H5”	1.083				
				C6”-H6”	1.083				

Table S9: Bond angles of the investigated coumarin-hydroxybenzohydrazide derivatives

**3a**		**3b**		**3c**		**3d**		**3e**	
**Bond angle (º)**	**M062X**	**Bond angle (º)**	**M062X**	**Bond angle (º)**	**M062X**	**Bond angle (º)**	**M062X**	**Bond angle (º)**	**M062X**
O1-C2-O2	114.84	O1-C2-O2	114.78	O1-C2-O2	114.79	O1-C2-O2	114.86	O1-C2-O2	114.79
O1-C2-C3	118.06	O1-C2-C3	118.06	O1-C2-C3	118.04	O1-C2-C3	118.04	O1-C2-C3	118.06
O1-C9-C10	121.82	O1-C9-C10	121.79	O1-C9-C10	118.04	O1-C9-C10	121.85	O1-C9-C10	121.80
O1-C9-C8	116.88	O1-C9-C8	116.90	O1-C9-C8	116.86	O1-C9-C8	116.83	O1-C9-C8	116.89
O2-C2-C3	127.10	O2-C2-C3	127.15	O2-C2-C3	127.16	O2-C2-C3	127.09	O2-C2-C3	127.15
C2-C3-C4	120.38	C2-C3-C4	120.40	C2-C3-C4	120.41	C2-C3-C4	120.38	C2-C3-C4	120.40
C2-C3-C1’	118.77	C2-C3-C1’	118.78	C2-C3-C1’	118.80	C2-C3-C1’	117.76	C2-C3-C1’	117.78
C3-C4-O4	123.83	C3-C4-O4	123.87	C3-C4-O4	123.89	C3-C4-O4	123.82	C3-C4-O4	123.86
C3-C1’-N1’	118.11	C3-C1’-N1’	118.21	C3-C1’-N1’	118.14	C3-C1’-N1’	118.09	C3-C1’-N1’	118.18
C3-C1’-C2’	123.92	C3-C1’-C2’	123.87	C3-C1’-C2’	123.84	C3-C1’-C2’	123.91	C3-C1’-C2’	123.91
C3-C4-C10	116.76	C3-C4-C10	116.77	C3-C4-C10	116.78	C3-C4-C10	116.76	C3-C4-C10	116.78
C4-C10-C9	119.59	C4-C10-C9	119.59	C4-C10-C9	119.59	C4-C10-C9	119.58	C4-C10-C9	119.58
O4-C4-C10	119.41	O4-C4-C10	119.36	O4-C4-C10	119.32	O4-C4-C10	119.42	O4-C4-C10	119.36
C4-C10-C5	121.32	C4-C10-C5	121.31	C4-C10-C5	121.31	C4-C10-C5	121.35	C4-C10-C5	121.35
C10-C5-C6	120.34	C10-C5-C6	120.34	C10-C5-C6	120.34	C10-C5-C6	120.34	C10-C5-C6	120.35
C10-C9-C8	121.30	C10-C9-C8	121.30	C10-C9-C8	121.34	C10-C9-C8	121.32	C10-C9-C8	121.31
C5-C6-C7	119.66	C5-C6-C7	119.67	C5-C6-C7	119.68	C5-C6-C7	119.67	C5-C6-C7	119.67
C5-C10-C9	119.09	C5-C10-C9	119.07	C5-C10-C9	119.06	C5-C10-C9	119.07	C5-C10-C9	119.07
C6-C7-C8	120.77	C6-C7-C8	120.76	C6-C7-C8	120.76	C6-C7-C8	120.75	C6-C7-C8	120.75

C7-C8-C9	118.84	C7-C8-C9	118.84	C7-C8-C9	118.83	C7-C8-C9	118.84	C7-C8-C9	118.85
C1’-N1’-N7”	123.15	C1’-N1’-N7”	123.10	C1’-N1’-N7”	123.08	C1’-N1’-N7”	123.12	C1’-N1’-N7”	123.11
N1’-N7”-C7”	118.96	N1’-N7”-C7”	118.86	N1’-N7”-C7”	118.71	N1’-N7”-C7”	119.30	N1’-N7”-C7”	118.83
N7”-C7”-O7”	120.18	N7”-C7”-O7”	121.18	N7”-C7”-O7”	121.17	N7”-C7”-O7”	120.44	N7”-C7”-O7”	121.31
N7”-C7”-C1”	116.91	N7”-C7”-C1”	115.09	N7”-C7”-C1”	115.03	N7”-C7”-C1”	116.86	N7”-C7”-C1”	114.93
C7”-C1”-C2”	118.38	C7”-C1”-C2”	117.61	C7”-C1”-C2”	122.09	C7”-C1”-C6”	123.25	C7”-C1”-C2”	122.20
C7”-C1”-C6”	122.63	C7”-C1”-C6”	123.08	C7”-C1”-C6”	117.56	C7”-C1”-C2”	117.46	C7”-C1”-C6”	117.72
C1”-C2”-C3”	119.56	C1”-C2”-C3”	120.65	C1”-C2”-C3”	119.36	C1”-C2”-C3”	119.83	C1”-C2”-C3”	119.58
C1”-C6”-C5”	121.12	C1”-C6”-C5”	120.46	C1”-C6”-C5”	120.21	C1”-C6”-C5”	120.31	C1”-C6”-C5”	120.20
C2”-C3”-C4”	120.30	C2”-C3”-C4”	119.55	C2”-C3”-C4”	120.09	C2”-C3”-C4”	120.18	C2”-C3”-C4”	120.26
C3”-C4”-C5”	120.70	C3”-C4”-C5”	120.31	C3”-C4”-C5”	120.19	C3”-C4”-C5”	120.07	C3”-C4”-C5”	119.95
C4”-C5”-C6”	119.28	C4”-C5”-C6”	119.71	C4”-C5”-C6”	119.80	C4”-C5”-C6”	120.31	C4”-C5”-C6”	119.92
O2”-C2”-C3”	117.30	O4”-C4”-C3”	122.42	O4”-C4”-C3”	119.71	O2”-C2”-C3”	115.24	O4”-C4”-C3”	120.14
O2”-C2”-C1”	123.14	O4”-C4”-C5”	117.26	O4”-C4”-C5”	120.09	O2”-C2”-C1”	124.92	O4”-C4”-C5”	119.91
				C8”-O3”-C3”	117.92	O3”-C3”-C4”	120.30	O3”-C3”-C4”	114.74
				O3”-C3”-C4”	113.85	O3”-C3”-C2”	119.52	O3”-C3”-C2”	124.99
				O3”-C3”-C2”	126.06				

Table S10: Dihedral angles of the investigated coumarin-hydroxybenzohydrazide derivatives

3a		3b		3c		3d		3e	
**Dihedral angle (°)**	**M062X**	**Dihedral angle (°)**	**M062X**	**Dihedral angle (°)**	**M062X**	**Dihedral angle (°)**	**M062X**	**Dihedral angle (°)**	**M062X**
O1-C2-C3-C1’	178.81	O1-C2-C3-C1’	178.31	O1-C2-C3-C1’	179.06	O1-C2-C3-C1’	179.12	O1-C2-C3-C1’	178.36
O2-C2-C3-C1’	1.49	O2-C2-C3-C1’	-2.10	O2-C2-C3-C1’	-1.16	O2-C2-C3-C1’	-1.22	O2-C2-C3-C1’	-2.04
O1-C2-C3-C4	-2.91	O1-C2-C3-C4	-3.68	O1-C2-C3-C4	-2.82	O1-C2-C3-C4	-2.58	O1-C2-C3-C4	-3.56
C2-C3-C1’-N1’	177.60	C2-C3-C1’-N1’	176.19	C2-C3-C1’-N1’	177.56	C2-C3-C1’-N1’	177.83	C2-C3-C1’-N1’	176.20
C2-C3-C4-O4	-178.21	C2-C3-C4-O4	-177.60	C2-C3-C4-O4	-178.42	C2-C3-C4-O4	-178.20	C2-C3-C4-O4	-177.72
C2-C3-C1’-C2’	-3.01	C2-C3-C1’-C2’	-4.54	C2-C3-C1’-C2’	-3.04	C2-C3-C1’-C2’	-2.82	C2-C3-C1’-C2’	-4.50
C2-C3-C4-C10	2.09	C2-C3-C4-C10	2.81	C2-C3-C4-C10	1.74	C2-C3-C4-C10	2.05	C2-C3-C4-C10	2.63
C3-C1’-N1’-N7”	-172.82	C3-C1’-N1’-N7”	-172.94	C3-C1’-N1’-N7”	-172.39	C3-C1’-N1’-N7”	-172.80	C3-C1’-N1’-N7”	-172.93
C4-C10-C5-C6	-179.52	C4-C10-C5-C6	-179.62	C4-C10-C5-C6	-179.65	C4-C10-C5-C6	-179.56	C4-C10-C5-C6	-179.73
C4-C10-C9-C8	179.45	C4-C10-C9-C8	179.54	C4-C10-C9-C8	179.58	C4-C10-C9-C8	179.55	C4-C10-C9-C8	179.68
C10-C5-C6-C7	0.04	C10-C5-C6-C7	0.06	C10-C5-C6-C7	0.06	C10-C5-C6-C7	0.01	C10-C5-C6-C7	0.04
C10-C9-C8-C7	0.11	C10-C9-C8-C7	0.12	C10-C9-C8-C7	0.11	C10-C9-C8-C7	0.04	C10-C9-C8-C7	0.05
C5-C6-C7-C8	-0.05	C5-C6-C7-C8	-0.02	C5-C6-C7-C8	-0.06	C5-C6-C7-C8	-0.03	C5-C6-C7-C8	-0.01
C6-C7-C8-C9	-0.02	C6-C7-C8-C9	-0.07	C6-C7-C8-C9	-0.02	C6-C7-C8-C9	0.01	C6-C7-C8-C9	0.04
C7-C8-C9-O1	-179.69	C7-C8-C9-O1	-179.59	C7-C8-C9-O1	-179.63	C7-C8-C9-O1	-179.80	C7-C8-C9-O1	-179.63
C9-C10-C4-O4	-179.99	C9-C10-C4-O4	-179.78	C9-C10-C4-O4	-179.84	C9-C10-C4-O4	179.76	C9-C10-C4-O4	179.79
C1’-N1’-N7”-C7”	76.51	C1’-N1’-N7”-C7”	75.27	C1’-N1’-N7”-C7”	74.71	C1’-N1’-N7”-C7”	75.26	C1’-N1’-N7”-C7”	75.00
N1’-N7”-C7”-O1”	-7.73	N1’-N7”-C7”-O7”	-8.49	N1’-N7”-C7”-O7”	-8.71	N1’-N7”-C7”-O7”	-6.50	N1’-N7”-C7”-O7”	-8.59
N1’-N7”-C7”-C1”	173.67	N1’-N7”-C7”-C1”	173.37	N1’-N7”-C7”-C1”	173.12	N1’-N7”-C7”-C1”	175.13	N1’-N7”-C7”-C1”	173.19
N7”-C7”-C1”-C6”	14.01	N7”-C7”-C1”-C6”	22.74	N7”-C7”-C1”-C6”	-155.92	N7”-C7”-C1”-C6”	12.72	N7”-C7”-C1”-C6”	-156.05
N7”-C7”-C1”-C2”	-166.20	N7”-C7”-C1”-C2”	-158.36	N7”-C7”-C1”-C2”	-24.66	N7”-C7”-C1”-C2”	-168.01	N7”-C7”-C1”-C2”	24.54
C7”-C1”-C2”-O2”	-1.80	C7”-C1”-C6”-C5”	-179.55	C7”-C1”-C6”-C5”	-179.46	C7”-C1”-C6”-C5”	-179.51	C7”-C1”-C6”-C5”	179.39
C7”-C1”-C6”-C5”	-178.60	C7”-C1”-C2”-C3”	179.85	C7”-C1”-C2”-C3”	179.95	C7”-C1”-C2”-C3”	178.93	C7”-C1”-C2”-C3”	-179.84
C7”-C1”-C2”-C3”	177.76	C1”-C2”-C3”-C4”	0.79	C1”-C2”-C3”-C4”	0.31	C1”-C2”-C3”-C4”	1.14	C1”-C2”-C3”-C4”	0.10
C1”-C2”-C3”-C4”	1.57	C1”-C6”-C5”-C4”	0.26	C1”-C6”-C5”-C4”	0.79	C1”-C6”-C5”-C4”	-0.04	C1”-C6”-C5”-C4”	0.74

C1”-C6”-C5”-C4”	0.14	C6”-C5”-C4”-C3”	-0.69	C6”-C5”-C4”-C3”	-0.06	C6”-C5”-C4”-C3”	-0.61	C6”-C5”-C4”-C3”	0.13
O2”-C2”-C3”-C4”	-178.85	C2”-C3”-C4”-C5”	0.16	C2”-C3”-C4”-C5”	0.62	C2”-C3”-C4”-C5”	0.64	C2”-C3”-C4”-C5”	-0.55
O2”-C2”-C1”-C6”	178.01	O4”-C4”-C3”-C2”	179.85	O4”-C4”-C3”-C2”	179.84	O2”-C2”-C3”-C4”	-179.19	O3”-C3”-C2”-C1”	-179.19
C6”-C5”-C4”-C3”	-1.06	O4”-C4”-C5”-C6”	179.85	O4”-C4”-C5”-C6”	179.60	O2”-C2”-C1”-C7”	-0.71	O3”-C3”-C4”-C5”	178.81
C2”-C3”-C4”-C5”	0.20			C8”-O3”-C3”-C2”	-0.08	O3”-C3”-C2”-C1”	-179.36	O4”-C4”-C3”-C2”	179.86
				C8”-O3”-C3”-C4”	-179.48	O3”-C3”-C2”-O2”	0.31	O3”-C3”-C4”-O4”	-0.78
				O3”-C3”-C4”-C5”	178.82	O3”-C3”-C4”-C5”	-179.43	O4”-C4”-C5”-C6”	179.72
				O3”-C3”-C2”-C1”	-179.05				
				O3”-C3”-C4”-O4”	-0.72				

Optimized geometries of investigated compounds and radicals, anions and radical cations in methanol.

Ground states

3a
H(UM06-2X)=-1179.556536 Ha
Charge = 0; Multiplicity =1

C	2.52911600	1.77958600	-0.12612600
C	4.45974600	0.34037800	-0.22830700
C	3.73615900	-0.79105000	0.12478200
C	2.29348500	-0.67530000	0.40055400
C	1.71124800	0.64787600	0.28777800
H	6.35915700	1.17658700	-0.75763200
C	5.82692100	0.27385900	-0.48561500
C	4.39875900	-2.02027600	0.21995200
C	5.75553900	-2.10388700	-0.03374700
C	6.46663600	-0.95075200	-0.38651000
H	3.82162200	-2.89390900	0.49716400
H	6.26708200	-3.05509000	0.04081900
O	3.86977100	1.56351600	-0.34374900
O	2.16141300	2.91529800	-0.31130800
O	1.65095200	-1.68734500	0.71560100
C	0.33102100	0.85912500	0.54110700
C	-0.33438600	2.19516700	0.47214800
H	-0.34339500	2.54224900	-0.56224900
H	-1.34995700	2.14007400	0.85222500
H	0.24301200	2.91988200	1.04199900
N	-0.41350800	-0.19192200	0.87083200
H	0.04885300	-1.11097600	0.88074100
N	-1.78199400	-0.13419100	0.99825900
C	-2.55384900	-0.10584200	-0.13483200
H	-2.14531400	-0.46808100	1.87939300
C	-4.01970200	-0.18936500	0.05101200
C	-4.82353100	-0.45982100	-1.07498300
C	-4.63605100	-0.00619100	1.29881900
C	-6.20665800	-0.59080600	-0.91949500
C	-6.00584300	-0.11932000	1.44236400
H	-4.04275400	0.25880100	2.16611200

C	-6.78776200	-0.42435200	0.32408700
O	-2.02749300	0.01109000	-1.23698300
H	7.52991400	-1.01142100	-0.58530400
H	-7.86233700	-0.52082500	0.42646700
H	-6.46557400	0.03302200	2.41005700
H	-6.80113900	-0.81434500	-1.79687700
O	-4.32520000	-0.61066500	-2.31468800
H	-3.37130300	-0.40251600	-2.28546300

3b

H(UM06-2X)= -1179.553752 Ha

Charge = 0; Multiplicity =1

C	2.71526300	1.76068800	-0.08144600
C	4.65493300	0.33313100	-0.18189100
C	3.92215700	-0.81570500	0.08547500
C	2.46932100	-0.71607100	0.31136300
C	1.88911400	0.60980200	0.25107000
H	6.56934100	1.19807500	-0.59795900
C	6.03099600	0.28169300	-0.39149100
C	4.58477700	-2.04713800	0.14266400
C	5.95026900	-2.11597200	-0.06482100
C	6.67039800	-0.94562500	-0.33204800
H	4.00104000	-2.93450600	0.35467200
H	6.46194300	-3.06895800	-0.01986800
O	4.06513400	1.55934400	-0.25783100
O	2.34940400	2.90242900	-0.23232800
O	1.81839400	-1.74528000	0.54569500
C	0.49862300	0.80650600	0.47233200
C	-0.16191500	2.14687200	0.48063500
H	-0.18041200	2.54730900	-0.53431400
H	-1.17552800	2.07021200	0.86330300
H	0.42093100	2.83828300	1.08447100
N	-0.25758400	-0.26008300	0.69888500
H	0.20394200	-1.17851000	0.66477100
N	-1.62986000	-0.20377700	0.78536700
C	-2.36666000	-0.06072900	-0.37640300
H	-2.02037900	-0.67412300	1.58970900
C	-3.83962100	-0.16379500	-0.20544500
C	-4.60011500	-0.49014100	-1.32956200
C	-4.48495100	0.07180600	1.01260600
C	-5.97763800	-0.60573500	-1.24096200
H	-4.09919800	-0.66014600	-2.27489400
C	-5.86277400	-0.03068700	1.10851200
H	-3.92450800	0.36982300	1.89126900
C	-6.61063800	-0.37693400	-0.01768500
O	-1.82614400	0.16737700	-1.43973700
O	-7.95262900	-0.47085000	0.13390800
H	-8.36966000	-0.71140100	-0.70070500
H	7.74039500	-0.99483800	-0.49467700
H	-6.56400200	-0.87115300	-2.11394200

H	-6.37432500	0.16099100	2.04330900

3c

H(UM06-2X)= -1294.033856 Ha

Charge = 0; Multiplicity =1

C	-3.31078000	1.60202700	0.72521100
C	-5.18876400	0.12330000	0.41090000
C	-4.44225400	-0.80211900	-0.30568000
C	-3.01726800	-0.53749200	-0.57206100
C	-2.47709300	0.70510300	-0.06111200
H	-7.09204600	0.66232700	1.23137000
C	-6.54193600	-0.08220600	0.66986400
C	-5.06653400	-1.96470100	-0.77226100
C	-6.40877500	-2.18498600	-0.52266300
C	-7.14389900	-1.23781700	0.19977500
H	-4.47144100	-2.67656700	-1.33098900
H	-6.89049500	-3.08432500	-0.88472000
O	-4.63541200	1.26938400	0.89706800
O	-2.97223000	2.62873400	1.26516800
O	-2.35568300	-1.36736300	-1.21417800
C	-1.11496700	1.05011000	-0.27999700
C	-0.49420400	2.32803400	0.18200200
H	-1.13813400	3.16466500	-0.07779500
H	-0.40992700	2.31436500	1.26999100
H	0.49041400	2.45508300	-0.25905900
N	-0.35117100	0.19133500	-0.94289400
H	-0.78377300	-0.69919400	-1.22498900
N	1.00790700	0.35025900	-1.08919700
C	1.82801400	0.10944800	-0.00247000
H	1.34912400	0.21364500	-2.03033000
C	3.28610400	0.14104100	-0.29383000
C	4.12441800	-0.52371900	0.61187500
C	3.82409800	0.80775800	-1.39018400
C	5.48935700	-0.53452500	0.39748400
H	3.68202300	-1.02307900	1.46360200
C	5.20081200	0.80513800	-1.59871900
H	3.19364900	1.36473000	-2.07248500
C	6.03193900	0.13493400	-0.71745200
O	1.36561900	-0.07624800	1.10526500
O	7.36709000	0.12559100	-0.91987600
H	7.78310800	-0.37885600	-0.20713700
O	6.41617600	-1.14591500	1.17707300
H	-8.19589500	-1.40637900	0.39660300
C	5.95415100	-1.84407000	2.32753400
H	5.44862000	-1.16024100	3.01347100
H	5.27744800	-2.65175000	2.03898200
H	6.83910300	-2.25709800	2.80367500
H	5.64207300	1.32651400	-2.43900000

3d

H(UM06-2X)= -1254.779242 Ha
Charge = 0; Multiplicity =1

C	-2.76659300	1.68436200	-0.52047300
C	-4.76293000	0.49309000	0.11431900
C	-4.10140100	-0.72231700	0.22597300
C	-2.65826500	-0.79501100	-0.06331400
C	-2.01144500	0.43875500	-0.47082300
H	-6.60993200	1.56539900	0.26867600
C	-6.12831800	0.60067200	0.36720700
C	-4.82642900	-1.85919100	0.60250400
C	-6.18245200	-1.76955400	0.85809900
C	-6.83017300	-0.53430700	0.73831500
H	-4.29739900	-2.80078000	0.68462900
H	-6.74270900	-2.64935500	1.14820800
O	-4.11217200	1.63616800	-0.24236100
O	-2.33958500	2.78438600	-0.77803900
O	-2.07094200	-1.88056800	0.04160400
C	-0.63291100	0.46370300	-0.79892100
C	0.08809800	1.68371600	-1.27202700
H	0.15634700	2.40229300	-0.45404700
H	1.08400300	1.43463300	-1.62575500
H	-0.48327400	2.16085900	-2.06528700
N	0.05702300	-0.67124500	-0.69911800
H	-0.44185700	-1.49990700	-0.35258400
N	1.41787300	-0.74954900	-0.86703100
C	2.25436400	-0.25020800	0.08694200
H	1.74499400	-1.20789100	-1.70411400
C	3.70791200	-0.46288800	-0.11090600
C	4.58422000	0.25783300	0.71056700
C	4.23678700	-1.34327500	-1.07041400
C	5.97273900	0.11554100	0.54768600
C	5.60367400	-1.47893900	-1.21668800
H	3.58658200	-1.94724700	-1.69026600
C	6.47565900	-0.74358200	-0.40973900
O	1.80099300	0.37677500	1.04155200
O	6.81328500	0.82517800	1.34020200
H	6.28746100	1.34653500	1.96138200
H	-7.89264600	-0.46019300	0.93686500
O	4.20425300	1.11564700	1.67914300
H	3.22864300	1.07326900	1.75314400
H	6.00348000	-2.16371800	-1.95313800
H	7.55016100	-0.83943700	-0.51132800

3e
H(UM06-2X)= -1254.775512Ha
Charge = 0; Multiplicity =1

C	2.99664200	1.75796400	-0.13712000
C	4.94173600	0.33463400	-0.11980500
C	4.20253000	-0.80727600	0.15909200
C	2.74237100	-0.70524500	0.32815100

C	2.16129100	0.61536700	0.20087900
H	6.86752500	1.19158100	-0.49824000
C	6.32432600	0.28074400	-0.28024500
C	4.86560900	-2.03397000	0.27984600
C	6.23761100	-2.10504600	0.12252800
C	6.96412600	-0.94173500	-0.15803800
H	4.27667900	-2.91598700	0.49975900
H	6.74903000	-3.05458200	0.21678900
O	4.35269300	1.55580700	-0.25589700
O	2.63400000	2.89304800	-0.33819000
O	2.08571200	-1.72837800	0.57316900
C	0.76275500	0.81372500	0.36213100
C	0.09794100	2.15051400	0.30090700
H	0.11366200	2.51537000	-0.72751300
H	-0.92842700	2.08221800	0.64979400
H	0.65630400	2.86549900	0.90023000
N	0.00186000	-0.24827800	0.59444400
H	0.46772200	-1.16533400	0.60813200
N	-1.37299400	-0.19566000	0.62629700
C	-2.06435400	-0.08812700	-0.56705700
H	-1.79233800	-0.65468500	1.42301500
C	-3.54288400	-0.19737500	-0.44707800
C	-4.21948900	0.10572800	0.74088000
C	-4.25566800	-0.60520300	-1.57169200
C	-5.59584200	-0.01803400	0.79326000
H	-3.69166500	0.47412100	1.61400000
C	-5.63694500	-0.73687500	-1.51347900
H	-3.72104900	-0.82745200	-2.48633900
C	-6.31083000	-0.44659400	-0.33562100
O	-1.48608700	0.11402000	-1.61492800
O	-7.65580400	-0.56949900	-0.28069700
H	-7.95908400	-0.32934900	0.60494800
O	-6.36420200	0.26168100	1.88216100
H	-5.82760800	0.52823800	2.63633400
H	8.03927400	-0.99273700	-0.28193000
H	-6.20812800	-1.06194300	-2.37434900

Radicals

3aC2”O•
H(UM06-2X)= -1178.910820 Ha
Charge = 0; Multiplicity =2

C	-2.52102800	1.75398800	0.32954000
C	-4.46651300	0.33676900	0.21105600
C	-3.73636100	-0.76926200	-0.20294500
C	-2.28130100	-0.64799000	-0.40023000
C	-1.69624200	0.65637400	-0.15688200
H	-6.38164500	1.14894000	0.72032900
C	-5.84497000	0.26583200	0.39716000
C	-4.40407000	-1.97765900	-0.43340100

C	-5.77207500	-2.06553400	-0.25192700
C	-6.48971100	-0.93769600	0.16364000
H	-3.82126300	-2.83218500	-0.75482200
H	-6.28713300	-3.00078800	-0.43059700
O	-3.87265200	1.53751300	0.46221200
O	-2.15003000	2.85967500	0.64452500
O	-1.63194900	-1.63845200	-0.76543600
C	-0.30922100	0.87965900	-0.35562100
C	0.35630500	2.20547500	-0.17747400
H	-0.22851200	2.97998500	-0.66738800
H	0.38886800	2.45275600	0.88509500
H	1.36522700	2.18517000	-0.57866000
N	0.44287300	-0.14821800	-0.73896500
H	-0.02047000	-1.06163900	-0.83054800
N	1.81267200	-0.08774400	-0.83666900
C	2.57987800	-0.06761100	0.30282500
C	4.04987300	-0.10268700	0.02974400
C	4.86510300	-1.09315600	0.73081200
C	4.63027200	0.79812600	-0.82516500
C	6.29653600	-1.04458100	0.49635100
C	6.02672600	0.80141100	-1.02055800
H	4.02239200	1.53118500	-1.34373900
C	6.84852500	-0.12144600	-0.35358100
O	2.10021300	0.03729300	1.40927800
H	-7.56177300	-1.00186700	0.30652400
H	7.91844600	-0.10110500	-0.51883000
H	6.46464000	1.52871400	-1.69223500
H	2.20765900	-0.33868600	-1.73317200
O	4.36116000	-1.95605700	1.46830200
H	6.90044200	-1.77858600	1.01637200

3aC7”N•
H(UM06-2X)= -1178.916194 Ha
Charge = 0; Multiplicity =2

C	-2.86912800	1.92339500	0.03247500
C	-4.50250400	0.15644500	-0.01354000
C	-3.54629400	-0.85071900	0.00255600
C	-2.11806900	-0.50795700	0.02720500
C	-1.78537700	0.92566600	0.01984000
H	-6.57877900	0.67428000	-0.05417800
C	-5.86229300	-0.13704500	-0.04253000
C	-3.96451900	-2.18754000	-0.00972500
C	-5.31116400	-2.49590700	-0.03839600
C	-6.25767200	-1.46474100	-0.05489400
H	-3.20797200	-2.96240700	0.00334800
H	-5.63455700	-3.52889000	-0.04820700
O	-4.16110200	1.47902400	0.00201700
O	-2.72761200	3.11783100	0.06910100
O	-1.26948400	-1.39926900	0.04728500
C	-0.46578100	1.38352000	0.00357400

C	-0.04296300	2.81601200	-0.02660100
H	-0.51610100	3.32300400	-0.86671900
H	-0.39114900	3.31749300	0.87726800
H	1.03689000	2.88667900	-0.09907500
N	0.52362400	0.44676000	0.01377600
H	0.23054400	-0.54912300	0.03627100
N	1.79328000	0.75050900	-0.00250200
C	2.58425800	-0.40400400	0.01505900
C	4.03082200	-0.16053600	-0.00424600
C	4.91738600	-1.25972200	0.00740000
C	4.55651400	1.14387100	-0.03401000
C	6.29680900	-1.03343500	-0.01113600
C	5.91987500	1.35811900	-0.05173700
H	3.86823100	1.97888400	-0.04280800
C	6.78748400	0.25909300	-0.04015100
O	2.08800500	-1.53247800	0.04478700
H	-7.31444400	-1.70240000	-0.07726700
H	7.85934200	0.41864300	-0.05398500
H	6.31379200	2.36572800	-0.07454700
H	6.95863200	-1.89064400	-0.00216900
O	4.49905400	-2.53521900	0.03553000
H	3.52205600	-2.53833100	0.04652700

3bC4”O•
H(UM06-2X)= -1178.912715 Ha
Charge = 0; Multiplicity =2

C	-2.65469900	1.75987300	0.09403700
C	-4.60085000	0.34096900	0.18268000
C	-3.87405200	-0.81017200	-0.09154100
C	-2.42079200	-0.71722300	-0.31411400
C	-1.83339900	0.60619100	-0.24500600
H	-6.51095300	1.21329900	0.60282700
C	-5.97725300	0.29554200	0.39059900
C	-4.54321500	-2.03766800	-0.15807300
C	-5.90926900	-2.10031900	0.04709000
C	-6.62317500	-0.92788200	0.32179000
H	-3.96406700	-2.92676800	-0.37529400
H	-6.42612300	-3.05011100	-0.00524700
O	-4.00487400	1.56397000	0.26743900
O	-2.28164000	2.89781300	0.25273200
O	-1.77397500	-1.74744000	-0.55336500
C	-0.44432700	0.79712600	-0.46431800
C	0.22458800	2.13320400	-0.46671100
H	-0.35645200	2.83191500	-1.06375100
H	0.25024500	2.52723000	0.55064700
H	1.23589500	2.05529500	-0.85553500
N	0.30572500	-0.27550500	-0.69651500
H	-0.16206200	-1.19198600	-0.67085500
N	1.67762800	-0.22995900	-0.78072100
C	2.41212800	-0.06794800	0.37029400

C	3.89278800	-0.17559100	0.20076800
C	4.63747800	-0.55895200	1.33785200
C	4.53507900	0.12233500	-1.01930100
C	5.99608600	-0.67956200	1.26148200
H	4.10765100	-0.76109900	2.26041200
C	5.89670300	0.01889400	-1.11827900
H	3.96121000	0.46206600	-1.87336400
C	6.69616800	-0.39785900	0.02039600
O	1.89390300	0.18265500	1.43743600
O	7.93318800	-0.50351300	-0.06479000
H	-7.69359700	-0.97233200	0.48289900
H	6.59133000	-0.98292100	2.11427800
H	6.41918100	0.24969900	-2.03857600
H	2.07152600	-0.66966800	-1.60126700

3bC7”N•

H(UM06-2X)= -1178.922555 Ha

Charge = 0; Multiplicity =2

C	-2.97689000	1.90417800	0.04229000
C	-4.67499600	0.20027800	-0.03443000
C	-3.75750300	-0.84147500	0.00180300
C	-2.31727500	-0.55085800	0.05606200
C	-1.93226800	0.86740300	0.04102600
H	-6.72902100	0.79557600	-0.11966500
C	-6.04397600	-0.04218000	-0.09386500
C	-4.22531200	-2.16142800	-0.02167400
C	-5.58167200	-2.41955200	-0.08137400
C	-6.48870000	-1.35398600	-0.11741900
H	-3.49828900	-2.96362100	0.00720100
H	-5.94274000	-3.43975500	-0.10045400
O	-4.28520300	1.50861500	-0.00819900
O	-2.79349000	3.09313400	0.08730100
O	-1.50448900	-1.47446500	0.10187100
C	-0.59432400	1.27511600	0.02542500
C	-0.12116200	2.69149200	-0.02446700
H	-0.58395400	3.20670800	-0.86518000
H	-0.44195200	3.21440700	0.87748500
H	0.95976200	2.72238900	-0.10749300
N	0.36073900	0.30620900	0.05248600
H	0.03695700	-0.67879400	0.09267000
N	1.64168800	0.56048900	0.02658500
C	2.38717700	-0.63773800	0.06569300
C	3.84754300	-0.43514300	0.02111400
C	4.67573300	-1.56197500	0.04608500
C	4.42578200	0.83925600	-0.04741400
C	6.05188200	-1.42820100	0.00372900
H	4.22608800	-2.54603800	0.09951400
C	5.80117600	0.98264200	-0.08941300
H	3.79287100	1.71691000	-0.06724400
C	6.61668900	-0.15086300	-0.06402500

O	1.85372700	-1.73345000	0.12961300
O	7.95309900	0.04772800	-0.10785000
H	8.42129500	-0.79414600	-0.08085000
H	-7.55271900	-1.55203700	-0.16407300
H	6.69187800	-2.30371400	0.02111700
H	6.26225900	1.96095500	-0.14330200

3cC4”O•

H(UM06-2X)= -1293.394502 Ha

Charge = 0; Multiplicity =2

C	-3.34227800	1.68136900	0.47804700
C	-5.23059900	0.18669100	0.38156200
C	-4.48501100	-0.85186100	-0.15957100
C	-3.05204000	-0.65019000	-0.43666700
C	-2.50692000	0.65877700	-0.13613100
H	-7.13843500	0.87332600	1.06960700
C	-6.59026500	0.04127200	0.64596400
C	-5.11781900	-2.06834800	-0.44113900
C	-6.46698100	-2.22951700	-0.18428200
C	-7.20056000	-1.16881100	0.36006000
H	-4.52362500	-2.86932500	-0.86357800
H	-6.95572500	-3.17055100	-0.40221600
O	-4.67085800	1.39133400	0.68547900
O	-2.99868200	2.78025800	0.84408400
O	-2.38744900	-1.58201500	-0.91366100
C	-1.14275400	0.95465600	-0.39615400
C	-0.52174600	2.29390300	-0.16613600
H	-1.16535900	3.06964500	-0.57374400
H	-0.44226100	2.47688900	0.90671600
H	0.46358800	2.34360300	-0.62040600
N	-0.37553900	-0.01178800	-0.88930600
H	-0.80915400	-0.93578800	-1.02040500
N	0.98514000	0.11568100	-1.04430000
C	1.79237200	0.07145600	0.07050100
C	3.25898300	0.06963600	-0.21338000
C	4.08047200	-0.44569200	0.76529700
C	3.80467300	0.59067400	-1.42467700
C	5.46870200	-0.49725600	0.58691000
H	3.66121700	-0.82692000	1.68845000
C	5.14674700	0.56317300	-1.62988000
H	3.15767300	1.04038100	-2.16814200
C	6.06905500	0.01232500	-0.65484300
O	1.33403200	0.06957900	1.19324400
O	7.29405700	-0.01860200	-0.86307600
O	6.15055900	-1.02345500	1.58877800
H	-8.25782500	-1.29142900	0.56247400
C	7.58186900	-1.15159200	1.60350700
H	7.91386800	-1.79198400	0.78923600
H	8.05146200	-0.17354600	1.52105800
H	7.80063700	-1.60537700	2.56620200

H	5.59098800	0.96294300	-2.53332900
H	1.33706100	-0.12990400	-1.95939200

3cC7"N•

H(UM06-2X)= -1293.395122 Ha

Charge = 0; Multiplicity =2

C	-3.73220800	1.83309100	0.10790900
C	-5.29969500	0.00625000	0.08004100
C	-4.30868500	-0.96151700	-0.02112300
C	-2.89488300	-0.56222400	-0.06629800
C	-2.61535700	0.87991600	0.00780300
H	-7.39125200	0.44309200	0.20855800
C	-6.64678900	-0.33868800	0.12774000
C	-4.67878100	-2.31137000	-0.07769700
C	-6.01230100	-2.67087000	-0.03169900
C	-6.99440900	-1.67845400	0.07135800
H	-3.89599500	-3.05551700	-0.15836900
H	-6.29807200	-3.71407700	-0.07498200
O	-5.00676300	1.33855600	0.13661100
O	-3.63764900	3.03169200	0.16972800
O	-2.01543600	-1.41855700	-0.16058100
C	-1.31256900	1.38723800	-0.00690200
C	-0.94608900	2.83438600	0.06288700
H	-1.42547000	3.37353200	-0.75386100
H	-1.32895900	3.26554500	0.98835700
H	0.13091300	2.95066500	0.01271200
N	-0.28711000	0.49544200	-0.08768100
H	-0.53507900	-0.51031700	-0.14782300
N	0.96944000	0.85159900	-0.08348100
C	1.81105200	-0.27609600	-0.17093100
C	3.24968600	0.05401100	-0.14712400
C	4.17236600	-0.99652600	-0.21549200
C	3.70939200	1.36955400	-0.05156700
C	5.52878100	-0.74243400	-0.19424200
H	3.82846900	-2.02154600	-0.28884100
C	5.07164100	1.63186400	-0.02937400
H	3.00318100	2.18705500	0.00324400
C	5.98816200	0.58536300	-0.10284800
O	1.37456300	-1.41222800	-0.25466900
O	7.32506200	0.76912800	-0.10067800
H	7.53650000	1.70637600	-0.02369700
O	6.42703800	-1.76695700	-0.30339400
H	-8.04094800	-1.95652100	0.10773900
C	7.05033000	-2.11622600	0.93658500
H	6.29903200	-2.47792700	1.64339300
H	7.75948300	-2.91080000	0.71456100
H	7.57745300	-1.25803700	1.35828200
H	5.43203900	2.65250900	0.03856700

3dC2"O•

H(UM06-2X)= -1254.145478 Ha
Charge = 0; Multiplicity =2

C	2.88736400	1.71210500	-0.46513900
C	4.77874500	0.22788200	-0.29226900
C	4.02215300	-0.80666100	0.24101200
C	2.58090200	-0.60812500	0.47414900
C	2.03622300	0.69067100	0.12950100
H	6.70247900	0.91344900	-0.93587900
C	6.14563200	0.08445400	-0.51761000
C	4.65104100	-2.01731900	0.55447300
C	6.00710900	-2.17680800	0.33590300
C	6.75178600	-1.11998500	-0.20082800
H	4.04790000	-2.81546100	0.96945000
H	6.49284000	-3.11354900	0.57765600
O	4.22335700	1.42669900	-0.62573000
O	2.55222400	2.80746200	-0.84909200
O	1.90896000	-1.53444300	0.95102300
C	0.66121900	0.97915700	0.33312500
C	0.03584100	2.30333300	0.03697700
H	0.63581900	3.09654900	0.47735700
H	0.03193500	2.46990600	-1.04109800
H	-0.97937400	2.34462100	0.41940900
N	-0.11323700	0.01958000	0.82971100
H	0.32533600	-0.89496200	1.00302600
N	-1.47885800	0.13357800	0.94301800
C	-2.26502000	0.04977200	-0.18382600
H	-1.86208100	-0.04350100	1.86189700
C	-3.72898200	0.09556600	0.10858600
C	-4.59987700	-0.84931900	-0.56290600
C	-4.25715700	1.03541200	0.95138100
C	-6.03541300	-0.71940100	-0.29171600
C	-5.65846600	1.12128400	1.17517400
H	-3.60799900	1.74920300	1.44561000
C	-6.54126000	0.25636700	0.56060300
O	-1.79562300	0.01160800	-1.29908100
O	-6.82932200	-1.58561500	-0.89519600
H	-6.26359000	-2.17172100	-1.43140300
H	7.81448600	-1.24136200	-0.37347200
O	-4.23224800	-1.76564800	-1.31589900
H	-6.03402000	1.88706600	1.84158700
H	-7.60889800	0.31684500	0.72900900

3dC3”O•

H(UM06-2X)= -1254.145955 Ha
Charge = 0; Multiplicity =2

C	-2.81103800	1.76356300	0.23654900
C	-4.73093900	0.30907300	0.31204000
C	-4.01357200	-0.79722600	-0.12290500
C	-2.57911700	-0.65968800	-0.42839300
C	-1.99887200	0.65955200	-0.25903400

H	-6.61784700	1.10641400	0.93648100
C	-6.09067600	0.22243800	0.60041800
C	-4.67505200	-2.02206200	-0.27027900
C	-6.02467200	-2.12532900	0.01251500
C	-6.72961600	-0.99711800	0.44812300
H	-4.10278100	-2.87666600	-0.60986600
H	-6.53490300	-3.07313900	-0.10269500
O	-4.14277200	1.52704000	0.48071100
O	-2.44312100	2.89003200	0.46976300
O	-1.94036600	-1.64939800	-0.81315600
C	-0.62931800	0.89242300	-0.53870900
C	0.03414700	2.22490600	-0.40985300
H	-0.55572000	2.97701400	-0.92946300
H	0.05948600	2.51668500	0.64125600
H	1.04282900	2.19708800	-0.81041000
N	0.10836100	-0.13578700	-0.95258100
H	-0.35029700	-1.05570400	-1.00083400
N	1.47112000	-0.06397100	-1.12190200
C	2.28111300	-0.09152300	-0.02495100
H	1.80748700	-0.28509700	-2.04805500
C	3.74633000	-0.12099000	-0.25870700
C	4.57627500	-0.39288800	0.84449000
C	4.32903900	0.12682800	-1.50275500
C	6.03607400	-0.48389000	0.67600400
C	5.72515800	0.07152000	-1.69043500
H	3.71431700	0.39679100	-2.35236600
C	6.55371300	-0.23148600	-0.64708600
O	1.80393300	-0.06605100	1.10600700
O	6.76069900	-0.75642800	1.64553500
H	-7.78730900	-1.07341400	0.67002700
O	4.13152700	-0.57984100	2.06713200
H	6.13027500	0.27409000	-2.67347200
H	7.62897700	-0.28553400	-0.76915700
H	3.15606200	-0.43167600	2.05980400

3dC7”N•

H(UM06-2X)= -1254.148333 Ha

Charge = 0; Multiplicity =2

C	3.26020900	1.87996200	-0.02710500
C	4.82370300	0.05076100	0.00589700
C	3.83019400	-0.91964000	-0.00377800
C	2.41614000	-0.52232500	-0.01742200
C	2.13861300	0.92430200	-0.01061300
H	6.91777000	0.49155000	0.03114400
C	6.17169100	-0.29277700	0.02395100
C	4.19869700	-2.27141600	0.00350000
C	5.53314300	-2.62939300	0.02087600
C	6.51758100	-1.63416900	0.03131000
H	3.41379400	-3.01764900	-0.00443100
H	5.81783100	-3.67379700	0.02659700

O	4.53338100	1.38523600	-0.00502300
O	3.16525400	3.07896300	-0.06022000
O	1.53300200	-1.37921600	-0.02909700
C	0.83886900	1.43306800	0.00777100
C	0.47277800	2.88136800	0.03687800
H	0.96450800	3.36943400	0.87751800
H	0.84123900	3.36878900	-0.86659100
H	-0.60336000	2.99471900	0.10862200
N	-0.18844200	0.53646500	0.00017500
H	0.06267000	-0.47074900	-0.01761000
N	-1.44279100	0.89704900	0.01119700
C	-2.28876900	-0.21479000	-0.00123100
C	-3.72196900	0.10724700	0.00485900
C	-4.64660800	-0.94731600	-0.00350100
C	-4.18604600	1.43576700	0.01758200
C	-6.02219600	-0.66785400	0.00107200
C	-5.54179300	1.69573000	0.02167500
H	-3.46611800	2.24242500	0.02407900
C	-6.46214400	0.64224100	0.01335900
O	-1.85640800	-1.36889900	-0.01625800
O	-6.91567600	-1.68901000	-0.00663500
H	-6.43264100	-2.52578500	-0.01706800
H	7.56496500	-1.91115600	0.04499400
O	-4.31957900	-2.25510400	-0.01583400
H	-3.34519800	-2.33027900	-0.02037200
H	-5.89988500	2.71699200	0.03122100
H	-7.52927900	0.83055400	0.01634700

3eC3”O•
H(UM06-2X)= -1254.147774 Ha
Charge = 0; Multiplicity =2

C	-2.94819500	1.75506000	0.15384500
C	-4.90784800	0.35228600	0.14142400
C	-4.18349400	-0.79544300	-0.15273500
C	-2.72388900	-0.70824600	-0.33269800
C	-2.12763400	0.60619800	-0.20187000
H	-6.82125700	1.22696200	0.54170600
C	-6.28957200	0.31218200	0.31226500
C	-4.86086700	-2.01374500	-0.27867200
C	-6.23231700	-2.07100500	-0.11138600
C	-6.94364900	-0.90217200	0.18464700
H	-4.28333600	-2.90026800	-0.51030200
H	-6.75494200	-3.01397700	-0.20958000
O	-4.30454200	1.56615600	0.28297900
O	-2.57045800	2.88356100	0.36226800
O	-2.07967500	-1.73586000	-0.58955600
C	-0.73094500	0.79169400	-0.37667500
C	-0.05106700	2.12088200	-0.31581100
H	-0.61248400	2.84731500	-0.89801300
H	-0.04324600	2.47624000	0.71616400

H	0.96787200	2.04688600	-0.68512100
N	0.01720400	-0.27866500	-0.62257900
H	-0.45749300	-1.19131500	-0.63918900
N	1.39103600	-0.24185700	-0.66751000
C	2.09774800	-0.12345400	0.50565700
H	1.80731100	-0.65462900	-1.49085400
C	3.58276400	-0.21840200	0.35974300
C	4.23400300	0.13862000	-0.78977100
C	4.30762600	-0.65995400	1.50335500
C	5.66955000	0.04648800	-0.86349100
H	3.72133100	0.52371400	-1.66368500
C	5.68186000	-0.77717600	1.48735900
H	3.74956100	-0.91001800	2.39700200
C	6.36968400	-0.43439900	0.32785800
O	1.55343400	0.08036700	1.56974600
O	7.68506800	-0.52320800	0.25406100
H	7.94723300	-0.23136900	-0.63821600
O	6.34707800	0.35064900	-1.86424600
H	-8.01828400	-0.94233900	0.31641700
H	6.23026200	-1.12335900	2.35400800

3eC4”O•

H(UM06-2X)= -1254.147128 Ha

Charge = 0; Multiplicity =2

C	-2.93150200	1.75605100	0.16632000
C	-4.89196500	0.35459500	0.14311400
C	-4.16932800	-0.79009300	-0.16685600
C	-2.71039200	-0.70150400	-0.35099600
C	-2.11234700	0.61051900	-0.20378900
H	-6.80346400	1.22548000	0.56061200
C	-6.27298800	0.31313600	0.31901000
C	-4.84772000	-2.00667800	-0.30347300
C	-6.21848900	-2.06527800	-0.13116900
C	-6.92810200	-0.89947900	0.18059300
H	-4.27142200	-2.89085400	-0.54685900
H	-6.74183700	-3.00695800	-0.23740400
O	-4.28767100	1.56664100	0.29557700
O	-2.55261600	2.88187800	0.38648100
O	-2.06774700	-1.72590800	-0.62392800
C	-0.71532300	0.79569300	-0.37364400
C	-0.03202100	2.12182000	-0.28914200
H	-0.58944900	2.85919100	-0.86127500
H	-0.02633800	2.46021900	0.74859200
H	0.98786000	2.05115900	-0.65648300
N	0.03083600	-0.27256300	-0.63654300
H	-0.44618500	-1.18383200	-0.66806300
N	1.40509500	-0.23864500	-0.66895800
C	2.09885900	-0.13693200	0.51387100
H	1.82719200	-0.64495400	-1.49271800
C	3.58369400	-0.23726700	0.38871500

C	4.24647400	0.13543900	-0.76959100
C	4.29069000	-0.69915200	1.53996000
C	5.63255200	0.03228700	-0.80229800
H	3.73054500	0.53371100	-1.63492900
C	5.64503800	-0.81738000	1.52903100
H	3.71513700	-0.95846500	2.41973900
C	6.39727700	-0.45604200	0.35167900
O	1.54347500	0.06036600	1.57374400
O	7.63317800	-0.52051000	0.24410600
O	6.32152100	0.38215200	-1.87296600
H	7.26393700	0.23392500	-1.67056700
H	-8.00219400	-0.94066600	0.31637100
H	6.19714100	-1.17485300	2.38936700

3eC7”N•

H(UM06-2X)= -1254.144254 Ha

Charge = 0; Multiplicity =2

C	-3.17513600	1.92133700	0.06035800
C	-4.94451600	0.29229400	-0.02242500
C	-4.07330100	-0.78876300	-0.00330100
C	-2.62144900	-0.56078400	0.04500900
C	-2.17577200	0.84053800	0.04027900
H	-6.96996600	0.97949100	-0.08852700
C	-6.32304400	0.11158300	-0.07646700
C	-4.59959000	-2.08649500	-0.03911100
C	-5.96637300	-2.28360800	-0.09367500
C	-6.82584400	-1.17868400	-0.11218100
H	-3.90873600	-2.92035000	-0.02454200
H	-6.37195100	-3.28668200	-0.12282400
O	-4.49905200	1.58201000	0.01615300
O	-2.94088500	3.10069300	0.11815800
O	-1.84869600	-1.51841500	0.07616500
C	-0.82227300	1.19084900	0.01857100
C	-0.28967600	2.58608600	-0.03301700
H	-0.74177000	3.12288500	-0.86592700
H	-0.57443300	3.11983900	0.87468000
H	0.79021700	2.57081700	-0.13173600
N	0.09176600	0.18248900	0.03980700
H	-0.27153100	-0.78898000	0.07426200
N	1.38160900	0.38555400	0.01826600
C	2.08053300	-0.83974100	0.05319200
C	3.54912600	-0.69033400	0.02467000
C	4.15714600	0.57250100	-0.02993800
C	4.33585600	-1.84307700	0.05368300
C	5.53429400	0.66439800	-0.05572500
H	3.55352100	1.47197500	-0.05189500
C	5.71877400	-1.74685900	0.02763700
H	3.85396200	-2.81140100	0.09711000
C	6.32328700	-0.49666700	-0.02740000
O	1.50674900	-1.91519500	0.10242600

O	7.66916200	-0.40203200	-0.05392500
H	7.91977500	0.53070100	-0.09799400
O	6.23938500	1.82969500	-0.10813700
H	5.65249300	2.59203300	-0.14995300
H	-7.89776900	-1.32931100	-0.15472100
H	6.34700500	-2.62881300	0.04789100

Anions

3aC2”O¯

H(UM06-2X)= -1179.091182 Ha

Charge = -1; Multiplicity =1

C	-2.50280800	1.74415400	0.33439700
C	-4.45712800	0.33722500	0.22206200
C	-3.73393100	-0.76657800	-0.20913200
C	-2.27890400	-0.64736000	-0.41680200
C	-1.68856900	0.64920800	-0.16636100
H	-6.36557300	1.15022000	0.75556200
C	-5.83463400	0.26868000	0.41876600
C	-4.40777900	-1.96998300	-0.44672000
C	-5.77482300	-2.05556800	-0.25520700
C	-6.48554600	-0.93007300	0.17806400
H	-3.83025000	-2.82290500	-0.78168500
H	-6.29428200	-2.98731000	-0.43973000
O	-3.85646400	1.53223500	0.47936500
O	-2.12855800	2.84866600	0.65396500
O	-1.63795800	-1.63945500	-0.79740100
C	-0.29415500	0.86774000	-0.37124700
C	0.37436500	2.19229100	-0.19206900
H	-0.20712700	2.96805500	-0.68454900
H	0.40528000	2.44021800	0.87003600
H	1.38507300	2.16158900	-0.58888100
N	0.45153900	-0.15448100	-0.75952800
H	-0.01666600	-1.06474700	-0.84865300
N	1.82235700	-0.09153400	-0.86130800
C	2.59316100	-0.09290400	0.30868700
C	4.04517600	-0.15238500	0.05221300
C	4.89617200	-0.93514500	0.92074300
C	4.57223300	0.56599400	-1.03313200
C	6.29647200	-0.88176300	0.58460700
C	5.92797500	0.58593300	-1.31054900
H	3.89807500	1.15296900	-1.65039400
C	6.78477900	-0.15448100	-0.47744800
O	2.04245900	0.01468400	1.38798500
H	-7.55663400	-0.99228200	0.32899200
H	7.85310500	-0.15312600	-0.67298500
H	6.31614200	1.16674500	-2.13726900
H	2.19818200	-0.49794700	-1.70632100
O	4.47249600	-1.64300000	1.88121400
H	6.96924700	-1.45431700	1.21546900

3aC7”N^{-}

H(UM06-2X)= -1179.102250 Ha

Charge = -1; Multiplicity =1

C	-2.87318700	1.90882800	0.03530700
C	-4.53036000	0.15540600	0.00115900
C	-3.56830800	-0.84500100	-0.00745600
C	-2.13663500	-0.48253300	0.00046200
C	-1.81948300	0.91945800	0.00480500
H	-6.61261400	0.66095700	-0.00072500
C	-5.89141600	-0.14676300	-0.00844000
C	-3.98101100	-2.18186700	-0.02555400
C	-5.32756000	-2.49989700	-0.03519400
C	-6.28173400	-1.47577400	-0.02655000
H	-3.21873200	-2.95139600	-0.03234300
H	-5.64409700	-3.53541900	-0.04933300
O	-4.18614700	1.47097400	0.02211200
O	-2.75687400	3.11586300	0.07399800
O	-1.28730800	-1.39251200	-0.00055500
C	-0.44428100	1.36786900	-0.00879600
C	-0.04989500	2.81309200	-0.04763300
H	-0.53798200	3.30837000	-0.88586800
H	-0.39983700	3.31446400	0.85527500
H	1.02880600	2.89530700	-0.12812100
N	0.51464700	0.47513400	0.00651700
H	0.24408200	-0.51940500	0.02316100
N	1.85397200	0.78162500	-0.00556300
C	2.58269400	-0.32096700	0.00965700
C	4.06524000	-0.12925900	-0.00176900
C	4.90660700	-1.25960300	0.01897300
C	4.64814400	1.14249100	-0.03373500
C	6.29612500	-1.09397100	0.00820800
C	6.02467800	1.30551800	-0.04433200
H	3.99206000	2.00420900	-0.05055500
C	6.84791600	0.17630200	-0.02287300
O	2.11681100	-1.50642900	0.03308100
H	-7.33748600	-1.71980300	-0.03381900
H	7.92600600	0.29014300	-0.03090800
H	6.45637500	2.29832300	-0.06979900
H	6.92037100	-1.97984600	0.02410200
O	4.41132900	-2.51173700	0.04952500
H	3.41503600	-2.39245500	0.04952500

3bC4”O^{-}

H(UM06-2X)= -1179.098318 Ha

Charge = -1; Multiplicity =1

C	-2.68926700	1.76287200	-0.02156600
C	-4.60306600	0.31171700	0.19338000
C	-3.85510300	-0.83592300	-0.03235600
C	-2.40774700	-0.72262100	-0.28977900

C	-1.85020600	0.61097000	-0.30438700
H	-6.52482100	1.16368600	0.60251600
C	-5.97449900	0.24737700	0.42993600
C	-4.49711900	-2.07921900	-0.02025800
C	-5.85777800	-2.16100400	0.21376800
C	-6.59364800	-0.99163200	0.43869200
H	-3.90128800	-2.96551500	-0.20088500
H	-6.35354200	-3.12337900	0.22254000
O	-4.03287000	1.54886400	0.19748100
O	-2.34496300	2.91971300	0.05439600
O	-1.74501900	-1.75404000	-0.48585400
C	-0.45932300	0.81742600	-0.55110400
C	0.17792200	2.16673300	-0.63053700
H	-0.41091100	2.81104500	-1.27965100
H	0.17787200	2.62478300	0.35940800
H	1.19744900	2.08136100	-0.99504000
N	0.30785000	-0.24289800	-0.73569300
H	-0.14223400	-1.16340100	-0.65046400
N	1.68008400	-0.16822400	-0.84882000
C	2.42104300	0.03110900	0.33119300
C	3.86582600	-0.12918600	0.20408100
C	4.64473300	-0.13725200	1.37918000
C	4.53929300	-0.27080700	-1.02715000
C	6.00897200	-0.29713600	1.34061000
H	4.13776500	-0.02116200	2.33152500
C	5.90448900	-0.42631100	-1.08455700
H	3.99229100	-0.22771700	-1.96415100
C	6.72499100	-0.45701600	0.09995900
O	1.83551000	0.36001200	1.35192800
O	7.97975500	-0.60759000	0.05237600
H	-7.65983400	-1.05095800	0.62186200
H	6.58664900	-0.30807300	2.25929800
H	6.40304300	-0.52132000	-2.04354700
H	2.04562300	-0.79775000	-1.54905900

3bC7”N⁻

H(UM06-2X)= -1179.109574 Ha

Charge = -1; Multiplicity =1

C	-2.97433400	1.87433800	0.14005700
C	-4.71009800	0.20803800	0.00249900
C	-3.79538900	-0.83627800	-0.01725000
C	-2.34719400	-0.54155200	0.02645300
C	-1.97120400	0.84340100	0.02446600
H	-6.76485500	0.81317400	-0.03555400
C	-6.08296500	-0.02787900	-0.05428000
C	-4.27111100	-2.14994900	-0.09287100
C	-5.63059100	-2.40247700	-0.15045700
C	-6.53555900	-1.33489700	-0.13109200
H	-3.54610700	-2.95463900	-0.10768300
H	-5.99488000	-3.42037600	-0.21097200

O	-4.30727800	1.50392900	0.08995200
O	-2.79645100	3.06590500	0.29229200
O	-1.53941600	-1.48873500	0.04424800
C	-0.57873900	1.23734300	-0.04436000
C	-0.13452700	2.65147700	-0.27010800
H	-0.79877300	3.14839900	-0.97236400
H	-0.18224100	3.21134800	0.66651600
H	0.88926700	2.65155900	-0.63274200
N	0.35052600	0.32222600	0.05459500
H	0.05663500	-0.65966500	0.15790400
N	1.69656300	0.57740400	-0.00534600
C	2.38244700	-0.56610600	0.08648700
C	3.87458200	-0.39849100	0.03617700
C	4.68426900	-1.52992400	0.12248700
C	4.48654700	0.85257700	-0.09714600
C	6.07029800	-1.42735100	0.07873200
H	4.21388000	-2.50040400	0.22497100
C	5.86715000	0.96989700	-0.14130600
H	3.86690000	1.73781100	-0.16612600
C	6.66217900	-0.17333500	-0.05304300
O	1.88850200	-1.71061400	0.20702700
O	8.01272900	-0.00258900	-0.10229300
H	8.45611300	-0.85482700	-0.03452000
H	-7.60088900	-1.52730200	-0.17571500
H	6.69071200	-2.31543500	0.14417900
H	6.34535500	1.93677700	-0.24510600

3cC4”O^{-}
H(UM06-2X)= -1293.572976 Ha
Charge = -1; Multiplicity =1

C	-3.26026100	1.73986300	-0.29550400
C	-5.19990800	0.42060800	0.25990300
C	-4.49539000	-0.77502000	0.21987600
C	-3.06117700	-0.76241700	-0.12300600
C	-2.47277000	0.52484100	-0.41776400
H	-7.07569500	1.40235300	0.58511600
C	-6.55945100	0.45088800	0.56284800
C	-5.16989200	-1.97026700	0.49513200
C	-6.51900700	-1.95781100	0.79936400
C	-7.21114700	-0.74150600	0.83116000
H	-4.60743900	-2.89518400	0.46073100
H	-7.03912400	-2.88326500	1.01159900
O	-4.59762400	1.61662900	0.01356100
O	-2.87633300	2.87871300	-0.42948300
O	-2.43554200	-1.83438800	-0.15361100
C	-1.09970000	0.63062200	-0.79363800
C	-0.44128700	1.91519000	-1.17956700
H	-1.07573300	2.46037700	-1.87414900
H	-0.32363800	2.53935500	-0.29250900
H	0.53267100	1.72146300	-1.61980800

N	-0.37185500	-0.47250500	-0.82174000
H	-0.83393700	-1.34281600	-0.52675900
N	0.98696500	-0.47122000	-1.05412800
C	1.82674900	-0.04781700	-0.00908400
C	3.25509900	-0.26899300	-0.21660900
C	4.12752500	-0.01653400	0.86253500
C	3.82130400	-0.70493300	-1.42824900
C	5.47997700	-0.21411700	0.75435000
H	3.72222100	0.33153000	1.80687500
C	5.17992100	-0.90098400	-1.55102400
H	3.20142000	-0.87085000	-2.30385000
C	6.09834900	-0.68918100	-0.46735300
O	1.33549700	0.50739600	0.96168800
O	7.34042200	-0.89763900	-0.55984800
O	6.27872600	0.00151000	1.85909700
H	-8.26822700	-0.72749500	1.06863600
C	7.08884400	1.16658100	1.73768700
H	7.69585500	1.11935800	0.83154000
H	6.45954900	2.06238100	1.71832600
H	7.73645700	1.19790400	2.61327600
H	5.59872700	-1.23569000	-2.49433500
H	1.28329200	-1.24520500	-1.63120500

3cC7”N^{-}

H(UM06-2X)= -1293.588914 Ha

Charge = -1; Multiplicity =1

C	3.70572500	1.80785500	-0.20144500
C	5.29036500	0.00023000	-0.02549200
C	4.28824500	-0.95854100	0.04461600
C	2.87127300	-0.53831900	0.01164500
C	2.61857800	0.87385200	-0.03486500
H	7.39067000	0.42256200	-0.04742200
C	6.63808200	-0.35382600	0.01362000
C	4.64884600	-2.30612200	0.15379900
C	5.98190100	-2.67551800	0.19385700
C	6.97616400	-1.69271100	0.12319300
H	3.85682200	-3.04318900	0.20759900
H	6.25663800	-3.71942100	0.27938400
O	5.00140300	1.32311100	-0.15020700
O	3.63163600	3.00383400	-0.39820300
O	1.98195600	-1.40899500	0.04250500
C	1.26843800	1.39302600	0.03476600
C	0.95736500	2.84683500	0.22899200
H	1.67646100	3.29913000	0.90679600
H	1.03580000	3.37649300	-0.72297300
H	-0.05490400	2.94793900	0.60919700
N	0.25760500	0.56550600	-0.03453900
H	0.45723200	-0.44170100	-0.11126500
N	-1.05709600	0.95041900	0.02391300
C	-1.85221200	-0.12144500	-0.03238300

C	-3.32163000	0.19477900	0.01801600
C	-4.22409700	-0.87204800	-0.07085800
C	-3.80583000	1.49374800	0.15116000
C	-5.58807000	-0.63335400	-0.02848800
H	-3.82719000	-1.87342300	-0.17187500
C	-5.17697100	1.73501100	0.19393300
H	-3.10695100	2.31671000	0.22235800
C	-6.06917000	0.67901600	0.10434700
O	-1.47547900	-1.31272400	-0.12288900
O	-7.40787400	0.90875100	0.14522800
H	-7.86142300	0.05850200	0.07069400
O	-6.56871200	-1.57794300	-0.10379200
H	8.02144100	-1.97705300	0.15274200
C	-6.16465300	-2.93357700	-0.23248700
H	-5.59324400	-3.07987300	-1.15247500
H	-5.56439300	-3.24009200	0.62768300
H	-7.07921400	-3.51973400	-0.27009000
H	-5.56883700	2.74006800	0.29734600

3dC2”O^{-}

H(UM06-2X)= -1254.321318 Ha

Charge = -1; Multiplicity =1

C	-2.77820600	1.73584900	-0.38532900
C	-4.75283000	0.45544000	0.13703400
C	-4.06888000	-0.75231500	0.13183500
C	-2.62516600	-0.77012100	-0.17060500
C	-1.99771700	0.50859200	-0.43693200
H	-6.61724100	1.47534800	0.40418100
C	-6.11769700	0.51479500	0.40971500
C	-4.77054200	-1.93112300	0.41066800
C	-6.12531100	-1.88989500	0.68596200
C	-6.79604800	-0.66130900	0.68343000
H	-4.22385000	-2.86601300	0.40366700
H	-6.66600100	-2.80233000	0.90306100
O	-4.12593100	1.63646500	-0.11982900
O	-2.37904500	2.86482600	-0.54934800
O	-2.02352800	-1.85405200	-0.18533300
C	-0.60483700	0.59469100	-0.71815000
C	0.10180200	1.87742900	-1.01505300
H	0.08013900	2.51658000	-0.13211300
H	1.13001900	1.68917600	-1.30727800
H	-0.42767800	2.40848300	-1.80396600
N	0.10525700	-0.52672600	-0.73748600
H	-0.38856600	-1.39573100	-0.50486000
N	1.47593500	-0.54970600	-0.83104300
C	2.25697100	-0.16953700	0.25332700
H	1.86684700	-0.99036200	-1.65037800
C	3.69232700	-0.47497800	0.09398100
C	4.66491100	0.46224300	0.54299800

C	4.08414500	-1.70011000	-0.49623600
C	6.04151500	0.06494700	0.33776100
C	5.41119800	-2.03545400	-0.64935800
H	3.32124300	-2.41157900	-0.79580700
C	6.40726900	-1.12804800	-0.22673400
O	1.73259100	0.38185900	1.20485900
O	6.95731400	0.98233900	0.75570500
H	6.39975000	1.71031700	1.09315700
H	-7.85735100	-0.62493600	0.89828300
O	4.46658400	1.60963800	1.06457900
H	5.69255700	-2.98791100	-1.08024700
H	7.46050400	-1.36119000	-0.34294900

3dC3”O¯
H(UM06-2X)= -1254.318248 Ha
Charge = -1; Multiplicity =1

C	-2.85293100	1.70099200	0.48958600
C	-4.75242700	0.22644400	0.31486100
C	-4.00646200	-0.79691100	-0.25363400
C	-2.56754600	-0.59605800	-0.50347500
C	-2.01489300	0.69115400	-0.13797200
H	-6.66437500	0.90014000	1.00495800
C	-6.11619900	0.08016500	0.55825100
C	-4.64289900	-1.99867600	-0.58499000
C	-5.99590800	-2.16089900	-0.34919200
C	-6.72997000	-1.11557800	0.22363100
H	-4.04806100	-2.78804700	-1.02794700
H	-6.48732400	-3.09090300	-0.60518100
O	-4.18853800	1.41549200	0.66636200
O	-2.51108500	2.78856300	0.89140300
O	-1.90672200	-1.51485900	-1.01149400
C	-0.63679300	0.97879300	-0.35150200
C	-0.00599100	2.29817300	-0.04467700
H	-0.61083100	3.09792200	-0.46637100
H	0.01268800	2.44910700	1.03539200
H	1.00521400	2.33876500	-0.43883900
N	0.12965600	0.02762300	-0.86657800
H	-0.31347900	-0.88204400	-1.04966400
N	1.49483500	0.14140700	-0.98407700
C	2.28807300	0.05368700	0.15168700
H	1.87330600	-0.09198300	-1.89145000
C	3.74242500	0.06305800	-0.14463400
C	4.61568400	-0.67953000	0.63297500
C	4.25135600	0.83635000	-1.21621600
C	6.03512800	-0.71268300	0.38180000
C	5.61227100	0.83980000	-1.46321400
H	3.58389800	1.45357300	-1.80509000
C	6.49445100	0.07911600	-0.68573600
O	1.78567900	0.03067400	1.25720800
O	6.74309300	-1.46334000	1.15223100

H	-7.79008400	-1.23909400	0.41014400

3dC7”N¯

H(UM06-2X)= -1254.343943 Ha

Charge = -1; Multiplicity =1

C	3.26642000	1.86452800	-0.05136100
C	4.84428800	0.04017400	-0.00357300
C	3.84036100	-0.91808200	0.01094500
C	2.42562600	-0.49306300	0.00004900
C	2.17047200	0.92241600	-0.00779700
H	6.94568700	0.45815700	-0.00438000
C	6.19121600	-0.31858500	0.00880400
C	4.19652400	-2.27134100	0.03782400
C	5.52853600	-2.64547000	0.05038000
C	6.52521600	-1.66261700	0.03568800
H	3.40241600	-3.00799700	0.04898300
H	5.80086700	-3.69339400	0.07129000
O	4.55798400	1.36903400	-0.03461200
O	3.20309300	3.07471300	-0.10531600
O	1.53685900	-1.36414800	0.00326300
C	0.81827000	1.43255800	0.01173000
C	0.49005700	2.89390200	0.06548000
H	1.01364100	3.36133600	0.89816900
H	0.84697800	3.38527100	-0.84022800
H	-0.58222800	3.02395700	0.16500400
N	-0.18130900	0.58475000	-0.00799500
H	0.04208100	-0.42115200	-0.03061400
N	-1.50416600	0.95684900	0.01017200
C	-2.28751800	-0.10435000	-0.01047900
C	-3.75766200	0.16199100	0.00637500
C	-4.62673500	-0.93405000	-0.01885100
C	-4.29709700	1.45581700	0.04765300
C	-6.01631400	-0.73209000	-0.00341500
C	-5.66850700	1.64606900	0.06261000
H	-3.61939500	2.29944700	0.06795300
C	-6.53529400	0.54804600	0.03676500
O	-1.88676700	-1.31517100	-0.04326200
O	-6.84218200	-1.81488400	-0.02838700
H	-6.28965300	-2.60768300	-0.05729500
H	7.56970000	-1.95124100	0.04495500
O	-4.20558200	-2.21679800	-0.05843300
H	-3.19723500	-2.14201500	-0.06096400
H	-6.07788300	2.64816300	0.09502700
H	-7.61103900	0.67887700	0.04786600

3eC3”O¯

H(UM06-2X)= -1254.323002 Ha

Charge = -1; Multiplicity =1

C	-2.97352200	1.76093700	0.07000800
C	-4.89989600	0.31217200	0.12741100

C	-4.14981500	-0.82622500	-0.13559400
C	-2.69404700	-0.70835400	-0.33499600
C	-2.12830500	0.62067600	-0.24600400
H	-6.82992800	1.15228400	0.52171200
C	-6.27824800	0.24342400	0.31705100
C	-4.79733500	-2.06478700	-0.20984100
C	-6.16480800	-2.15089600	-0.02242200
C	-6.90251100	-0.99077400	0.24147800
H	-4.20012000	-2.94404300	-0.41819700
H	-6.66427500	-3.10969200	-0.08049500
O	-4.32567100	1.54413600	0.21783100
O	-2.62618400	2.90777500	0.22908600
O	-2.02938100	-1.72936500	-0.56966600
C	-0.72989100	0.83067200	-0.41919200
C	-0.08135200	2.17649400	-0.38715500
H	-0.64006200	2.86569800	-1.01619000
H	-0.11662400	2.57167900	0.62911100
H	0.95067000	2.10842200	-0.71882700
N	0.04110500	-0.22448800	-0.63616400
H	-0.41547900	-1.14584000	-0.63118400
N	1.41548300	-0.15779400	-0.65940900
C	2.09914900	-0.00657500	0.54103700
H	1.84812800	-0.65023100	-1.42816000
C	3.57273000	-0.13323100	0.43899600
C	4.25184100	0.06098300	-0.77887000
C	4.26134900	-0.47110100	1.59986800
C	5.64787800	-0.08815100	-0.86767800
H	3.72498600	0.37591100	-1.67370700
C	5.65083100	-0.63746300	1.54899600
H	3.71866200	-0.61648200	2.52485000
C	6.31416600	-0.45258200	0.35493500
O	1.49149200	0.24031000	1.56652500
O	7.66010300	-0.59296500	0.24713400
H	7.82447400	-0.39040400	-0.69550300
O	6.37657400	0.07652500	-1.91555000
H	-7.97405100	-1.05359800	0.38892400
H	6.21276700	-0.91037400	2.43563900

3eC4”O^{-}

H(UM06-2X)= -1254.326808 Ha

Charge = -1; Multiplicity =1

C	-2.98872200	1.76748700	-0.03396400
C	-4.88932600	0.29364700	0.14319100
C	-4.12777700	-0.84345000	-0.08975900
C	-2.67901400	-0.71232400	-0.32942700
C	-2.13171000	0.62604200	-0.30775300
H	-6.82230200	1.12085900	0.55006200
C	-6.26137300	0.21236500	0.37041600
C	-4.75680800	-2.09351300	-0.09589000
C	-6.11791100	-2.19211400	0.12867600

C	-6.86741400	-1.03310500	0.36216000
H	-4.15040200	-2.97148500	-0.28200700
H	-6.60338400	-3.15974500	0.12368500
O	-4.33287500	1.53677000	0.16304100
O	-2.65991000	2.92808700	0.05032800
O	-2.00485200	-1.73373400	-0.53730400
C	-0.73543500	0.84589800	-0.50324000
C	-0.10273600	2.19962100	-0.51745700
H	-0.65587400	2.85241700	-1.18937200
H	-0.16687400	2.63991200	0.47837000
H	0.93694600	2.12859500	-0.82267900
N	0.04386900	-0.20545000	-0.69473700
H	-0.40431000	-1.13020400	-0.65078000
N	1.41948500	-0.12242800	-0.72827200
C	2.09192400	0.04593100	0.49258600
H	1.83344800	-0.71247600	-1.43603900
C	3.54760000	-0.09189300	0.43110600
C	4.27441900	-0.11987700	-0.78609400
C	4.24851300	-0.20922500	1.63592900
C	5.63400900	-0.27408900	-0.75965200
H	3.79213000	0.01652100	-1.74850700
C	5.62511100	-0.36844600	1.66276500
H	3.68678100	-0.18582400	2.56298900
C	6.38690000	-0.40897000	0.46597100
O	1.45252300	0.32851500	1.49365200
O	7.65095600	-0.54373900	0.37784900
O	6.38167200	-0.29038500	-1.89971200
H	7.29297700	-0.39931400	-1.57070100
H	-7.93390400	-1.10584000	0.53870400
H	6.14968200	-0.46630500	2.60713500

3eC7”N^{-}
H(UM06-2X)= -1254.331798 Ha
Charge = -1; Multiplicity =1

C	-3.17724100	1.88690700	0.17895800
C	-4.98162300	0.30011600	-0.00168400
C	-4.11355100	-0.78319900	-0.03256900
C	-2.65404500	-0.55194000	0.02679000
C	-2.21959100	0.81594300	0.04498300
H	-7.00651500	0.99754300	-0.04899200
C	-6.36278100	0.12706200	-0.07603300
C	-4.64684200	-2.07291400	-0.13663600
C	-6.01557300	-2.26317800	-0.21153400
C	-6.87247900	-1.15668400	-0.18141400
H	-3.95848800	-2.90898900	-0.15942800
H	-6.42406900	-3.26258200	-0.29458300
O	-4.52418300	1.57509000	0.11586800
O	-2.94835500	3.06579900	0.35904400
O	-1.88715200	-1.53221900	0.03670600
C	-0.81265800	1.15196400	-0.02100300

C	-0.30960700	2.54588600	-0.24925000
H	-0.96973700	3.08117100	-0.92633000
H	-0.29938100	3.09829100	0.69314200
H	0.70196200	2.49986700	-0.64277200
N	0.07837900	0.19968700	0.07945600
H	-0.25418300	-0.76951300	0.18128800
N	1.43298900	0.40183800	0.02037800
C	2.07479700	-0.76673600	0.11212600
C	3.57304500	-0.65269700	0.06297300
C	4.21195700	0.58557300	-0.07313200
C	4.34399100	-1.80829900	0.15477900
C	5.59209900	0.65253300	-0.11423600
H	3.62109300	1.49161900	-0.14593800
C	5.73449600	-1.74128500	0.11228900
H	3.84387500	-2.76253400	0.25988700
C	6.36369400	-0.51367600	-0.02212200
O	1.53991700	-1.89226000	0.23183700
O	7.72075200	-0.44176800	-0.06506900
H	7.97836700	0.48366000	-0.16427400
O	6.31087200	1.80959800	-0.24295300
H	5.72495600	2.57010400	-0.31474600
H	-7.94478200	-1.30080400	-0.23986000
H	6.34401100	-2.63469300	0.18057600

Radical cations

3a$^{\bullet+}$
H(UM06-2X)= -1179.307568 Ha
Charge = 1; Multiplicity =2

C	-2.44165300	1.69982000	-0.32111500
C	-4.45527600	0.45341900	0.01705700
C	-3.79698600	-0.76212500	0.19215000
C	-2.34746600	-0.83289300	0.08122000
C	-1.66357900	0.43420500	-0.29944500
H	-6.31480300	1.50463800	-0.04349000
C	-5.83750000	0.54339200	0.09561900
C	-4.55083400	-1.91650300	0.45823200
C	-5.92539400	-1.84042600	0.54110100
C	-6.56439600	-0.60755600	0.35674200
H	-4.02755100	-2.85497900	0.59222600
H	-6.50872500	-2.72809600	0.74729800
O	-3.78458500	1.62570000	-0.21820000
O	-1.93579100	2.78330400	-0.40370300
O	-1.72575000	-1.87812300	0.24441500
C	-0.27597600	0.48400600	-0.64203100
C	0.41047900	1.65301600	-1.26006800
H	-0.25248000	2.11444500	-1.98852700
H	0.65927400	2.39595800	-0.50188400
H	1.32358000	1.32100100	-1.75247300
N	0.37944300	-0.65114800	-0.44536000

H	-0.20010200	-1.47552300	-0.14713900
N	1.70533700	-0.80039300	-0.57993200
C	2.58194500	0.04460800	0.16075800
H	1.97881100	-1.77052700	-0.70257900
C	4.00651000	-0.23668900	-0.00534300
C	4.91388400	0.32844300	0.91768300
C	4.49506900	-1.04205900	-1.05045300
C	6.27610600	0.03733000	0.80103300
C	5.84249700	-1.30892000	-1.16467000
H	3.81450100	-1.43291900	-1.79766100
C	6.72948600	-0.77065900	-0.22347200
O	2.10725600	0.90757700	0.86796000
H	-7.64402100	-0.54655600	0.42027700
H	7.78956000	-0.98025200	-0.30295500
H	6.20889000	-1.92215100	-1.97687200
H	6.95596300	0.46818800	1.52537300
O	4.54465200	1.13337900	1.92418700
H	3.59589600	1.33041600	1.85819800

3b$^{\bullet+}$
H(UM06-2X)= -1179.308416 Ha
Charge = 1; Multiplicity =2

C	-2.65242600	1.73999700	-0.15170200
C	-4.62856200	0.39845800	0.04040300
C	-3.93141000	-0.80660600	0.08806900
C	-2.47949400	-0.81311200	-0.01718600
C	-1.82889500	0.50695300	-0.21646100
H	-6.52380900	1.38653400	0.08212000
C	-6.01401400	0.43271600	0.12110200
C	-4.64650900	-2.00722800	0.22155400
C	-6.02329300	-1.98671600	0.30410100
C	-6.70248100	-0.76283200	0.25216600
H	-4.09141300	-2.93633700	0.25580400
H	-6.57702100	-2.91052300	0.40748500
O	-3.99703000	1.60784100	-0.07355200
O	-2.19859800	2.84943700	-0.14459300
O	-1.83382800	-1.86091100	0.00970300
C	-0.45159200	0.63317100	-0.47526600
C	0.24705500	1.88254000	-0.89548000
H	-0.38084600	2.42100300	-1.60144500
H	0.42943500	2.53113900	-0.03871300
H	1.19561300	1.63444800	-1.36976500
N	0.23112700	-0.52660500	-0.41545300
H	-0.37159300	-1.39364700	-0.29616500
N	1.54829900	-0.63935800	-0.45259100
C	2.39014100	0.16814600	0.41437200
H	1.85926400	-1.58643700	-0.65347500
C	3.82306100	-0.06236200	0.22609100
C	4.68035500	0.34861600	1.25369100
C	4.35452300	-0.64185900	-0.93384200

C	6.04448400	0.17068300	1.13709800
H	4.26231700	0.79962300	2.14533400
C	5.71873100	-0.81384800	-1.06039000
H	3.71440200	-0.93288600	-1.75880200
C	6.56639600	-0.41363000	-0.02275900
O	1.86599700	0.90904800	1.19808900
O	7.88494700	-0.61456100	-0.19865000
H	8.38497000	-0.30310900	0.56471400
H	-7.78360900	-0.74457600	0.31618200
H	6.70971600	0.47984400	1.93528300
H	6.14826300	-1.24867800	-1.95359600

3c$^{\bullet+}$

H(UM06-2X)= -1293.788147 Ha

Charge = 1; Multiplicity =2

C	-3.18066400	1.65402900	-0.57205200
C	-5.18171200	0.49009400	0.04635300
C	-4.51810500	-0.69160600	0.36832200
C	-3.07454500	-0.78519600	0.20396500
C	-2.40006800	0.40935400	-0.36345200
H	-7.04299400	1.53440500	-0.07970400
C	-6.55940400	0.60081100	0.17636100
C	-5.25960100	-1.78939900	0.83254000
C	-6.62900700	-1.69219700	0.96638100
C	-7.27433000	-0.49375000	0.63595900
H	-4.73063300	-2.70212800	1.07663100
H	-7.20372100	-2.53702600	1.32244600
O	-4.52200400	1.60463300	-0.39755700
O	-2.69432800	2.70900900	-0.86764800
O	-2.45855300	-1.81648600	0.47389200
C	-1.04169400	0.40118500	-0.72639600
C	-0.33848500	1.46730600	-1.49752000
H	-0.99966800	1.83418500	-2.27922600
H	-0.07924600	2.30581000	-0.85119800
H	0.56764200	1.06362700	-1.94739000
N	-0.39099400	-0.73687200	-0.41152500
H	-1.01016500	-1.51374100	-0.03364300
N	0.91505100	-0.91902900	-0.50160700
C	1.84381600	0.04561300	0.06162300
H	1.18573400	-1.89909700	-0.47475900
C	3.24977500	-0.28819200	-0.16861600
C	4.19286800	0.35145100	0.65467200
C	3.65616200	-1.16556300	-1.17336900
C	5.53350100	0.09071000	0.47152200
H	3.84748000	1.03147100	1.42199900
C	5.00826100	-1.41751700	-1.36218300
H	2.93874600	-1.63040000	-1.83856500
C	5.94501100	-0.80158900	-0.54578200
O	1.40445900	0.98440600	0.66489300
O	7.25148700	-1.04963500	-0.72176700

H	7.75910700	-0.54074800	-0.07317900
O	6.55662800	0.61297800	1.18385300
H	-8.34977800	-0.41544600	0.73998000
C	6.23897000	1.53141000	2.22543900
H	5.72430200	2.40469200	1.81871800
H	5.61845100	1.04829800	2.98351700
H	7.18818800	1.83102200	2.66036800
H	5.35215200	-2.08459400	-2.14201900

3d$^{•+}$
H(UM06-2X)= -1254.546071 Ha
Charge = 1; Multiplicity =2

C	-2.81764400	1.75163500	-0.28269600
C	-4.77190100	0.42741200	0.19719300
C	-4.07748200	-0.77368900	0.13896100
C	-2.63832700	-0.76646700	-0.17114500
C	-2.02958500	0.53042500	-0.41485200
H	-6.64483700	1.41722900	0.50954400
C	-6.13650800	0.46208900	0.47179000
C	-4.76765500	-1.97041800	0.36624700
C	-6.12241500	-1.95321800	0.64181500
C	-6.80378100	-0.73150200	0.69267500
H	-4.21302100	-2.89942700	0.31815300
H	-6.65489100	-2.87913400	0.81766000
O	-4.15606300	1.62724000	-0.00356000
O	-2.41809200	2.88569900	-0.38809700
O	-2.01783500	-1.83710600	-0.22130300
C	-0.66417200	0.63673700	-0.75934700
C	0.02625200	1.92578600	-1.06346700
H	0.09045800	2.52422600	-0.15319700
H	1.02070300	1.75403700	-1.46437000
H	-0.56639300	2.49800700	-1.77375800
N	0.04907900	-0.49028800	-0.83233200
H	-0.42780700	-1.37227400	-0.59405000
N	1.40577100	-0.51513200	-1.05035900
C	2.25217100	-0.19812800	-0.05420900
H	1.71115500	-0.90006100	-1.93346900
C	3.71541800	-0.34718700	-0.28127200
C	4.54463100	0.03937500	0.80684600
C	4.32044400	-0.82055900	-1.41988400
C	5.98699100	-0.06695500	0.69126900
C	5.74215800	-0.92196300	-1.52216400
H	3.74042700	-1.13138500	-2.27976900
C	6.56442900	-0.55390200	-0.49390400
O	1.83797600	0.23772600	1.02491100
O	6.73607200	0.28937400	1.69453900
H	6.20676800	0.60341900	2.44922400
H	-7.86542800	-0.71405700	0.90793600
O	4.09422400	0.49094700	1.92816300
H	6.16451300	-1.30162400	-2.44301600

H	7.64204500	-0.62552900	-0.55866700
H	3.06395200	0.49666100	1.83624600

3e$^{\bullet+}$

H(UM06-2X)= -1254.536132 Ha

Charge = 1; Multiplicity =2

C	-3.01662300	1.72564200	-0.34715500
C	-4.94827100	0.33981800	0.04466400
C	-4.20296200	-0.83102700	0.09177500
C	-2.74431800	-0.77335300	-0.10453600
C	-2.17061900	0.53768300	-0.35292800
H	-6.87824200	1.25839400	0.17562700
C	-6.32992900	0.32579700	0.21811400
C	-4.85899700	-2.04638900	0.31948200
C	-6.23021100	-2.07764800	0.49478700
C	-6.96265900	-0.88580000	0.44254100
H	-4.26476200	-2.95126200	0.35222000
H	-6.73644600	-3.01815100	0.67090500
O	-4.36760200	1.55513900	-0.16614200
O	-2.65984800	2.87157100	-0.47881400
O	-2.08037200	-1.81774500	-0.05855200
C	-0.78203100	0.69197000	-0.57387300
C	-0.12621700	1.99923600	-0.87908100
H	-0.67731800	2.50941500	-1.66573900
H	-0.16641800	2.63910900	0.00376700
H	0.90655700	1.85828900	-1.18330300
N	-0.01289500	-0.39812200	-0.52392600
H	-0.46934000	-1.29030800	-0.29472200
N	1.35916400	-0.36377200	-0.58249000
C	2.07042600	0.12990200	0.46871600
H	1.78999900	-0.63542700	-1.45664000
C	3.56460600	0.04305600	0.32844800
C	4.18180900	-1.02319700	-0.27324300
C	4.31626100	1.11370400	0.92064700
C	5.58416900	-1.02982800	-0.32690200
H	3.63165100	-1.86146700	-0.68448400
C	5.67781900	1.13578000	0.87206400
H	3.76786000	1.91051100	1.40702400
C	6.34575900	0.06738200	0.24570800
O	1.56346300	0.62675200	1.44900900
O	7.64353400	0.05636900	0.18263300
H	7.98459500	-0.73543600	-0.26964800
O	6.32876700	-1.96006300	-0.86224900
H	5.82995100	-2.69559200	-1.25104700
H	-8.03718800	-0.90566000	0.57923100
H	6.26361500	1.93827300	1.29927800
